# Examining the Influence of Early Life Stress on Serum Lipid Profiles and Cognitive Functioning in Depressed Patients

**DOI:** 10.3389/fpsyg.2019.01798

**Published:** 2019-08-06

**Authors:** Ágnes Péterfalvi, Nándor Németh, Róbert Herczeg, Tamás Tényi, Attila Miseta, Boldizsár Czéh, Maria Simon

**Affiliations:** ^1^Neurobiology of Stress Research Group, Szentágothai Research Centre, University of Pécs, Pécs, Hungary; ^2^Department of Laboratory Medicine, Medical School, University of Pécs, Pécs, Hungary; ^3^Bioinformatics Research Group, Szentágothai Research Centre, University of Pécs, Pécs, Hungary; ^4^Department of Psychiatry and Psychotherapy, Medical School, University of Pécs, Pécs, Hungary

**Keywords:** adverse childhood experience, childhood adversity, cardiovascular risk, cholesterol, depression, HDL, major depressive disorder, triglyceride

## Abstract

**Background:**

Early childhood adversity is a strong predictor of the development of major depressive disorder (MDD), but not all depressed patients experience early life stress (ELS). Cardio-metabolic diseases and cognitive deficits often coincide in MDD and worsen its course and outcome. Adverse childhood experiences have been associated with elevated risk for cardiovascular disease (CVD), but little is known on the impact of ELS on cardiovascular risk factors in MDD. Here, we examined MDD patients with and without ELS to explore the effects of ELS on serum lipid and lipoprotein levels and on cognitive performances of the patients.

**Methods:**

Participants with a mean age of 35 years (18–55 years) were recruited from the university mental health clinic and general community. Three groups, matched in age, gender and lifestyle were examined: MDD patients with ELS (*n* = 21), MDD patients without ELS (*n* = 21), and healthy controls (*n* = 20). The following CVD risk factors were assessed: serum lipids (total cholesterol, triglycerides, high- and low-density lipoproteins), body mass index and exercise in a typical week. MDD severity was measured by the Beck Depression Inventory. Childhood Trauma Questionnaire was used to assess early life adversities. Executive functions and attentional processes were assessed by the Wisconsin Card Sorting and Conners’ Continuous Performance tests.

**Results:**

Major depressive disorder patients with ELS had higher serum triglyceride and lower HDL-cholesterol concentrations compared to MDD patients without ELS. Linear regression analysis revealed that the severity of ELS had a significant negative association with HDL-cholesterol levels and significant positive associations with the serum levels of TG and TC/HDL-cholesterol index. We also found significant associations between some specific trauma types and lipid profiles. Finally, we could detect significant associations between depression severity and specific domains of the cognitive tests as well as between lipid profiles and certain domains of the Wisconsin Card Sorting Test. However, we could not detect any association between the severity of ELS and cognitive performance.

**Conclusion:**

After controlling for depressive symptom severity and lifestyle variables, ELS was found to be a strong predictor of serum lipid alterations. Several, inter-correlated pathways may mediate the undesirable effects of ELS on the course and outcome of MDD.

## Introduction

Major depressive disorder is a key public health concern today (Kessler, 2012), as it is a commonly occurring and an often recurring condition associated with considerable functional impairments, diminished quality of life, increased medical morbidity, and mortality (Kessler and Bromet, 2013). MDD often coincides with somatic illnesses such as metabolic syndrome (Pan et al., 2012) and CVD (Hare et al., 2014); nevertheless, the direction of the causal relationship between depression and cardio-metabolic diseases, as well as the specific underlying mechanisms, have not yet been fully understood. Moreover, patients suffering from MDD often present neurocognitive deficits (Austin et al., 2001; McDermott and Ebmeier, 2009; Lee et al., 2012; McIntyre et al., 2013; Rock et al., 2014).

Major depressive disorder is a clinically heterogeneous disorder, which is a result of manifold etiological factors, as well as developmental pathways. ELS, such as adverse childhood experiences (ACEs) (e.g., physical, emotional, and sexual abuse, neglect, parental loss, and poverty), have long been known to be strong predictors of MDD in adulthood (e.g., Widom et al., 2007; Norman et al., 2012; Lindert et al., 2014). A recent meta-analysis of 26 studies revealed that childhood emotional abuse and neglect showed the strongest association with depression risk in adults, while sexual/physical abuse or family violence have been proved to be non-specific risk factors for various mental disorders (Mandelli et al., 2015). Adult MDD with prior ELS is associated with earlier onset, more severe symptomatology, a greater number and longer duration of depressive episodes, a tendency to be chronic or therapy-resistant, higher rates of psychiatric comorbidities, as well as suicidal behavior or impulsivity compared to MDD without ELS (Brodsky et al., 2001; Zlotnick et al., 2001; Klein et al., 2009; Wiersma et al., 2009, 2010; Miniati et al., 2010; Nanni et al., 2012). Moreover, ELS is also a risk factor for severe metabolic alterations and central obesity (Pervanidou and Chrousos, 2012; Davis et al., 2014) and CVD (Rich-Edwards et al., 2012; Loria et al., 2014). Furthermore, a recent study which analyzed data on cardio-metabolic markers of 9000 cohort members found that physical and sexual abuse was associated with high LDL-C and low serum levels of HDL-C, and that childhood neglect, as well as emotional abuse, was associated with raised TG and lower HDL-C (Li et al., 2019). In sum, ELS appears to be related to adult cardio-metabolic complications and comorbidities by two etiologic mechanisms: (1) the direct effect of early and late life stress; (2) general factors that are compensatory behaviors, as well as attempts at self-help by food and agents (Kesebir, 2014).

Serum lipid concentrations have been widely investigated in MDD, however, studies yielded inconsistent results. Both higher (Ledochowski et al., 2003; Nakao and Yano, 2004; Moreira et al., 2017) and lower serum TC levels (Olusi and Fido, 1996; Maes et al., 1997; Ong et al., 2016) were registered in patients with MDD compared to controls, and null findings have also been reported (Lehto et al., 2008; van Reedt Dortland et al., 2010; Enko et al., 2018). Alterations of serum concentrations of LDL-C were most widely studied in MDD. Recently, a comprehensive meta-analysis found significantly lower cross-sectional LDL-C serum concentrations in MDD compared to HCs, when LDL-C was modeled as a continuous measure (Persons and Fiedorowicz, 2016). The authors suggested a U-shaped relationship between depression severity and LDL-C. Nevertheless, this meta-analysis did not consider the effect of ELS on LDL-C concentration in depression. Studies that investigated the relationship between HDL-C and MDD had produced contradictory findings. Some studies found no association at all (Aijänseppä et al., 2002; Rice et al., 2010), while others revealed a correlation between lower HDL-C and depression (Kim et al., 2004; Ancelin et al., 2010) and one study reported higher HDL-C than matched controls (Olusi and Fido, 1996). Similarly, contradictory findings have been published in serum triglyceride levels in depressed patients. Kinder and co-workers reported on a positive correlation between triglyceride blood levels and depression in women aged between 17 and 39 years (Kinder et al., 2004), and a positive correlation between triglyceride blood levels and the BDI score was also found in women who had received coronary angiography (Vaccarino et al., 2008). But there are also negative findings demonstrating no difference in serum TG levels between control and depressed subjects (Pjrek et al., 2007). A number of theories have been put forward to explain the contradictory findings on serum lipid disturbances in depression. Most of them emphasize the influence of the methodology used for the clinical evaluation of depression (e.g., dimensional or categorical assessment), or the impact of demographic, lifestyle and clinical variables (van Reedt Dortland et al., 2010). Furthermore, some results imply that the inconsistent findings might be due to the heterogeneity of the illness and that the lipid disturbances may be characteristic for only certain specific subgroups within the MDD.

So far, only a few studies considered the role of ELS in the association between depression and metabolic disturbances. McIntyre et al. (2012) found a significantly lower level of HDL-C in depressed patients who experienced childhood adversity, but there was no statistically significant difference in the overall rate of dyslipidemia and metabolic syndrome between subjects with and without childhood adversity. Ding et al. (2014) did a metabonomic analysis and reported that MDD patients had lower TC levels compared to controls, but patients with ELS had higher TC levels compared to the MDD only group. Wingenfeld et al. (2017) conducted a women-only study in a physically healthy clinical sample and found no difference in TG, cholesterol, HDL-C, LDL-C and other metabolic risk markers between MDD patients with and without sexual or physical abuse. However, one should carefully interpret these null findings, as the exclusion of obese individuals (with body mass index > 30 kg/m^2^) might have led to an underrepresentation of subjects with existing obesity linked to ELS. More recently Deschênes et al. (2018) reported that ACEs are indirectly associated with diabetes via depressive symptoms and cardio-metabolic dysregulations. While Kraav et al. (2019) found decreased serum TC in depressed outpatients with a childhood history of physical violence. Importantly, most of these earlier studies – when they carried out the statistical analysis of their data – did not control for the effects of ELS, while it is well-known that the prevalence of ACEs is much higher in depressed patients compared to the general population, thus, the presence of ELS might be a confounding variable influencing the outcome of these investigations.

Psychodynamic factors, such as the loss of “good self” or “damaged self” might also have a significant impact. Individuals with ELS experience a defective or “wounded” self, and distressing feelings of shame originating from the internalization of bad or unworthy parents. According to the object relation theory, stressful life events can distort the mental representations of the self and others. This can significantly influence the individual’s behavior, i.e., his or her affective states and self-care. Moreover, the damaged self can negatively impact health behavior and the adaptation to emerging somatic illnesses as well (Kohut, 1977; Ulman and Brothers, 1988; Marchini et al., 2018). Recent psychodynamic theories focus on the role of the attachment and attachment-based mentalizing capacities in the etiology and treatment of depressive disorders, and in the development of somatic disorders in individuals with ELS. Adopting a developmental approach, Luyten and Fonagy (2018) emphasized that ELS can lead to insecure attachment that impairs adaptation to stressful social situations and disrupts the regulation of the stress response. If social stress emerges, hypermentalizing and hypomentalizing can occur on the basis of the insecure attachment. These can lead to deficits of stress regulation, and to dysfunctional compensatory strategies (e.g., drug abuse, self-harm, sexual promiscuity, risk-taking, eating disorders). Due to the unhealthy behavior and the neurobiological changes as a result of ELS, MDD patients with ELS may suffer from stress-related cardiovascular and metabolic diseases more often.

In the present study, we hypothesized that serum lipid levels might be determined by ACEs in depressed patients, based on the following observations: (i) ELS can result in serum lipid alterations both in psychiatric (McIntyre et al., 2012; Misiak et al., 2015) and non-psychiatric samples (van Reedt Dortland et al., 2012; Spann et al., 2014); (ii) lipid disturbances were detected mostly in depressed patients with atypical or melancholic symptoms, or suicidal tendencies, which are more characteristic to depression with ELS (Harkness and Monroe, 2002; Matza et al., 2003; Klein et al., 2009). To investigate this hypothesis, we measured serum lipid and lipoprotein profiles in MDD patients with high and low ELS scores, and in age- and gender-matched HCs. Atherogenic indices (TC/HDL-C, LDL-C/HDL-C) and BMI was also calculated and we collected sociodemographic and clinical data on the participants’ lifestyle as well. Finally, we used two well-established neuropsychological tests to measure the participant’s executive functions (Wisconsin Card Sorting Test) and their attentional processes (Conners’ Continuous Performance Test-II).

## Materials and Methods

### Participants

Forty-two patients with MDD and 20 healthy controls (HCs) participated in this study. Patients with MDD were recruited from the Affective Disorder Unite of the Department of Psychiatry and Psychotherapy, University of Pécs, Hungary. The local Research Ethics Committee of the University of Pécs approved the study design and protocol (Ethical Approval Nr.: 2015/5626) and all participants provided written informed consent. To exclude the effects of aging, only subjects aged between 18 and 55 were involved in the study, because several studies reported an increased prevalence of dyslipidemia in the elderly population (Bechtold et al., 2006; Shanmugasundaram et al., 2010; Liu and Li, 2015).

All patients fulfilled the DSM-5 diagnostic criteria of MDD (American Psychiatric Association, 2013). Inclusion criteria of the MDD group included: (1) age 18–55 years; (2) a diagnosis of MDD in a current major depressive episode as assessed by a trained psychiatrist using the Structured Clinical Interview for DSM-5, Clinical Version, (SCID-5-CV) (First et al., 2015, 2016b) and the Structured Clinical Interview for DSM-5, Personality Disorders (SCID-5-PD) (First et al., 2016a, 2018). Exclusion criteria for the patient group were: current substance abuse or dependence (if the patient met diagnostic criteria, he or she had to be abstinent for at least 2 years), bipolar disorder, post-traumatic stress disorder, a history of any psychotic disorder, and current eating disorders. HC participants were recruited by online advertisements and via personal contacts of the researchers. The control sample was screened by a qualified psychiatrist to ascertain the absence of lifetime or family history of mental disorders. In addition, SCL-90 (Derogatis, 1977) was applied to rule out relevant subthreshold psychiatric symptoms in potentially healthy individuals. Exclusion criteria for both the patients and the controls were: liver or kidney disease, severe CVD, uncontrolled thyroid disorders, uncontrolled diabetes mellitus, and current inflammatory illness. Subjects with known familial hyperlipidemia were not included. Subjects with neurological disorders, in addition, those with a history of head injury and with severe hearing or visual impairment, and an IQ < 85 were also excluded.

In the MDD group, treatment with antidepressant medication or psychotherapy were not exclusion criteria once the diagnosis had been established. Current psychotropic medication data were collected: 41 (97.6%) MDD subjects were taking antidepressants (20 patients were taking SSRIs, 12 mirtazapine, 2 mianserine, 2 venlafaxine, 1 duloxetine, 1 trazodone, 1 vortioxetine, 1 agomelatine), 21 (50%) low dose antipsychotics (17 quetiapine, 1 ziprasidone, 1 aripiprazole, 1 thiothixene), 5 (11.9%) mood stabilizing medications. None of the control subjects took psychotropic medication.

One MDD patient was on lipid-lowering drug (atorvastatin) treatment at the time of the study. Two patients and two control subjects kept a vegetarian diet.

### Laboratory Analyses

Cubital venous blood was drawn from the participants between 7 and 8 AM in order to avoid any possible effect of circadian variations. The samples were collected following 8–12 h of fasting. Serum concentrations of TC, LDL-C, HDL-C, and TG were all measured with a Roche Modular (module P800) clinical chemistry analyzer, using enzymatic colorimetric test methods according to the manufacturer’s instructions (Roche Diagnostics, Hungary).

### Questionnaires

#### Beck Depression Inventory

The severity of actual depressive symptoms was assessed using the BDI (Beck et al., 1961; Hungarian adaptation: Petö et al., 1987; Rózsa et al., 1998). This is a 21-item self-report questionnaire rating the presence and extent of sadness, pessimism, past failure, loss of pleasure, self-dislike, self-criticism, and suicidal thoughts and wishes in the past week. The scores range from 0 to 63 points and higher scores indicate more severe depression. In this study, Cronbach’s alfa values were excellent for the total BDI scores (0.95) and for the cognitive subscale scores (0.91), and good for the somatic-affective subscale (0.86).

#### Childhood Trauma Questionnaire-Short Form

Early life stress was surveyed with the 28-item retrospective self-report questionnaire of the Childhood Trauma Questionnaire-Short Form (CTQ) (Bernstein et al., 2003), that assesses the severity of five types of maltreatment before the age of 18 years: physical abuse (CTQ PA), emotional abuse (CTQ EA), physical neglect (CTQ PN), emotional neglect (CTQ EN), and sexual abuse (CTQ SA). Each subscale is measured with five 5-point scale items. The short form of the questionnaire is the most widely used version, which includes clinical cut-offs for significant abuse and neglect. Childhood maltreatment exposure was entered in the statistical analyses as a continuous variable with row scores, or it was coded into a two-level variable for dividing the MDD sample into low-ELS and high-ELS subgroups. Patients with MDD were assigned to the *MDD Only* subgroup if they had not experienced any types of moderate to severe childhood trauma. MDD patients were put into the *MDD* + *ELS* subgroup if they had at least one type of moderate to severe childhood trauma. In the present sample, the internal consistencies were excellent for the CTQ total score, and for the subscales of physical abuse, emotional abuse, sexual abuse, as well as for emotional neglect (Cronbach’s alphas > 0.9). The internal consistency was acceptable for the subscale of physical neglect (Cronbach’s alpha = 0.77). The Hungarian translation of the original (English) CTQ was done using the back-translation procedure (Sperber, 2004). Two senior authors (MS and BC) translated the English version to Hungarian. To ensure that the translated version is equivalent with the source version a bilingual linguist translated the early Hungarian version back to English. Errors of meaning and concept inconsistencies between the translated versions were discussed and corrected.

### Sociodemographic Data on BMI and Lifestyle

A self-report questionnaire determined the various sociodemographic data, including education, lifestyle habits of regular exercise. Measurements for height and body mass were obtained using a wall-mounted stadiometer and electronic scale, respectively. BMI was calculated as body mass in kilograms divided by height in meters squared.

### Neurocognitive Tests

#### Wisconsin Card Sorting Test

Executive functions were assessed by the computerized version of the WCST (Heaton, 1981). In the test, cards with geometric shapes (different in their number, color, and form) have to be matched according to varying sorting principles. The actual method of sorting has to be found out by the subject based on the provided feedback (correct or incorrect). Besides the number of total correct responses and non-perseverative errors, we detected the number of perseverative errors and conceptual level responses as a measure of shifting ability and conceptual ability, respectively. The WCST is a commonly used cognitive measure in clinical investigations including the studies examining cognitive changes related to depression (see e.g., Li et al., 2010; Giel et al., 2012; McGirr et al., 2012, etc.). Moreover, the WCST has been found to be a highly reliable test already decades ago (e.g., Tate et al., 1998).

#### Conner’s Continuous Performance Test-II

Attentional processes were assessed by the CPT-II (Conners, 2000). In this task, respondents are required to press the space bar when any letter except X appears. The inter-stimulus intervals are variable (1, 2, or 4 s) with display time of 250 ms. There are six blocks, with three sub-blocks each containing 20 trials. The procedure takes 14 min to complete. Omission errors and commission errors, as well as hit reaction time and detectability (a measure of the difference between the signal [non-X] and noise [X] distributions), were assessed.

Conner’s Continuous Performance Test-II is one of the most widely used, computer-administered cognitive test of attention and impulsivity. Since it is not a verbal test, and no language adaptation is necessary thus, the reliability testing of this test was out of the scope of our study. A recent publication reported that CPT-II has a strong internal consistency, adequate test-retest reliability for commission errors and response time, and a relatively poor test-retest reliability for omission errors, and practice effects for omission and commission errors (Shaked et al., 2019). Moreover, CPT-II performances were unrelated to those in other cognitive tests, such as Stoop Color-Word test (Shaked et al., 2019). CPT-II is often used in clinical research on depression (see e.g., Godard et al., 2011; Parlar et al., 2016).

Since none of the clinical studies listed above (using either the CPT-II or the WCST) investigated the reliability of these cognitive tests, we followed the examples of the literature and assumed that both CPT-II and WCST were sufficiently reliable tests.

### The Sequence of Data Collection

Research participants underwent the following study procedures. First, the clinical interviews and questionnaires were completed to assess the severity of depression and ELS. Then, a senior clinician blinded to the results of the CTQ data conducted a semi-structured interview about the stressful early life-events during childhood and adolescence. CTQ sores and the interview responses were compared, discrepancies were discussed with the participants. In the case of unresolvable discrepancies, participants were excluded from the study (*n* = 3). Cognitive functions were assessed separately the next day or the day after the next day. Blood samples were taken in the morning within 24 h after the initial clinical assessments.

### Statistical Analysis

Statistical analyses were performed with the Statistical Package for the Social Sciences (SPSS), version 21.0. Normality was checked by normal probability plots and by the Shapiro-Wilk and the Kolmogorov-Smirnov tests. Lipid and cognitive variables that showed skewed distributions were log-transformed, and all subsequent analyses were done with these transformed data. Between-group differences in demographic, lifestyle and clinical variables were analyzed by chi-square test and by ANOVA or non-parametric tests (Mann–Whitney U and Kruskal–Wallis). Differences between the study groups in the serum lipid and lipoprotein values, as well as cognitive performances, were tested first by one-way ANOVA. If the homogeneity assumption (tested by Levene’s statistic) was violated, Welch-probe was used for the group comparisons. Fisher’s LSD and Games-Howell tests were applied for *post hoc* pairwise comparisons. In the next step, between-group differences in the main variables were analyzed using ANCOVA with demographic and lifestyle variables as covariates and *post hoc* comparisons were done with Bonferroni correction.

After the group comparisons, hierarchical multiple linear regression analyses were run in the entire MDD group in order to explore whether the heterogeneity of lipid and lipoprotein levels were explained rather by the severity of depression or by the severity of ELS and whether there were associations between the revealed lipid alterations and the patients’ cognitive performances. Due to the relatively large number of background variables and the relatively small sample size, in the regression analyses, we selected the most relevant confounders using the forward procedure, and predictor variables of main interest were added to the models with the enter method. In the forward procedure, the predictor variables were sequentially included in the regression models depending on the strength of their correlation with the criterion variable (*P* to enter < 0.05). The entering procedure enters the predictor variables in the models irrespective of their significance with the criterion. In all analyses, *P*-values (two-tailed) below 0.05 were considered statistically significant. Effect sizes were measured by calculating Cohen’s d, η^2^ (for ANOVAs) as well as Cohen’s *f*^2^ (for multiple regression analyses).

## Results

### Demographic, Lifestyle, and Clinical Data

#### Two-Group Comparisons: Healthy Controls Versus the Entire MDD Group

There were no significant between-group differences in age (*F*_(__1__,__60__)_ = 0.024, *P* = 0.877), gender ratio (*X*^2^_(__1__)_ = 1.303, *P* = 0.254), BMI (*U* = 398.000*, Z* = *−*0.331*, P* = 0.740), and regular physical activity (*U* = 345.500*, Z* = *−*1.167*, P* = 0.243). The level of education was significantly lower (*U* = 190.000*, Z* = *−*3.612*, P* < 0.001), while the BDI score, as well as all CTQ scores (including total score and trauma type sub-scores) were significantly higher in MDD patients compared to the healthy subjects (BDI: Welch’s *F*_(__1__,__47__.__7__)_ = 146.324, *P* < 0.001); CTQ Total: *U* = 72.500*, Z* = *−*5.238*, P* < 0.001; CTQ PN: *U* = 110.000*, Z* = *−*4.787*, P* < 0.001; CTQ PA: *U* = 186,500*, Z* = *−*3.790*, P* < 0.001; CTQ EN: *F*_(__1__,__60__)_ = 26.407, *P* < 0.001; CTQ EA: *U* = 116.500*, Z* = *−*4,585*, P* < 0.001; CTQ SA: *U* = 230.000*, Z* = *−*3.506*, P* < 0.001) (for details see Table 1).

**TABLE 1:** Demographic, lifestyle and clinical characteristics of patients with MDD and HCs.

	**HC (*n* = 20)**	**Entire MDD (*n* = 42)**	**MDD Only (*n* = 21)**	**MDD + ELS (*n* = 21)**
**Demographic and lifestyle characteristics**				
Age (years)^a^	35.80 (8.53)	35.40 (9.73)	34.71 (8.17)	36.10 (11.24)
Gender (female/male)	13/7	33/9	17/4	16/5
Education (years)^b^	15.00 (5.00)	12.00 (1.00) ^∗∗∗^	12.00 (2.00) ^§^	12.00 (1.00) ^§§^
Physical exercise per week (hours)	1–2	2–4	1–2	2–4
Body mass index (kg/m^2^)^b^	23.39 (6.6)	23.11 (5.26)	23.12 (5.69)	23.11 (4.83)
**Early life stress**				
CTQ physical neglect^b^	5.00 (1.00)	9.00 (4.00) ^∗∗∗^	7.00 (4.50) ^§^	10.00 (5.50) ^§§§ ++^
CTQ physical abuse^b^	5.00 (0.00)	7.00 (5.25) ^∗∗∗^	5.00 (2.00)	10.00 (5.50) ^§§§^^+++^
CTQ emotional neglect^a^	9.10 (3.51)	13.40 (5.42) ^∗∗∗^	11.95 (3.56) ^§^	18.95 (3.47) ^§§§^^+++^
CTQ emotional abuse^b^	6.00 (2.75)	13.50 (10.25) ^∗∗∗^	9.00 (5.50)	19.00 (5.00) ^§§§^^+++^
CTQ sexual abuse^b^	5.00 (0.00)	5.00 (4.25) ^∗∗∗^	5.00 (0.50)	9.00 (7.50) ^§§§^^++^
CTQ total score^b^	29.00 (11.5)	54.50 (29.50) ^∗∗∗^	40.00 (17.00)	69.00 (18.50) ^§§§^^+++^
**Clinical data**				
BDI total score^a^	3.00 (2.13)	23.21 (10.38) ^∗∗∗^	20.05 (10.29) ^§§§^	26.38 (9.69) ^§§§^
Age at the onset of MDD^b^	-	25.5 (17–32.25)	28 (18–34)	20 (16–31.5)
Number of lifetime depressive episodes^b^	-	2 (1.75–3)	2 (1–3)	2 (2–3)
Double depression (*n*)	-	2	1	1
Chronic depression (*n*)	-	5	1	4
Recurrent depression (*n*)	-	31	13	18
**Lipid profile**				
Total cholesterol (mmol/L)^a^	4.85 (0.91)	5.10 (1.08)	5.04 (0.88)	5.16 (1.27)
Triglycerides (mmol/L)^b^	0.89 (0.93)	1.06 (0.78)	0.92 (0.39)	1.26 (1.05) ^+^
HDL cholesterol (mmol/L)^a^	1.65 (0.33)	1.58 (0.37)	1.70 (0.40)	1.45 (0.28) ^+^
LDL cholesterol (mmol/L)^a^	2.73 (0.77)	2.95 (0.79)	2.87 (0.61)	3.03 (0.95)
LDL-C/HDL-C^b^	1.75 (0.78)	1.81 (1.05)	1.70 (0.69)	2.00 (1.33)
TC/HDL-C^b^	3.04 (1.13)	3.22 (1.29)	3.04 (1.03)	3.27 (1.78) ^§^^+^

^*a*^Means and standard deviations are presented. ^*b*^Medians and inter-quartile ranges are presented. Two-group comparisons: overall MDD group compared to HC: ^*^P < 0.05, ^∗∗^*P* < 0.01, ^∗∗∗^*P* < 0.001. Three-group comparisons: MDD Only compared to MDD + ELS: ^+^*P* < 0.05, ^++^*P* < 0.01, ^+++^*P* < 0.001. MDD Only or MDD + ELS compared to HC: ^§^*P* < 0.05, ^§§^*P* < 0.01, ^§§§^*P* < 0.001. BDI, Beck Depression Inventory; CTQ, Childhood Trauma Questionnaire-Short Form; ELS, early life stress; HC, healthy controls; HDL-C, high-density lipoprotein cholesterol; LDL-C, low-density lipoprotein cholesterol; MDD, major depressive disorder; TC, total cholesterol.

#### Three-Group Comparisons: Healthy Controls Versus MDD Only Versus MDD + ELS

The three groups did not differ in age (*F*_(__2__,__59__)_ = 0.125, *P* = 0.883), gender ratio (*X*^2^_(__2__)_ = 1.428, *P* = 0.490), BMI (*X*^2^_(__2__)_ = 0.142, *P* = 0.931), and physical activity (*X*^2^_(__2__)_ = 3.083, *P* = 0.214), however, a significant difference could be observed between groups in years of education (*X*^2^_(__2__)_ = 14.079, *P* = 0.001). Pairwise comparisons showed that the level of education was significantly lower in the MDD Only and in the MDD + ELS groups compared to HC (*P* = 0.025, *P* = 0.001, respectively). As expected, CTQ total score, and the specific trauma sub-scores were significantly different between groups (CTQ Total: *X*^2^_(__2__)_ = 46.768, *P* < 0.001; CTQ PN: *X*^2^_(__2__)_ = 34.441, *P* < 0.001; CTQ PA: *X*^2^_(__2__)_ = 30.924, *P* < 0.001; CTQ EN: *F*_(__2__,__59__)_ = 43.020, *P* < 0.001; CTQ EA: *X*^2^_(__2__)_ = 37.808, *P* < 0.001; CTQ SA: *X*^2^_(__2__)_ = 23.897, *P* < 0.001) and *post hoc* comparisons revealed that the MDD + ELS group had significantly higher scores in all CTQ scales than the MDD Only group (CTQ Total: *P* < 001; CTQ PN: *P* = 0.002; CTQ PA: *P* < 0.001; CTQ EN: *P* < 0.001; CTQ EA: *P* < 0.001; CTQ SA: *P* = 0.002) (Table 1). The severity of ELS was significantly higher for physical and emotional neglect in the MDD Only group compared to HC (CTQ PN: *P* = 0.039; CTQ EN: *P* = 0.012), but there was no significant difference in CTQ total score, as well as in physical, emotional, and sexual abuse between these two groups (CTQ Total: *P* = 0.051; CTQ PA: *P* = 0.596; CTQ EA: *P* = 0.149; CTQ SA: *P* = 0.515). There was significant difference between the groups in BDI score (Welch’s *F*_(__2__.__29__,__0__)_ = 80.404, *P* < 0.001) and the pairwise comparisons demonstrated that both MDD subgroups had significantly higher BDI score than the HC group (*P* < 0.001), whereas depression severity was similar in the two MDD subgroups (*P* = 0.113) (Table 1).

### Lipid Profile

#### Two-Group Comparisons: Healthy Controls Versus the Entire MDD Group

**FIGURE 1:**
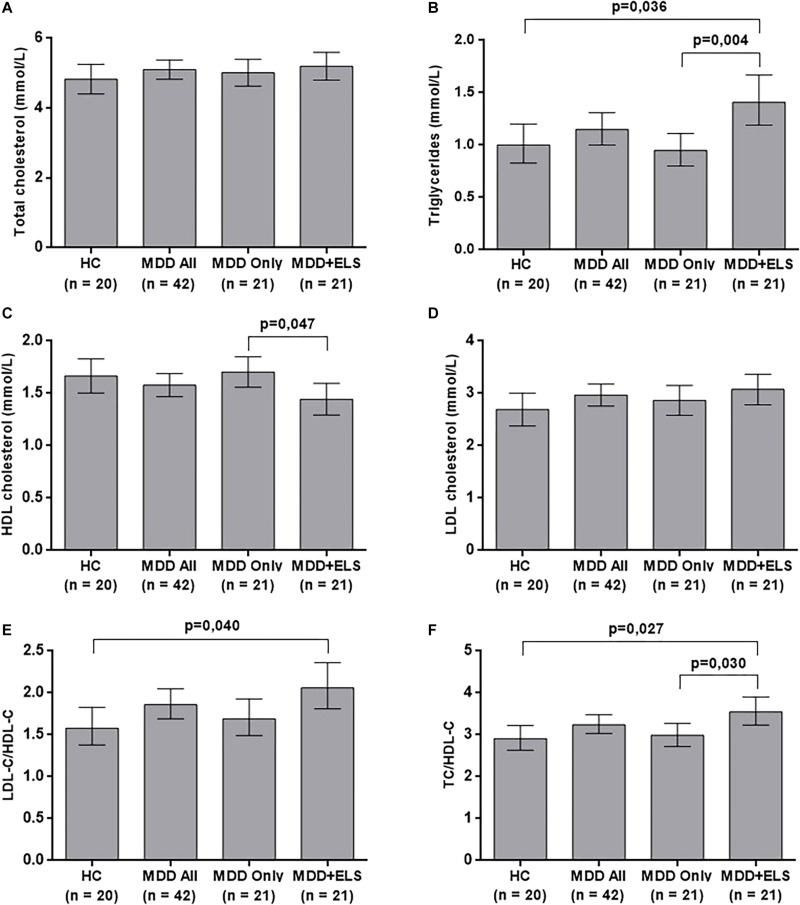
Serum lipid and lipoprotein levels in HCs and depressed patients after the adjustment for age, gender, education, physical exercise per week, and body mass index. **(A)** total cholesterol levels; **(B)** triglyceride levels; **(C)** high-density lipoprotein cholesterol levels; **(D)** low-density lipoprotein cholesterol levels; **(E)** LDL-C/HDL-C ratio; **(F)** TC/HDL-C ratio. The bars represent the means and upper and lower 95% confidence intervals of the examined lipid profile elements. The values of triglycerides, LDL-C/HDL-C and TC/HDL-C are results of back-transformation (antilog) because of the skewed distribution of the original data. The *P*-values of significant differences are shown. ELS, early life stress; HC, healthy controls; HDL-C, high-density lipoprotein cholesterol; LDL-C, low-density lipoprotein cholesterol; MDD, major depressive disorder; TC, total cholesterol.

No difference was found between the two groups by one-way ANOVA when we compared serum TC (*F*_(__1__,__60__)_ = 0.782*, P* = 0.380), TG (*F*_(__1__,__60__)_ = 0.426*, P* = 0.516), HDL-C (*F*_(__1__,__60__)_ = 0.609*, P* = 0.438), LDL-C (*F*_(__1__,__60__)_ = 1.062*, P* = 0.307), and the two atherogenic indices (LDL-C/HDL-C: *F*_(__1__,__60__)_ = 2.052*, P* = 0.157, TC/HDL-C: *F*_(__1__,__60__)_ = 2.036*, P* = 0.159) (Table 1). In order to control for the effects of demographic and lifestyle variables on lipid and lipoprotein levels, ANCOVAs were conducted with age, gender, level of education, physical exercise, and BMI as covariates, but again no significances were found (Figure 1 and Supplementary Table 1).

#### Three-Group Comparisons: Healthy Controls Versus MDD Only Versus MDD + ELS

Results of the ANOVA omnibus tests indicated significant between-group differences in TG (Welch’s *F*_(__2__,__35__.__4__)_ = 4.367, *P* = 0.020), HDL-C (*F*_(__2__,__59__)_ = 3.293, *P* = 0.044), and TC/HDL-C (*F*_(__2__,__59__)_ = 3.434, *P* = 0.039). *Post hoc* comparisons (Fisher’s LSD and Games-Howell tests) showed that the level of HDL-C was significantly lower (*P* = 0.018), while the level of TG (*P* = 0.015) and also the ratio TC/HDL-C (*p* = 0.034) were significantly higher in the MDD + ELS than in the MDD Only group. The ratio of TC/HDL-C of the MDD + ELS group was also significantly higher when compared to the HC group (*P* = 0.022) There were no significant differences between groups in TC (Welch’s *F*_(__2__,__38__.__7__)_ = 4.367, *P* = 0.645), LDL-C (*F*_(__2__,__59__)_ = 0.733, *P* = 0.485), and LDL-C/HDL-C (*F*_(__2__,__59__)_ = 2.562, *P* = 0.086) (Table 1). Cohen’s *d*-values for all significant group differences ranged from 0.68 to 0.78 indicating medium-to-large effect sizes.

After controlling for the effects of age, gender, level of education, physical exercise and BMI by ANCOVA, between-group differences remained significant in TG (*F*_(__2__,__54__)_ = 6.320, *P* = 0.003), HDL-C (*F*_(__2__,__54__)_ = 3.409, *P* = 0.040), and TC/HDL-C (*F*_(__2__,__54__)_ = 4.854, *P* = 0.012), and a new significant difference emerged in LDL-C/HDL-C (*F*_(__2__,__54__)_ = 3.794, *P* = 0.029). As it is shown in Figure 1, *post hoc* Bonferroni comparisons demonstrated that HDL-C was significantly lower in MDD patients with ELS than in MDD Only patients, as well as the TG and the TC/HDL-C index, were significantly higher in the MDD + ELS group compared both to the MDD Only and to the HC groups. Moreover, higher LDL-C/HDL-C ratio was revealed in MDD + ELS patients relative to the HC. There were no significant differences between groups by ANCOVA in TC (*F*_(__2__,__54__)_ = 0.742, *P* = 0.481) and LDL-C (*F*_(__2__,__54__)_ = 1.454, *P* = 0.243) (Figure 1). For the significant group comparisons, Cohen’s *d*-values ranged from 0.63 to 0.94 (medium-to-large effect sizes).

#### Multiple Linear Regression Analyses: The Effects of Depression Severity and ELS on Serum Lipid/Lipoprotein Levels

Next, we performed a series of hierarchical linear regression analyses in the entire MDD group to determine whether the heterogeneity of each lipid/lipoprotein level is explained by the severity of depression or by the amount of ELS after controlling for each other and for potentially confounding factors. Relevant confounders were selected from the demographic variables (age, gender, years of education) in Block 1, and from the lifestyle variables (BMI, physical exercise per week) in Block 2 using the forward variable selection procedure. Because we were interested in how the statistical effect of current depression severity on lipid/lipoprotein levels changes after including ELS in the models, next, in Block 3, depression severity (BDI score), and finally, in Block 4, the amount of ELS (CTQ total score) were added to the regression models using the enter method.

After running hierarchical regression analyses for each lipid and lipoprotein parameters as dependent variables, we found that in Block 3, BDI score predicted only HDL-C (*P* = 0.010) significantly. However, when the CTQ total score was also added in Block 4, the relationship between BDI and HDL-C lost its significance (*P* = 0.068) and no other significantly predictive relationship emerged between depression severity and any of the lipid profile elements (Table 2). However, in Block 4, the severity of ELS had a significant negative association with HDL-C level (*P* = 0.040) and a significant positive association with the serum level of TG (*P* = 0.014) and TC/HDL-C index (*P* = 0.043) (for details see Supplementary Table 2). Cohen’s *f*^2^-values for these significant associations ranged from 0.11 to 0.18 indicating moderate effect sizes.

**TABLE 2:** Hierarchical linear regression analyses predicting serum lipid and lipoprotein levels in the entire MDD group.

**Dependent variable**	**Blocks**	**Predictors**	***R*^2^**	**Δ*R*^2^**	**β**	**β’**
Total cholesterol	Block 1 (forward)	Age	0.157	0.157	0.396^∗∗^	0.381^∗∗^
	Block 2 (forward)	Body mass index	0.304	0.147	0.384^∗∗^	0.382^∗∗^
	Block 3 (enter)	**Depression severity**	0.311	0.007	*−*0.084	*−*0.080
	Block 4 (enter)	**Early life stress**	0.312	0.000		*−*0.013
Triglycerides	Block 1 (forward)	No variable associated				
	Block 2 (forward)	Physical exercise	0.108	0.108	*−*0.328^*^	*−*0.396^*^
	Block 3 (enter)	**Depression severity**	0.145	0.038	0.198	0.031
	Block 4 (enter)	**Early life stress**	0.273	0.128		0.400^*^
HDL cholesterol	Block 1 (forward)	No variable associated				
	Block 2 (forward)	No variable associated				
	Block 3 (enter)	**Depression severity**	0.156	0.156	*−*0.395^*^	*−*0.280^†^
	Block 4 (enter)	**Early life stress**	0.243	0.088		*−*0.317^*^
LDL cholesterol	Block 1 (forward)	Age	0.151	0.151	0.388^*^	0.392^∗∗^
	Block 2 (forward)	Physical exercise	0.318	0.167	*−*0.409^∗∗^	*−*0.453^∗∗^
	Block 3 (enter)	**Depression severity**	0.327	0.009	*−*0.096	*−*0.134
	Block 4 (enter)	**Early life stress**	0.333	0.007		0.092
LDL-C/HDL-C	Block 1 (forward)	No variable associated				
	Block 2 (forward)	Physical exercise	0.129	0.129	*−*0.359^*^	*−*0.391^*^
	Block 3 (enter)	**Depression severity**	0.177	0.048	0.223	0.104
	Block 4 (enter)	**Early life stress**	0.242	0.065		0.286^†^
TC/HDL-C	Block 1 (forward)	No variable associated				
	Block 2 (forward)	Physical exercise	0.126	0.126	*−*0.355^*^	*−*0.394^*^
	Block 3 (enter)	**Depression severity**	0.181	0.055	0.240	0.104
	Block 4 (enter)	**Early life stress**	0.266	0.085		0.326^*^

Input variables: Block 1: demographic variables (age, gender, and years of education); Block 2: lifestyle variables (body mass index and physical exercise per week); Block 3: depression severity (BDI score); Block 4: early life stress (CTQ total score). †*P* < 0.1,^*^P < 0.05, ^∗∗^*P* < 0.01. β, standardized beta coefficient at the current step; β’, the standardized beta coefficient in the final model; BDI, Beck Depression Inventory; CTQ, Childhood Trauma Questionnaire-Short Form; HDL-C, high-density lipoprotein cholesterol; LDL-C, low-density lipoprotein cholesterol; MDD; major depressive disorder; TC, total cholesterol. Bold values indicate the significant differences related to depression severity and ELS.

#### The Relationship Between the Different Subtypes of ELS and Serum Lipid/Lipoprotein Levels

Within the entire MDD group, additional series of hierarchical linear regressions were calculated to determine which subtypes of childhood adversities can significantly predict the parameters of the lipid profile as dependent variables after controlling for demographic variables (Block 1), lifestyle variables (Block 2), and depression severity (BDI score; Block 3) with the forward variable selection method. In Block 4, the CTQ subscores of the different trauma types, as predictor variables of main interest, were added to the models using the ‘enter’ procedure.

As it is shown in Table 3, we found significant negative associations between physical neglect and abuse and between HDL-C. We also found significant positive associations between physical and emotional neglect and abuse, and the levels of TG. Moreover, significant positive associations were found between physical and emotional neglect and the indices of LDL-C/HDL-C and TC/HDL-C. Sexual abuse had no statistically significant relationship between any of the lipid parameters (Table 3; for details see Supplementary Table 3).

**TABLE 3:** Linear regression analyses with serum lipid and lipoprotein levels as dependent variables, and with trauma types (CTQ sub-scores) as predictors in the entire MDD group.

	**Total cholesterol^a^**		**Triglycerides^b^**		**HDL cholesterol^c^**		**LDL cholesterol^d^**		**LDL-C/HDL-C^b^**		**TC/HDL-C^b^**	
						
	**β**	***p***	**β**	***p***	**β**	***p***	**β**	***p***	**β**	***p***	**β**	***p***
CTQ physical neglect	0.150	0.277	**0.351**	**0.017**	*−***0.306**	**0.034**	0.179	0.182	**0.392**	**0.006**	**0.403**	**0.005**
CTQ physical abuse	*−*0.166	0.231	**0.320**	**0.031**	*−***0.304**	**0.037**	*−*0.089	0.516	0.200	0.180	0.197	0.187
CTQ emotional neglect	0.176	0.198	**0.381**	**0.010**	*−*0.200	0.188	0.194	0.148	**0.358**	**0.014**	**0.419**	**0.004**
CTQ emotional abuse	*−*0.087	0.529	**0.308**	**0.041**	*−*0.291	0.054	0.008	0.956	0.223	0.138	0.248	0.099
CTQ sexual abuse	*−*0.240	0.083	0.114	0.463	0.050	0.764	*−*0.195	0.169	0.003	0.984	0.045	0.772

*^*a*^Adjusted for age and body mass index. ^*b*^Adjusted for physical exercise per week. ^*c*^Adjusted for depression severity. ^*d*^Adjusted for age and physical exercise per week. CTQ, Childhood Trauma Questionnaire Short Form; HDL-C, high-density lipoprotein cholesterol; LDL-C, low-density lipoprotein cholesterol; MDD, major depressive disorder; TC, total cholesterol. Bold values indicate the significant differences.*

### Neurocognitive Tests

#### Two-Group Comparisons: Healthy Controls Versus the Entire MDD Group

One-way ANOVA revealed significant group differences when we compared omission errors of the Conner’s Continuous Performance Test (Welch’s *F*_(__1__,__58__.__5__)_ = 7.464, *P* = 0.008, Cohen’s *d* = 0.75). Similarly, one-way ANOVA revealed significant group differences when we compared perseverative errors of the WCST (Welch’s *F*_(__1__,__50__.__8__)_ = 5.463, *P* = 0.023, Cohen’s *d* = 0.63) (for details see Supplementary Table 4). After controlling by ANCOVA for age, gender, and level of education, however, these differences lost their significance (Supplementary Table 5).

#### Three-Group Comparisons: Healthy Controls Versus MDD Only Versus MDD + ELS

The ANOVA omnibus tests revealed that omission errors of the Conner’s Continuous Performance Test were significantly different in the three groups (Welch’s *F*_(__2__,__36__.__6__)_ = 3.780, *P* = 0.032, Cohen’s *d* = 0.64). Further comparison with the Games-Howell *post hoc* test revealed that the CPT omission errors were significantly higher in the MDD + ELS group than in the HC (*P* = 0.045, Cohen’s *d* = 0.65) (for details see Supplementary Table 4). However, after controlling for the effects of demographic variables, no significant between-group differences were found in the neurocognitive variables (Supplementary Table 5).

#### The Effect of Serum Lipid/Lipoprotein Levels on Neurocognitive Performances in MDD

Finally, hierarchical multiple linear regressions were calculated to predict parameters of neurocognitive tests based on lipid parameters after controlling for demographic variables (Block 1), lifestyle variables (Block 2), severity of depression (i.e., BDI score; Block 3), and severity of ELS (i.e., CTQ total score; Block 4) that were included in the regression models with the forward procedure. The lipid profile elements, as predictor variables of main interest, were added to the models using the enter method in Block 5.

Depression severity predicted commission errors in the Conner’s Continuous Performance Test (β = 0.289, *P* = 0.024) and detectability (β = *−*0.304, *P* = 0.020), as well as conceptual level responses in the WCST (β = *−*0,416, *P* = 0.006) in Block 3 (for details see Supplementary Tables 6, 7). For these significant associations between depression severity and cognitive performances, Cohen’s *f*^2^-values ranged from 0.15 to 0.20 suggesting moderate effect sizes. However, we could not find any association between the amount of ELS and any of the neurocognitive test results in Block 4. No relationship was found between lipid parameters and any of the Conner’s Continuous Performance Test results (Supplementary Table 6). However, we could detect significant negative associations between the lipid profiles and between specific domains of the WCST. There were significant negative associations between HDL-C and WCST perseverative errors, between LDL-C/HDL-C ratio and WCST total correct responses, and also between the indices LDL-C/HDL-C and TC/HDL-C, and WCST conceptual level responses (Table 4, for details, see Supplementary Table 7). Cohen’s *f*^2^-values for these results ranged from 0.10 to 0.16 suggesting moderate effect sizes.

**TABLE 4:** Linear regression analyses of serum lipid and lipoprotein levels as predictors of executive functioning (WCST scores) in the entire MDD group.

	**WCST total correct responses**		**WCST perseverative errors^a^**		**WCST non-perseverative errors^b^**		**WCST conceptual level responses^c^**	
				
	**β**	***p***	**β**	***p***	**β**	***p***	**β**	***p***
Total cholesterol	*−*0.130	0.411	*−*0.088	0.532	*−*0.038	0.794	*−*0.153	0.292
Triglycerides	*−*0.235	0.134	0.219	0.100	0.062	0.673	*−*0.266	0.074
HDL cholesterol	0.167	0.290	*−***0.283**	**0.027**	*−*0.167	0.255	0.192	0.224
LDL cholesterol	*−*0.236	0.133	*−*0.068	0.629	*−*0.036	0.806	*−*0.241	0.093
LDL-C/HDL-C	*−***0.306**	**0.048**	0.151	0.251	0.105	0.471	*−***0.340**	**0.022**
TC/HDL-C	*−*0.252	0.108	0.193	0.139	0.146	0.318	*−***0.309**	**0.039**

*^*a*^Adjusted for age and education. ^*b*^Adjusted for education. ^*c*^Adjusted for depression severity. HDL-C, high-density lipoprotein cholesterol; LDL-C, low-density lipoprotein cholesterol; MDD; major depressive disorder; TC, total cholesterol; WCST, Wisconsin Card Sorting Test. Bold values indicate the significant differences.*

## Discussion

The principal aim of the present study was to examine the impact of childhood adversities on serum lipid profiles in depressed patients. In our statistical analysis, we asked the question of whether depression severity or the severity of ACEs have a stronger influence determining serum lipid levels. Overall, ELS was a stronger predictor of serum lipid profiles than depression severity. Furthermore, we found that depressed patients with ELS had significantly higher serum triglyceride and lower HDL-cholesterol concentrations compared to MDD patients without ELS. The atherogenic indices, LDL-C/HDL-C, and TC/HDL-C were also higher in patients with ELS. We also found significant associations between the different trauma types and lipid profiles. Both physical and emotional neglect and abuse had a significant positive association with serum triglyceride levels, while physical neglect and abuse had a significant negative association with HDL-cholesterol. Finally, we could detect significant associations between depression severity and specific domains of the cognitive tests as well as between lipid profiles and certain results of the WCST. But in our study, ELS had no influence on the cognitive performance of the subjects.

A vast number of studies report that early life adversity may increase cardiovascular risk factors and the occurrence of CVD (Batten et al., 2004; Dong et al., 2004; Goodwin and Stein, 2004; Danese et al., 2009; Fuller-Thomson et al., 2010, 2012; Korkeila et al., 2010; Stein et al., 2010; Scott et al., 2011; Rich-Edwards et al., 2012; Basu et al., 2017; Murphy et al., 2017; Reid et al., 2018; Doom et al., 2019; Obi et al., 2019). These studies document that childhood adversities are associated with hypertension (Danese et al., 2009; Stein et al., 2010; Reid et al., 2018; Doom et al., 2019), higher BMI (Doom et al., 2019), ischemic heart disease (Dong et al., 2004) and myocardial infarction (Fuller-Thomson et al., 2012). Adverse childhood experience may alter serum lipid/lipoprotein profiles as adults with ELS may have elevated serum TG, LDL-cholesterol and TC as well as low HDL-cholesterol (Danese et al., 2009; Spann et al., 2014; Reid et al., 2018; Doom et al., 2019). Furthermore, a recent study reported that the different trauma types can be associated with specific changes in serum levels, i.e., physical and sexual abuse were associated with high LDL-C and low HDL-C, and childhood neglect with raised TG and low HDL-C (Li et al., 2019). The exact physiological pathways connecting ELS with CVD risk factors and CVD are yet unknown. Recently, a hypothesis has been put forward that experiencing social threat and adversity up-regulates pro-inflammatory cytokines which in turn may elicit depressive symptoms as well as metabolic syndrome and CVD (Slavich and Irwin, 2014).

Large body of evidence indicate that there is a strong association between MDD and CVD (Musselman et al., 1998; Penninx et al., 2001; Carney et al., 2002; Barth et al., 2004; Whooley, 2006; Van der Kooy et al., 2007; Goldstein et al., 2015). While the exact relationship between these two disorders remains obscure there is evidence that the presence of depressive symptoms can increase the risk of CVD (Joynt et al., 2003; Almeida et al., 2007; Vancampfort et al., 2014; Pérez-Piñar et al., 2016). Among the various CVD risk factors dyslipidemia has also been associated with depressed mood (Huang et al., 2003; Papakostas et al., 2004; van Reedt Dortland et al., 2010, 2013; Chien et al., 2013). However, the studies investigating serum lipid concentrations in MDD yielded inconsistent results. There are reports on higher (Ledochowski et al., 2003; Nakao and Yano, 2004; Moreira et al., 2017) as well as lower serum TC levels (Olusi and Fido, 1996; Maes et al., 1997; Ong et al., 2016) compared to controls, while others found no difference (Lehto et al., 2008; van Reedt Dortland et al., 2010; Enko et al., 2018). Other studies found that TG levels are increased in patients with MDD and that TG levels show a positive relationship with depression severity (Sevincok et al., 2001; Huang and Chen, 2004; Liu et al., 2016).

So far only a handful of studies examined the influence of childhood adversity on lipid profiles in depressed patients. McIntyre et al. (2012) examined a clinical population with unipolar depression and found a significantly lower level of HDL-C in patients who experienced traumatic life events during their childhood compared to those without childhood adversities. However, there was no statistically significant difference in the overall rate of dyslipidemia and/or metabolic syndrome between subjects with and without childhood adversity. Wingenfeld et al. (2017) conducted a women-only study in a physically healthy clinical sample and detected no difference in TG, cholesterol, HDL-C, LDL-C and other metabolic risk markers between MDD patients with and without sexual or physical abuse. More recently Deschênes et al. (2018) reported that ACEs are indirectly associated with diabetes via depressive symptoms and cardio-metabolic dysregulations. The most recent study found decreased TC levels in adult outpatients with MDD with a childhood history of physical violence (Kraav et al., 2019). The same study found no differences in serum levels of HDL-C and LDL-C between the groups (Kraav et al., 2019). In our present study, we could detect higher serum triglyceride and lower HDL-cholesterol levels in MDD patients who experienced childhood adversity compared to MDD patients without ELS. Furthermore, we also found that the severity of ELS had a negative association with HDL-cholesterol levels and positive associations with the serum level of TG and TC/HDL-C index. Thus, our present data support the notion that childhood adversity may influence serum lipid levels also in depressed individuals and that MDD patients with a history of childhood adversity may represent a specific sub-group within MDD. We could also detect significant associations between the different trauma types and lipid profiles. Physical neglect and abuse had a significant negative association with HDL-cholesterol while physical and emotional neglect and abuse had a significant positive association with serum triglyceride levels. Our findings are in harmony with the recent report of Li et al. (2019), which reported that physical abuse was associated with low HDL-C, while neglect was associated with raised TG and lower HDL-C. In our present study, we could not detect any association between sexual abuse and serum lipid/lipoprotein levels. Others found that sexual abuse was associated with high LDL-C and low HDL-C (Li et al., 2019). There is, in fact, ample evidence in the literature that childhood sexual abuse can increase the incidence of CVD: a US study involving 5 900 subjects reported that childhood sexual abuse was associated with increased risk of cardiac disease (Goodwin and Stein, 2004). Another US survey involving 12 900 individuals found that specifically in men childhood sexual abuse was associated with heart attack (Fuller-Thomson et al., 2012). One should add that there are negative findings as well, e.g., a recent retrospective study involving 3 600 individuals could not reveal any consistent association between the specific type of early psychosocial adversity and CVD risk factors (Anderson et al., 2018). This study examined associations of specific types of psychosocial adversities, such as lack of maternal care, maternal overprotection, parental mental illness, household dysfunction, sexual abuse, physical and emotional abuse, and neglect in childhood with CVD risk factors including BMI, TG, low and high density lipoprotein cholesterol (Anderson et al., 2018).

A vast body of work has linked early life adversity to various types of cognitive deficits later in life (see e.g., Evans and Schamberg, 2009; Mueller et al., 2010; Pechtel and Pizzagalli, 2011; Gould et al., 2012; Chen and Baram, 2016). Cognitive impairments are also frequently present in depressed individuals (Porter et al., 2003; Marazziti et al., 2010; Ahern and Semkovska, 2017). A meta-analysis found significant cognitive deficits in executive function, memory and attention in depressed patients relative to controls (Rock et al., 2014), yet another one revealed significant correlations between depression severity and specific domains of episodic memory, executive function, and processing speed (McDermott and Ebmeier, 2009). In our present study, we could also detect significant associations between depression severity and specific domains of attention (examined with the CPT-II) and executive functions (investigated with the WCST). However, we could not find any association between ELS and cognitive performance using these two tests.

Numerous clinical and preclinical data suggest that dyslipidemia can be linked to cognitive deficits and decline (Yaffe et al., 2002; Farr et al., 2008; Gendle et al., 2008; Morley and Banks, 2010; Reynolds et al., 2010) though this issue is not without controversies (see e.g., Panza et al., 2006; Anstey et al., 2008). For example, there are reports that high TG are associated with poor memory and general cognitive decline (de Frias et al., 2007; Morley and Banks, 2010), and that high triglyceride levels inversely correlate with executive function in non-demented elderly adults (Parthasarathy et al., 2017). Furthermore, a recent study documented elevated triglyceride levels in patients with MDD, which was associated with cognitive impairments (Shao et al., 2017). In our study, we found negative associations between lipid profiles (HDL-C and LDL-C/HDL-C, TC/HDL-C ratios) and specific domains of the WCST measuring executive functions. Low levels of HDL cholesterol have been associated with poor memory (Singh-Manoux et al., 2008; Feinkohl et al., 2019), impaired executive functions (Sun et al., 2019) and cognitive decline (van Exel et al., 2002), as well as with lower gray matter volumes (Ward et al., 2010). It should be added here that higher levels of HDL-C have been associated with a decreased risk of Alzheimer’s disease (Reitz et al., 2010) and that low HDL-C levels can result in cerebral amyloidosis (Reed et al., 2014).

The low sample size is a major limitation of this study. A further important limitation is that we used a retrospective self-report to asses ELS. Ideally, the long-term effects of childhood adversities should be studied in prospective longitudinal studies and using qualitative or mixed methods can also add further valuable information when studying the impact of experienced traumas (see e.g., Boeije et al., 2013; Esposito et al., 2019), especially because self-reports can be biased. For example, social desirability can be an important potential bias when reporting past traumatic events especially in health-related research (see e.g., Adams et al., 2005; van de Mortel, 2008; Caputo, 2017 on this topic). Another limitation of our study design is that it does not allow to derive causal relations, but only associations. To compensate these limitations we did our best to carefully select the participants and match them in age, gender, lifestyle habits, and clinical data. Notably, only a few studies (Ding et al., 2014; Wingenfeld et al., 2017) included a control group in their studies, besides the MDD patients with or without ELS. We also carefully analyzed the influence of the various ACE subtypes. Finally, we also assessed the cognitive performance of our subjects and none of the earlier studies did such measurements.

Our present findings, together with the results available in the literature, have important clinical implications regarding the psychological interventions in case of depressed patients with ELS. Several studies demonstrated that depressed adults who experienced ELS react less well to conventional treatments than those who were not exposed to stressful life events during childhood (reviewed by Targum and Nemeroff, 2019). There is some evidence that MDD with ELS reacts much better to cognitive behavioral therapy (Nemeroff et al., 2003; Niciu et al., 2015) or interpersonal therapy (Zobel et al., 2011) than to pharmacotherapy. Psychodynamic therapies, as well as mentalizing-based therapy, can also be beneficial for MDD patients with ELS (Alessi and Kahn, 2017; Luyten and Fonagy, 2018). Our data emphasize the importance of the screening for ELS in the clinical MDD population. In case of early emotional abuse and emotional and physical neglect, we should consider psychotherapeutic interventions. Relying on relational cooperation, psychodynamic psychotherapy interventions can be especially helpful for patients with ELS, as they can establish an atmosphere of acceptance and safety, factors that are extremely relevant in early traumatized individuals. The holding environment and containment, created in this way, can provide a basis for the therapy of mood symptoms, and it may also reduce the risks for somatic complications. In addition, mentalizing based therapy can support early traumatized patients with an insecure attachment to regulate their negative affective states, and reduce stress, instead of using unhealthy methods to cope with stressful situations.

In summary, our present data provide further evidence that childhood adversity may increase the risk of CVD. We found that depressed patients with ELS had higher serum triglyceride and lower HDL-cholesterol concentrations compared to patients without ELS. The severity of childhood adversity and the different trauma types showed specific associations with the lipid profiles, but we could not find any association between the severity of ELS and cognitive performance. Further research is needed to clarify the exact intermediary factors in order to gain a better understanding on the physiological mechanisms linking childhood adversities to cardio-metabolic disease, including the exploration of the difference as well as common pathways for specific maltreatment. Importantly, these issues should preferably be investigated in longitudinal studies as the retrospective self-reported measures might be biased. Finally, our present findings highlight the importance of controlling ELS, especially when a psychiatric sample is studied and treated.

## Data Availability

All datasets generated for this study are included in the manuscript and/or the Supplementary Files.

## Ethics Statement

The local Research Ethics Committee of the University of Pécs approved the study design and protocol (Ethical Approval Nr.: 2015/5626) and all participants provided written informed consent.

## Author Contributions

ÁP, BC, and MS conceived the study, designed the experiments, and wrote the manuscript. NN carried out the psychological and neurocognitive tests with the subjects, analyzed the data, prepared the tables and the figure, and wrote the manuscript. RH helped with the statistical analysis. MS selected the patients and made the diagnosis. TT and AM provided supervision and had helpful comments on the interpretation of the data. All authors contributed to the writing of the manuscript and/or revising it critically for important intellectual content, approved the final version to be published, and agreed to be accountable for all aspects of the work in ensuring that questions related to the accuracy or integrity of any part of the work are appropriately investigated and resolved.

## Conflict of Interest Statement

The authors declare that the research was conducted in the absence of any commercial or financial relationships that could be construed as a potential conflict of interest.

## Abbreviations

ACEs: adverse childhood experiences
BDI: Beck Depression Inventory
BMI: body mass index
CPT-II: Conner’s Continuous Performance Test-II
CTQ EA: Childhood Trauma Questionnaire Emotional Abuse
CTQ EN: Childhood Trauma Questionnaire Emotional Neglect
CTQ PA: Childhood Trauma Questionnaire Physical Abuse
CTQ PN: Childhood Trauma Questionnaire Physical Neglect
CTQ SA: Childhood Trauma Questionnaire Sexual Abuse
CTQ: Childhood Trauma Questionnaire
CVD: cardiovascular disease
ELS: early life stress
HC: healthy control
HDL-C: high-density lipoprotein cholesterol
LDL-C: low-density lipoprotein cholesterol
MDD: major depressive disorder
TC: total cholesterol
TG: triglycerides
WCST: Wisconsin Card Sorting Test.
