# Supplementary material for: Examining the Influence of Early Life Stress on Serum Lipid Profiles and Cognitive Functioning in Depressed Patients
Source: Front Psychol. 2019 Aug 6;10:1798. doi: 10.3389/fpsyg.2019.01798 (PMC6691174; doi:10.3389/fpsyg.2019.01798)
Supplement: Supplementary file 1 [file Table_1.DOCX]

Supplementary Material

Examining the influence of early life stress on serum lipid profiles and cognitive functioning in depressed patients

Ágnes Péterfalvi § ^1,2^, Nándor Németh § ^1^, Róbert Herczeg ^3^, Tamás Tényi ^4^, Attila Miseta ^2^, Boldizsár Czéh ^1,2^ and Maria Simon ^1,4^*

§ These authors contributed equally

*** Correspondence:** Maria Simon, E-mail: [simon.maria@pte.hu](mailto:simon.maria@pte.hu)

## Supplementary Table 1.

## Results of the one-way ANCOVA comparisons between healthy controls and the entire MDD group in serum lipid and lipoprotein levels

| Total cholesterol | *F_(1,55)_=*1.021*, P=*0.317 |
| --- | --- |
| Triglycerides | *F_(1,55)_=*0.943*, P=*0.336 |
| HDL cholesterol | *F_(1,55)_=*0.546*, P=*0.463 |
| LDL cholesterol | *F_(1,55)_=*1.837*, P=*0.181 |
| LDL-C/HDL-C | *F_(1,55)_=*2.781*, P=*0.101 |
| TC/HDL-C | *F_(1,55)_=*2.298*, P=*0.135 |

## Supplementary Table 2.

## Hierarchical linear regression analyses of depression severity (BDI score) and early life stress (CTQ total score) as predictors of serum lipid and lipoprotein levels in the entire MDD group

| **Model Summary** | | | | | | | | | |
| --- | --- | --- | --- | --- | --- | --- | --- | --- | --- |
| Model | R | R Square | Adjusted R Square | Std. Error of the Estimate | Change Statistics | | | | |
|  |  |  |  |  | R Square Change | F Change | df1 | df2 | Sig. F Change |
| 1 | ,396^a^ | ,157 | ,136 | 1,00399 | ,157 | 7,451 | 1 | 40 | ,009 |
| 2 | ,552^b^ | ,304 | ,269 | ,92373 | ,147 | 8,253 | 1 | 39 | ,007 |
| 3 | ,558^c^ | ,311 | ,257 | ,93101 | ,007 | ,392 | 1 | 38 | ,535 |
| 4 | ,558^d^ | ,312 | ,237 | ,94342 | ,000 | ,007 | 1 | 37 | ,933 |
| a. Predictors: (Constant), Age | | | | | | | | | |
| b. Predictors: (Constant), Age, Body_mass_index | | | | | | | | | |
| c. Predictors: (Constant), Age, Body_mass_index, Depression_severity | | | | | | | | | |
| d. Predictors: (Constant), Age, Body_mass_index, Depression_severity, Early_life_stress | | | | | | | | | |

| **Coefficients^a^** | | | | | | | | |
| --- | --- | --- | --- | --- | --- | --- | --- | --- |
| Model | | Unstandardized Coefficients | | Standardized Coefficients | t | Sig. | 95,0% Confidence Interval for B | |
|  |  | B | Std. Error | Beta |  |  | Lower Bound | Upper Bound |
| 1 | (Constant) | 3,540 | ,591 |  | 5,988 | ,000 | 2,345 | 4,735 |
|  | Age | ,044 | ,016 | ,396 | 2,730 | ,009 | ,011 | ,077 |
| 2 | (Constant) | 1,635 | ,857 |  | 1,907 | ,064 | -,099 | 3,370 |
|  | Age | ,042 | ,015 | ,375 | 2,801 | ,008 | ,012 | ,072 |
|  | Body_mass_index | ,083 | ,029 | ,384 | 2,873 | ,007 | ,025 | ,141 |
| 3 | (Constant) | 1,819 | ,913 |  | 1,993 | ,053 | -,028 | 3,667 |
|  | Age | ,042 | ,015 | ,379 | 2,809 | ,008 | ,012 | ,072 |
|  | Body_mass_index | ,083 | ,029 | ,385 | 2,853 | ,007 | ,024 | ,142 |
|  | Depression_severity | -,009 | ,014 | -,084 | -,626 | ,535 | -,037 | ,020 |
| 4 | (Constant) | 1,861 | 1,047 |  | 1,778 | ,084 | -,260 | 3,982 |
|  | Age | ,042 | ,015 | ,381 | 2,759 | ,009 | ,011 | ,073 |
|  | Body_mass_index | ,082 | ,030 | ,382 | 2,739 | ,009 | ,021 | ,143 |
|  | Depression_severity | -,008 | ,015 | -,080 | -,544 | ,589 | -,039 | ,023 |
|  | Early_life_stress | -,001 | ,010 | -,013 | -,085 | ,933 | -,021 | ,019 |
| a. Dependent Variable: Total_cholesterol | | | | | | | | |

| **Model Summary** | | | | | | | | | |
| --- | --- | --- | --- | --- | --- | --- | --- | --- | --- |
| Model | R | R Square | Adjusted R Square | Std. Error of the Estimate | Change Statistics | | | | |
|  |  |  |  |  | R Square Change | F Change | df1 | df2 | Sig. F Change |
| 1 | ,328^a^ | ,108 | ,085 | ,18104 | ,108 | 4,830 | 1 | 40 | ,034 |
| 2 | ,381^b^ | ,145 | ,102 | ,17944 | ,038 | 1,716 | 1 | 39 | ,198 |
| 3 | ,523^c^ | ,273 | ,216 | ,16764 | ,128 | 6,683 | 1 | 38 | ,014 |
| a. Predictors: (Constant), Physical_exercise | | | | | | | | | |
| b. Predictors: (Constant), Physical_exercise, Depression_severity | | | | | | | | | |
| c. Predictors: (Constant), Physical_exercise, Depression_severity, Early_life_stress | | | | | | | | | |

| **Coefficients^a^** | | | | | | | | |
| --- | --- | --- | --- | --- | --- | --- | --- | --- |
| Model | | Unstandardized Coefficients | | Standardized Coefficients | t | Sig. | 95,0% Confidence Interval for B | |
|  |  | B | Std. Error | Beta |  |  | Lower Bound | Upper Bound |
| 1 | (Constant) | ,228 | ,084 |  | 2,712 | ,010 | ,058 | ,398 |
|  | Physical_exercise | -,046 | ,021 | -,328 | -2,198 | ,034 | -,089 | -,004 |
| 2 | (Constant) | ,123 | ,116 |  | 1,060 | ,296 | -,112 | ,357 |
|  | Physical_exercise | -,041 | ,021 | -,288 | -1,902 | ,065 | -,084 | ,003 |
|  | Depression_severity | ,004 | ,003 | ,198 | 1,310 | ,198 | -,002 | ,009 |
| 3 | (Constant) | -,003 | ,119 |  | -,023 | ,982 | -,243 | ,238 |
|  | Physical_exercise | -,056 | ,021 | -,396 | -2,688 | ,011 | -,098 | -,014 |
|  | Depression_severity | ,001 | ,003 | ,031 | ,202 | ,841 | -,005 | ,006 |
|  | Early_life_stress | ,005 | ,002 | ,400 | 2,585 | ,014 | ,001 | ,008 |
| a. Dependent Variable: Triglycerides | | | | | | | | |

| **Model Summary** | | | | | | | | | |
| --- | --- | --- | --- | --- | --- | --- | --- | --- | --- |
| Model | R | R Square | Adjusted R Square | Std. Error of the Estimate | Change Statistics | | | | |
|  |  |  |  |  | R Square Change | F Change | df1 | df2 | Sig. F Change |
| 1 | ,395^a^ | ,156 | ,135 | ,34075 | ,156 | 7,388 | 1 | 40 | ,010 |
| 2 | ,493^b^ | ,243 | ,205 | ,32671 | ,088 | 4,512 | 1 | 39 | ,040 |
| a. Predictors: (Constant), Depression_severity | | | | | | | | | |
| b. Predictors: (Constant), Depression_severity, Early_life_stress | | | | | | | | | |

| **Coefficients^a^** | | | | | | | | |
| --- | --- | --- | --- | --- | --- | --- | --- | --- |
| Model | | Unstandardized Coefficients | | Standardized Coefficients | t | Sig. | 95,0% Confidence Interval for B | |
|  |  | B | Std. Error | Beta |  |  | Lower Bound | Upper Bound |
| 1 | (Constant) | 1,902 | ,130 |  | 14,624 | ,000 | 1,639 | 2,165 |
|  | Depression_severity | -,014 | ,005 | -,395 | -2,718 | ,010 | -,024 | -,004 |
| 2 | (Constant) | 2,198 | ,187 |  | 11,760 | ,000 | 1,820 | 2,576 |
|  | Depression_severity | -,010 | ,005 | -,280 | -1,876 | ,068 | -,021 | ,001 |
|  | Early_life_stress | -,007 | ,003 | -,317 | -2,124 | ,040 | -,014 | ,000 |
| a. Dependent Variable: HDL_cholesterol | | | | | | | | |

| **Model Summary** | | | | | | | | | |
| --- | --- | --- | --- | --- | --- | --- | --- | --- | --- |
| Model | R | R Square | Adjusted R Square | Std. Error of the Estimate | Change Statistics | | | | |
|  |  |  |  |  | R Square Change | F Change | df1 | df2 | Sig. F Change |
| 1 | ,388^a^ | ,151 | ,130 | ,74086 | ,151 | 7,108 | 1 | 40 | ,011 |
| 2 | ,564^b^ | ,318 | ,283 | ,67256 | ,167 | 9,537 | 1 | 39 | ,004 |
| 3 | ,571^c^ | ,327 | ,273 | ,67692 | ,009 | ,500 | 1 | 38 | ,484 |
| 4 | ,577^d^ | ,333 | ,261 | ,68259 | ,007 | ,371 | 1 | 37 | ,546 |
| a. Predictors: (Constant), Age | | | | | | | | | |
| b. Predictors: (Constant), Age, Physical_exercise | | | | | | | | | |
| c. Predictors: (Constant), Age, Physical_exercise, Depression_severity | | | | | | | | | |
| d. Predictors: (Constant), Age, Physical_exercise, Depression_severity, Early_life_stress | | | | | | | | | |

| **Coefficients^a^** | | | | | | | | |
| --- | --- | --- | --- | --- | --- | --- | --- | --- |
| Model | | Unstandardized Coefficients | | Standardized Coefficients | t | Sig. | 95,0% Confidence Interval for B | |
|  |  | B | Std. Error | Beta |  |  | Lower Bound | Upper Bound |
| 1 | (Constant) | 1,830 | ,436 |  | 4,194 | ,000 | ,948 | 2,711 |
|  | Age | ,032 | ,012 | ,388 | 2,666 | ,011 | ,008 | ,056 |
| 2 | (Constant) | 2,718 | ,490 |  | 5,553 | ,000 | 1,728 | 3,708 |
|  | Age | ,032 | ,011 | ,396 | 2,994 | ,005 | ,010 | ,054 |
|  | Physical_exercise | -,242 | ,078 | -,409 | -3,088 | ,004 | -,401 | -,084 |
| 3 | (Constant) | 2,917 | ,567 |  | 5,142 | ,000 | 1,768 | 4,065 |
|  | Age | ,033 | ,011 | ,402 | 3,012 | ,005 | ,011 | ,055 |
|  | Physical_exercise | -,254 | ,081 | -,428 | -3,148 | ,003 | -,417 | -,091 |
|  | Depression_severity | -,007 | ,010 | -,096 | -,707 | ,484 | -,028 | ,014 |
| 4 | (Constant) | 2,822 | ,593 |  | 4,758 | ,000 | 1,620 | 4,023 |
|  | Age | ,032 | ,011 | ,392 | 2,896 | ,006 | ,010 | ,054 |
|  | Physical_exercise | -,269 | ,085 | -,453 | -3,167 | ,003 | -,440 | -,097 |
|  | Depression_severity | -,010 | ,012 | -,134 | -,890 | ,379 | -,034 | ,013 |
|  | Early_life_stress | ,004 | ,007 | ,092 | ,609 | ,546 | -,010 | ,019 |
| a. Dependent Variable: LDL_cholesterol | | | | | | | | |

| **Model Summary** | | | | | | | | | |
| --- | --- | --- | --- | --- | --- | --- | --- | --- | --- |
| Model | R | R Square | Adjusted R Square | Std. Error of the Estimate | Change Statistics | | | | |
|  |  |  |  |  | R Square Change | F Change | df1 | df2 | Sig. F Change |
| 1 | ,359^a^ | ,129 | ,107 | ,13707 | ,129 | 5,923 | 1 | 40 | ,020 |
| 2 | ,420^b^ | ,177 | ,134 | ,13498 | ,048 | 2,251 | 1 | 39 | ,142 |
| 3 | ,492^c^ | ,242 | ,182 | ,13121 | ,065 | 3,271 | 1 | 38 | ,078 |
| a. Predictors: (Constant), Physical_exercise | | | | | | | | | |
| b. Predictors: (Constant), Physical_exercise, Depression_severity | | | | | | | | | |
| c. Predictors: (Constant), Physical_exercise, Depression_severity, Early_life_stress | | | | | | | | | |

| **Coefficients^a^** | | | | | | | | |
| --- | --- | --- | --- | --- | --- | --- | --- | --- |
| Model | | Unstandardized Coefficients | | Standardized Coefficients | t | Sig. | 95,0% Confidence Interval for B | |
|  |  | B | Std. Error | Beta |  |  | Lower Bound | Upper Bound |
| 1 | (Constant) | ,414 | ,064 |  | 6,494 | ,000 | ,285 | ,543 |
|  | Physical_exercise | -,039 | ,016 | -,359 | -2,434 | ,020 | -,071 | -,007 |
| 2 | (Constant) | ,323 | ,087 |  | 3,703 | ,001 | ,147 | ,499 |
|  | Physical_exercise | -,034 | ,016 | -,314 | -2,112 | ,041 | -,066 | -,001 |
|  | Depression_severity | ,003 | ,002 | ,223 | 1,500 | ,142 | -,001 | ,007 |
| 3 | (Constant) | ,254 | ,093 |  | 2,736 | ,009 | ,066 | ,442 |
|  | Physical_exercise | -,042 | ,016 | -,391 | -2,597 | ,013 | -,075 | -,009 |
|  | Depression_severity | ,001 | ,002 | ,104 | ,653 | ,518 | -,003 | ,006 |
|  | Early_life_stress | ,003 | ,001 | ,286 | 1,809 | ,078 | ,000 | ,005 |
| a. Dependent Variable: LDL_C_HDL_C_ratio | | | | | | | | |

| **Model Summary** | | | | | | | | | |
| --- | --- | --- | --- | --- | --- | --- | --- | --- | --- |
| Model | R | R Square | Adjusted R Square | Std. Error of the Estimate | Change Statistics | | | | |
|  |  |  |  |  | R Square Change | F Change | df1 | df2 | Sig. F Change |
| 1 | ,355^a^ | ,126 | ,104 | ,10403 | ,126 | 5,770 | 1 | 40 | ,021 |
| 2 | ,426^b^ | ,181 | ,139 | ,10196 | ,055 | 2,637 | 1 | 39 | ,112 |
| 3 | ,516^c^ | ,266 | ,208 | ,09779 | ,085 | 4,396 | 1 | 38 | ,043 |
| a. Predictors: (Constant), Physical_exercise | | | | | | | | | |
| b. Predictors: (Constant), Physical_exercise, Depression_severity | | | | | | | | | |
| c. Predictors: (Constant), Physical_exercise, Depression_severity, Early_life_stress | | | | | | | | | |

| **Coefficients^a^** | | | | | | | | |
| --- | --- | --- | --- | --- | --- | --- | --- | --- |
| Model | | Unstandardized Coefficients | | Standardized Coefficients | t | Sig. | 95,0% Confidence Interval for B | |
|  |  | B | Std. Error | Beta |  |  | Lower Bound | Upper Bound |
| 1 | (Constant) | ,620 | ,048 |  | 12,823 | ,000 | ,522 | ,718 |
|  | Physical_exercise | -,029 | ,012 | -,355 | -2,402 | ,021 | -,054 | -,005 |
| 2 | (Constant) | ,546 | ,066 |  | 8,286 | ,000 | ,413 | ,679 |
|  | Physical_exercise | -,025 | ,012 | -,306 | -2,066 | ,046 | -,050 | -,001 |
|  | Depression_severity | ,003 | ,002 | ,240 | 1,624 | ,112 | -,001 | ,006 |
| 3 | (Constant) | ,486 | ,069 |  | 7,024 | ,000 | ,346 | ,626 |
|  | Physical_exercise | -,032 | ,012 | -,394 | -2,662 | ,011 | -,057 | -,008 |
|  | Depression_severity | ,001 | ,002 | ,104 | ,669 | ,507 | -,002 | ,004 |
|  | Early_life_stress | ,002 | ,001 | ,326 | 2,097 | ,043 | ,000 | ,004 |
| a. Dependent Variable: TC_HDL_C_ratio | | | | | | | | |

## Supplementary Table 3.

## Hierarchical linear regression analyses of trauma types (CTQ sub-scores) as predictors of serum lipid and lipoprotein levels in the entire MDD group

| **Model Summary** | | | | | | | | | |
| --- | --- | --- | --- | --- | --- | --- | --- | --- | --- |
| Model | R | R Square | Adjusted R Square | Std. Error of the Estimate | Change Statistics | | | | |
|  |  |  |  |  | R Square Change | F Change | df1 | df2 | Sig. F Change |
| 1 | ,396^a^ | ,157 | ,136 | 1,00399 | ,157 | 7,451 | 1 | 40 | ,009 |
| 2 | ,552^b^ | ,304 | ,269 | ,92373 | ,147 | 8,253 | 1 | 39 | ,007 |
| 3 | ,571^c^ | ,326 | ,273 | ,92118 | ,022 | 1,216 | 1 | 38 | ,277 |
| a. Predictors: (Constant), Age | | | | | | | | | |
| b. Predictors: (Constant), Age, Body_mass_index | | | | | | | | | |
| c. Predictors: (Constant), Age, Body_mass_index, Physical_neglect | | | | | | | | | |

| **Coefficients^a^** | | | | | | | | |
| --- | --- | --- | --- | --- | --- | --- | --- | --- |
| Model | | Unstandardized Coefficients | | Standardized Coefficients | t | Sig. | 95,0% Confidence Interval for B | |
|  |  | B | Std. Error | Beta |  |  | Lower Bound | Upper Bound |
| 1 | (Constant) | 3,540 | ,591 |  | 5,988 | ,000 | 2,345 | 4,735 |
|  | Age | ,044 | ,016 | ,396 | 2,730 | ,009 | ,011 | ,077 |
| 2 | (Constant) | 1,635 | ,857 |  | 1,907 | ,064 | -,099 | 3,370 |
|  | Age | ,042 | ,015 | ,375 | 2,801 | ,008 | ,012 | ,072 |
|  | Body_mass_index | ,083 | ,029 | ,384 | 2,873 | ,007 | ,025 | ,141 |
| 3 | (Constant) | 1,173 | ,952 |  | 1,231 | ,226 | -,755 | 3,101 |
|  | Age | ,039 | ,015 | ,354 | 2,627 | ,012 | ,009 | ,070 |
|  | Body_mass_index | ,088 | ,029 | ,408 | 3,021 | ,004 | ,029 | ,147 |
|  | Physical_neglect | ,044 | ,040 | ,150 | 1,103 | ,277 | -,037 | ,126 |
| a. Dependent Variable: Total_cholesterol | | | | | | | | |

| **Model Summary** | | | | | | | | | |
| --- | --- | --- | --- | --- | --- | --- | --- | --- | --- |
| Model | R | R Square | Adjusted R Square | Std. Error of the Estimate | Change Statistics | | | | |
|  |  |  |  |  | R Square Change | F Change | df1 | df2 | Sig. F Change |
| 1 | ,396^a^ | ,157 | ,136 | 1,00399 | ,157 | 7,451 | 1 | 40 | ,009 |
| 2 | ,552^b^ | ,304 | ,269 | ,92373 | ,147 | 8,253 | 1 | 39 | ,007 |
| 3 | ,575^c^ | ,330 | ,278 | ,91807 | ,026 | 1,483 | 1 | 38 | ,231 |
| a. Predictors: (Constant), Age | | | | | | | | | |
| b. Predictors: (Constant), Age, Body_mass_index | | | | | | | | | |
| c. Predictors: (Constant), Age, Body_mass_index, Physical_abuse | | | | | | | | | |

| **Coefficients^a^** | | | | | | | | |
| --- | --- | --- | --- | --- | --- | --- | --- | --- |
| Model | | Unstandardized Coefficients | | Standardized Coefficients | t | Sig. | 95,0% Confidence Interval for B | |
|  |  | B | Std. Error | Beta |  |  | Lower Bound | Upper Bound |
| 1 | (Constant) | 3,540 | ,591 |  | 5,988 | ,000 | 2,345 | 4,735 |
|  | Age | ,044 | ,016 | ,396 | 2,730 | ,009 | ,011 | ,077 |
| 2 | (Constant) | 1,635 | ,857 |  | 1,907 | ,064 | -,099 | 3,370 |
|  | Age | ,042 | ,015 | ,375 | 2,801 | ,008 | ,012 | ,072 |
|  | Body_mass_index | ,083 | ,029 | ,384 | 2,873 | ,007 | ,025 | ,141 |
| 3 | (Constant) | 2,067 | ,923 |  | 2,239 | ,031 | ,199 | 3,936 |
|  | Age | ,045 | ,015 | ,402 | 2,980 | ,005 | ,014 | ,075 |
|  | Body_mass_index | ,077 | ,029 | ,358 | 2,662 | ,011 | ,018 | ,136 |
|  | Physical_abuse | -,048 | ,040 | -,166 | -1,218 | ,231 | -,129 | ,032 |
| a. Dependent Variable: Total_cholesterol | | | | | | | | |

| **Model Summary** | | | | | | | | | |
| --- | --- | --- | --- | --- | --- | --- | --- | --- | --- |
| Model | R | R Square | Adjusted R Square | Std. Error of the Estimate | Change Statistics | | | | |
|  |  |  |  |  | R Square Change | F Change | df1 | df2 | Sig. F Change |
| 1 | ,396^a^ | ,157 | ,136 | 1,00399 | ,157 | 7,451 | 1 | 40 | ,009 |
| 2 | ,552^b^ | ,304 | ,269 | ,92373 | ,147 | 8,253 | 1 | 39 | ,007 |
| 3 | ,578^c^ | ,334 | ,282 | ,91537 | ,030 | 1,716 | 1 | 38 | ,198 |
| a. Predictors: (Constant), Age | | | | | | | | | |
| b. Predictors: (Constant), Age, Body_mass_index | | | | | | | | | |
| c. Predictors: (Constant), Age, Body_mass_index, Emotional_neglect | | | | | | | | | |

| **Coefficients^a^** | | | | | | | | |
| --- | --- | --- | --- | --- | --- | --- | --- | --- |
| Model | | Unstandardized Coefficients | | Standardized Coefficients | t | Sig. | 95,0% Confidence Interval for B | |
|  |  | B | Std. Error | Beta |  |  | Lower Bound | Upper Bound |
| 1 | (Constant) | 3,540 | ,591 |  | 5,988 | ,000 | 2,345 | 4,735 |
|  | Age | ,044 | ,016 | ,396 | 2,730 | ,009 | ,011 | ,077 |
| 2 | (Constant) | 1,635 | ,857 |  | 1,907 | ,064 | -,099 | 3,370 |
|  | Age | ,042 | ,015 | ,375 | 2,801 | ,008 | ,012 | ,072 |
|  | Body_mass_index | ,083 | ,029 | ,384 | 2,873 | ,007 | ,025 | ,141 |
| 3 | (Constant) | ,976 | ,988 |  | ,988 | ,330 | -1,024 | 2,975 |
|  | Age | ,040 | ,015 | ,360 | 2,710 | ,010 | ,010 | ,070 |
|  | Body_mass_index | ,088 | ,029 | ,409 | 3,052 | ,004 | ,030 | ,146 |
|  | Emotional_neglect | ,038 | ,029 | ,176 | 1,310 | ,198 | -,021 | ,097 |
| a. Dependent Variable: Total_cholesterol | | | | | | | | |

| **Model Summary** | | | | | | | | | |
| --- | --- | --- | --- | --- | --- | --- | --- | --- | --- |
| Model | R | R Square | Adjusted R Square | Std. Error of the Estimate | Change Statistics | | | | |
|  |  |  |  |  | R Square Change | F Change | df1 | df2 | Sig. F Change |
| 1 | ,396^a^ | ,157 | ,136 | 1,00399 | ,157 | 7,451 | 1 | 40 | ,009 |
| 2 | ,552^b^ | ,304 | ,269 | ,92373 | ,147 | 8,253 | 1 | 39 | ,007 |
| 3 | ,558^c^ | ,312 | ,257 | ,93088 | ,007 | ,403 | 1 | 38 | ,529 |
| a. Predictors: (Constant), Age | | | | | | | | | |
| b. Predictors: (Constant), Age, Body_mass_index | | | | | | | | | |
| c. Predictors: (Constant), Age, Body_mass_index, Emotional_abuse | | | | | | | | | |

| **Coefficients^a^** | | | | | | | | |
| --- | --- | --- | --- | --- | --- | --- | --- | --- |
| Model | | Unstandardized Coefficients | | Standardized Coefficients | t | Sig. | 95,0% Confidence Interval for B | |
|  |  | B | Std. Error | Beta |  |  | Lower Bound | Upper Bound |
| 1 | (Constant) | 3,540 | ,591 |  | 5,988 | ,000 | 2,345 | 4,735 |
|  | Age | ,044 | ,016 | ,396 | 2,730 | ,009 | ,011 | ,077 |
| 2 | (Constant) | 1,635 | ,857 |  | 1,907 | ,064 | -,099 | 3,370 |
|  | Age | ,042 | ,015 | ,375 | 2,801 | ,008 | ,012 | ,072 |
|  | Body_mass_index | ,083 | ,029 | ,384 | 2,873 | ,007 | ,025 | ,141 |
| 3 | (Constant) | 1,942 | ,990 |  | 1,962 | ,057 | -,062 | 3,945 |
|  | Age | ,041 | ,015 | ,370 | 2,741 | ,009 | ,011 | ,071 |
|  | Body_mass_index | ,080 | ,029 | ,372 | 2,731 | ,010 | ,021 | ,140 |
|  | Emotional_abuse | -,016 | ,025 | -,087 | -,635 | ,529 | -,067 | ,035 |
| a. Dependent Variable: Total_cholesterol | | | | | | | | |

| **Model Summary** | | | | | | | | | |
| --- | --- | --- | --- | --- | --- | --- | --- | --- | --- |
| Model | R | R Square | Adjusted R Square | Std. Error of the Estimate | Change Statistics | | | | |
|  |  |  |  |  | R Square Change | F Change | df1 | df2 | Sig. F Change |
| 1 | ,396^a^ | ,157 | ,136 | 1,00399 | ,157 | 7,451 | 1 | 40 | ,009 |
| 2 | ,552^b^ | ,304 | ,269 | ,92373 | ,147 | 8,253 | 1 | 39 | ,007 |
| 3 | ,598^c^ | ,358 | ,307 | ,89912 | ,053 | 3,164 | 1 | 38 | ,083 |
| a. Predictors: (Constant), Age | | | | | | | | | |
| b. Predictors: (Constant), Age, Body_mass_index | | | | | | | | | |
| c. Predictors: (Constant), Age, Body_mass_index, Sexual_abuse | | | | | | | | | |

| **Coefficients^a^** | | | | | | | | |
| --- | --- | --- | --- | --- | --- | --- | --- | --- |
| Model | | Unstandardized Coefficients | | Standardized Coefficients | t | Sig. | 95,0% Confidence Interval for B | |
|  |  | B | Std. Error | Beta |  |  | Lower Bound | Upper Bound |
| 1 | (Constant) | 3,540 | ,591 |  | 5,988 | ,000 | 2,345 | 4,735 |
|  | Age | ,044 | ,016 | ,396 | 2,730 | ,009 | ,011 | ,077 |
| 2 | (Constant) | 1,635 | ,857 |  | 1,907 | ,064 | -,099 | 3,370 |
|  | Age | ,042 | ,015 | ,375 | 2,801 | ,008 | ,012 | ,072 |
|  | Body_mass_index | ,083 | ,029 | ,384 | 2,873 | ,007 | ,025 | ,141 |
| 3 | (Constant) | 1,952 | ,853 |  | 2,287 | ,028 | ,224 | 3,680 |
|  | Age | ,049 | ,015 | ,438 | 3,246 | ,002 | ,018 | ,079 |
|  | Body_mass_index | ,078 | ,028 | ,363 | 2,776 | ,008 | ,021 | ,135 |
|  | Sexual_abuse | -,060 | ,034 | -,240 | -1,779 | ,083 | -,129 | ,008 |
| a. Dependent Variable: Total_cholesterol | | | | | | | | |

| **Model Summary** | | | | | | | | | |
| --- | --- | --- | --- | --- | --- | --- | --- | --- | --- |
| Model | R | R Square | Adjusted R Square | Std. Error of the Estimate | Change Statistics | | | | |
|  |  |  |  |  | R Square Change | F Change | df1 | df2 | Sig. F Change |
| 1 | ,328^a^ | ,108 | ,085 | ,18104 | ,108 | 4,830 | 1 | 40 | ,034 |
| 2 | ,481^b^ | ,231 | ,191 | ,17022 | ,123 | 6,244 | 1 | 39 | ,017 |
| a. Predictors: (Constant), Physical_exercise | | | | | | | | | |
| b. Predictors: (Constant), Physical_exercise, Physical_neglect | | | | | | | | | |

| **Coefficients^a^** | | | | | | | | |
| --- | --- | --- | --- | --- | --- | --- | --- | --- |
| Model | | Unstandardized Coefficients | | Standardized Coefficients | t | Sig. | 95,0% Confidence Interval for B | |
|  |  | B | Std. Error | Beta |  |  | Lower Bound | Upper Bound |
| 1 | (Constant) | ,228 | ,084 |  | 2,712 | ,010 | ,058 | ,398 |
|  | Physical_exercise | -,046 | ,021 | -,328 | -2,198 | ,034 | -,089 | -,004 |
| 2 | (Constant) | ,061 | ,104 |  | ,585 | ,562 | -,149 | ,270 |
|  | Physical_exercise | -,047 | ,020 | -,336 | -2,393 | ,022 | -,088 | -,007 |
|  | Physical_neglect | ,018 | ,007 | ,351 | 2,499 | ,017 | ,003 | ,033 |
| a. Dependent Variable: Triglycerides | | | | | | | | |

| **Model Summary** | | | | | | | | | |
| --- | --- | --- | --- | --- | --- | --- | --- | --- | --- |
| Model | R | R Square | Adjusted R Square | Std. Error of the Estimate | Change Statistics | | | | |
|  |  |  |  |  | R Square Change | F Change | df1 | df2 | Sig. F Change |
| 1 | ,328^a^ | ,108 | ,085 | ,18104 | ,108 | 4,830 | 1 | 40 | ,034 |
| 2 | ,458^b^ | ,209 | ,169 | ,17259 | ,102 | 5,010 | 1 | 39 | ,031 |
| a. Predictors: (Constant), Physical_exercise | | | | | | | | | |
| b. Predictors: (Constant), Physical_exercise, Physical_abuse | | | | | | | | | |

| **Coefficients^a^** | | | | | | | | |
| --- | --- | --- | --- | --- | --- | --- | --- | --- |
| Model | | Unstandardized Coefficients | | Standardized Coefficients | t | Sig. | 95,0% Confidence Interval for B | |
|  |  | B | Std. Error | Beta |  |  | Lower Bound | Upper Bound |
| 1 | (Constant) | ,228 | ,084 |  | 2,712 | ,010 | ,058 | ,398 |
|  | Physical_exercise | -,046 | ,021 | -,328 | -2,198 | ,034 | -,089 | -,004 |
| 2 | (Constant) | ,105 | ,097 |  | 1,084 | ,285 | -,091 | ,302 |
|  | Physical_exercise | -,050 | ,020 | -,354 | -2,478 | ,018 | -,091 | -,009 |
|  | Physical_abuse | ,016 | ,007 | ,320 | 2,238 | ,031 | ,002 | ,031 |
| a. Dependent Variable: Triglycerides | | | | | | | | |

| **Model Summary** | | | | | | | | | |
| --- | --- | --- | --- | --- | --- | --- | --- | --- | --- |
| Model | R | R Square | Adjusted R Square | Std. Error of the Estimate | Change Statistics | | | | |
|  |  |  |  |  | R Square Change | F Change | df1 | df2 | Sig. F Change |
| 1 | ,328^a^ | ,108 | ,085 | ,18104 | ,108 | 4,830 | 1 | 40 | ,034 |
| 2 | ,500^b^ | ,250 | ,212 | ,16807 | ,142 | 7,412 | 1 | 39 | ,010 |
| a. Predictors: (Constant), Physical_exercise | | | | | | | | | |
| b. Predictors: (Constant), Physical_exercise, Emotional_neglect | | | | | | | | | |

| **Coefficients^a^** | | | | | | | | |
| --- | --- | --- | --- | --- | --- | --- | --- | --- |
| Model | | Unstandardized Coefficients | | Standardized Coefficients | t | Sig. | 95,0% Confidence Interval for B | |
|  |  | B | Std. Error | Beta |  |  | Lower Bound | Upper Bound |
| 1 | (Constant) | ,228 | ,084 |  | 2,712 | ,010 | ,058 | ,398 |
|  | Physical_exercise | -,046 | ,021 | -,328 | -2,198 | ,034 | -,089 | -,004 |
| 2 | (Constant) | ,030 | ,107 |  | ,281 | ,780 | -,186 | ,246 |
|  | Physical_exercise | -,053 | ,020 | -,378 | -2,702 | ,010 | -,093 | -,013 |
|  | Emotional_neglect | ,015 | ,005 | ,381 | 2,722 | ,010 | ,004 | ,025 |
| a. Dependent Variable: Triglycerides | | | | | | | | |

| **Model Summary** | | | | | | | | | |
| --- | --- | --- | --- | --- | --- | --- | --- | --- | --- |
| Model | R | R Square | Adjusted R Square | Std. Error of the Estimate | Change Statistics | | | | |
|  |  |  |  |  | R Square Change | F Change | df1 | df2 | Sig. F Change |
| 1 | ,328^a^ | ,108 | ,085 | ,18104 | ,108 | 4,830 | 1 | 40 | ,034 |
| 2 | ,447^b^ | ,200 | ,158 | ,17366 | ,092 | 4,471 | 1 | 39 | ,041 |
| a. Predictors: (Constant), Physical_exercise | | | | | | | | | |
| b. Predictors: (Constant), Physical_exercise, Emotional_abuse | | | | | | | | | |

| **Coefficients^a^** | | | | | | | | |
| --- | --- | --- | --- | --- | --- | --- | --- | --- |
| Model | | Unstandardized Coefficients | | Standardized Coefficients | t | Sig. | 95,0% Confidence Interval for B | |
|  |  | B | Std. Error | Beta |  |  | Lower Bound | Upper Bound |
| 1 | (Constant) | ,228 | ,084 |  | 2,712 | ,010 | ,058 | ,398 |
|  | Physical_exercise | -,046 | ,021 | -,328 | -2,198 | ,034 | -,089 | -,004 |
| 2 | (Constant) | ,117 | ,096 |  | 1,213 | ,232 | -,078 | ,312 |
|  | Physical_exercise | -,054 | ,021 | -,382 | -2,623 | ,012 | -,095 | -,012 |
|  | Emotional_abuse | ,010 | ,005 | ,308 | 2,115 | ,041 | ,000 | ,019 |
| a. Dependent Variable: Triglycerides | | | | | | | | |

| **Model Summary** | | | | | | | | | |
| --- | --- | --- | --- | --- | --- | --- | --- | --- | --- |
| Model | R | R Square | Adjusted R Square | Std. Error of the Estimate | Change Statistics | | | | |
|  |  |  |  |  | R Square Change | F Change | df1 | df2 | Sig. F Change |
| 1 | ,328^a^ | ,108 | ,085 | ,18104 | ,108 | 4,830 | 1 | 40 | ,034 |
| 2 | ,347^b^ | ,120 | ,075 | ,18207 | ,012 | ,549 | 1 | 39 | ,463 |
| a. Predictors: (Constant), Physical_exercise | | | | | | | | | |
| b. Predictors: (Constant), Physical_exercise, Sexual_abuse | | | | | | | | | |

| **Coefficients^a^** | | | | | | | | |
| --- | --- | --- | --- | --- | --- | --- | --- | --- |
| Model | | Unstandardized Coefficients | | Standardized Coefficients | t | Sig. | 95,0% Confidence Interval for B | |
|  |  | B | Std. Error | Beta |  |  | Lower Bound | Upper Bound |
| 1 | (Constant) | ,228 | ,084 |  | 2,712 | ,010 | ,058 | ,398 |
|  | Physical_exercise | -,046 | ,021 | -,328 | -2,198 | ,034 | -,089 | -,004 |
| 2 | (Constant) | ,204 | ,091 |  | 2,254 | ,030 | ,021 | ,388 |
|  | Physical_exercise | -,050 | ,022 | -,355 | -2,298 | ,027 | -,094 | -,006 |
|  | Sexual_abuse | ,005 | ,007 | ,114 | ,741 | ,463 | -,009 | ,019 |
| a. Dependent Variable: Triglycerides | | | | | | | | |

| **Model Summary** | | | | | | | | | |
| --- | --- | --- | --- | --- | --- | --- | --- | --- | --- |
| Model | R | R Square | Adjusted R Square | Std. Error of the Estimate | Change Statistics | | | | |
|  |  |  |  |  | R Square Change | F Change | df1 | df2 | Sig. F Change |
| 1 | ,395^a^ | ,156 | ,135 | ,34075 | ,156 | 7,388 | 1 | 40 | ,010 |
| 2 | ,499^b^ | ,249 | ,210 | ,32553 | ,093 | 4,827 | 1 | 39 | ,034 |
| a. Predictors: (Constant), Depression_severity | | | | | | | | | |
| b. Predictors: (Constant), Depression_severity, Physical_neglect | | | | | | | | | |

| **Coefficients^a^** | | | | | | | | |
| --- | --- | --- | --- | --- | --- | --- | --- | --- |
| Model | | Unstandardized Coefficients | | Standardized Coefficients | t | Sig. | 95,0% Confidence Interval for B | |
|  |  | B | Std. Error | Beta |  |  | Lower Bound | Upper Bound |
| 1 | (Constant) | 1,902 | ,130 |  | 14,624 | ,000 | 1,639 | 2,165 |
|  | Depression_severity | -,014 | ,005 | -,395 | -2,718 | ,010 | -,024 | -,004 |
| 2 | (Constant) | 2,169 | ,174 |  | 12,492 | ,000 | 1,818 | 2,520 |
|  | Depression_severity | -,013 | ,005 | -,366 | -2,625 | ,012 | -,023 | -,003 |
|  | Physical_neglect | -,031 | ,014 | -,306 | -2,197 | ,034 | -,059 | -,002 |
| a. Dependent Variable: HDL_cholesterol | | | | | | | | |

| **Model Summary** | | | | | | | | | |
| --- | --- | --- | --- | --- | --- | --- | --- | --- | --- |
| Model | R | R Square | Adjusted R Square | Std. Error of the Estimate | Change Statistics | | | | |
|  |  |  |  |  | R Square Change | F Change | df1 | df2 | Sig. F Change |
| 1 | ,395^a^ | ,156 | ,135 | ,34075 | ,156 | 7,388 | 1 | 40 | ,010 |
| 2 | ,496^b^ | ,246 | ,208 | ,32606 | ,091 | 4,686 | 1 | 39 | ,037 |
| a. Predictors: (Constant), Depression_severity | | | | | | | | | |
| b. Predictors: (Constant), Depression_severity, Physical_abuse | | | | | | | | | |

| **Coefficients^a^** | | | | | | | | |
| --- | --- | --- | --- | --- | --- | --- | --- | --- |
| Model | | Unstandardized Coefficients | | Standardized Coefficients | t | Sig. | 95,0% Confidence Interval for B | |
|  |  | B | Std. Error | Beta |  |  | Lower Bound | Upper Bound |
| 1 | (Constant) | 1,902 | ,130 |  | 14,624 | ,000 | 1,639 | 2,165 |
|  | Depression_severity | -,014 | ,005 | -,395 | -2,718 | ,010 | -,024 | -,004 |
| 2 | (Constant) | 2,118 | ,159 |  | 13,282 | ,000 | 1,796 | 2,441 |
|  | Depression_severity | -,012 | ,005 | -,352 | -2,503 | ,017 | -,022 | -,002 |
|  | Physical_abuse | -,030 | ,014 | -,304 | -2,165 | ,037 | -,058 | -,002 |
| a. Dependent Variable: HDL_cholesterol | | | | | | | | |

| **Model Summary** | | | | | | | | | |
| --- | --- | --- | --- | --- | --- | --- | --- | --- | --- |
| Model | R | R Square | Adjusted R Square | Std. Error of the Estimate | Change Statistics | | | | |
|  |  |  |  |  | R Square Change | F Change | df1 | df2 | Sig. F Change |
| 1 | ,395^a^ | ,156 | ,135 | ,34075 | ,156 | 7,388 | 1 | 40 | ,010 |
| 2 | ,439^b^ | ,193 | ,152 | ,33742 | ,037 | 1,793 | 1 | 39 | ,188 |
| a. Predictors: (Constant), Depression_severity | | | | | | | | | |
| b. Predictors: (Constant), Depression_severity, Emotional_neglect | | | | | | | | | |

| **Coefficients^a^** | | | | | | | | |
| --- | --- | --- | --- | --- | --- | --- | --- | --- |
| Model | | Unstandardized Coefficients | | Standardized Coefficients | t | Sig. | 95,0% Confidence Interval for B | |
|  |  | B | Std. Error | Beta |  |  | Lower Bound | Upper Bound |
| 1 | (Constant) | 1,902 | ,130 |  | 14,624 | ,000 | 1,639 | 2,165 |
|  | Depression_severity | -,014 | ,005 | -,395 | -2,718 | ,010 | -,024 | -,004 |
| 2 | (Constant) | 2,086 | ,188 |  | 11,084 | ,000 | 1,705 | 2,467 |
|  | Depression_severity | -,012 | ,005 | -,340 | -2,274 | ,029 | -,023 | -,001 |
|  | Emotional_neglect | -,015 | ,011 | -,200 | -1,339 | ,188 | -,037 | ,008 |
| a. Dependent Variable: HDL_cholesterol | | | | | | | | |

| **Model Summary** | | | | | | | | | |
| --- | --- | --- | --- | --- | --- | --- | --- | --- | --- |
| Model | R | R Square | Adjusted R Square | Std. Error of the Estimate | Change Statistics | | | | |
|  |  |  |  |  | R Square Change | F Change | df1 | df2 | Sig. F Change |
| 1 | ,395^a^ | ,156 | ,135 | ,34075 | ,156 | 7,388 | 1 | 40 | ,010 |
| 2 | ,483^b^ | ,233 | ,194 | ,32888 | ,077 | 3,939 | 1 | 39 | ,054 |
| a. Predictors: (Constant), Depression_severity | | | | | | | | | |
| b. Predictors: (Constant), Depression_severity, Emotional_abuse | | | | | | | | | |

| **Coefficients^a^** | | | | | | | | |
| --- | --- | --- | --- | --- | --- | --- | --- | --- |
| Model | | Unstandardized Coefficients | | Standardized Coefficients | t | Sig. | 95,0% Confidence Interval for B | |
|  |  | B | Std. Error | Beta |  |  | Lower Bound | Upper Bound |
| 1 | (Constant) | 1,902 | ,130 |  | 14,624 | ,000 | 1,639 | 2,165 |
|  | Depression_severity | -,014 | ,005 | -,395 | -2,718 | ,010 | -,024 | -,004 |
| 2 | (Constant) | 2,088 | ,156 |  | 13,341 | ,000 | 1,771 | 2,404 |
|  | Depression_severity | -,011 | ,005 | -,309 | -2,103 | ,042 | -,021 | ,000 |
|  | Emotional_abuse | -,018 | ,009 | -,291 | -1,985 | ,054 | -,037 | ,000 |
| a. Dependent Variable: HDL_cholesterol | | | | | | | | |

| **Model Summary** | | | | | | | | | |
| --- | --- | --- | --- | --- | --- | --- | --- | --- | --- |
| Model | R | R Square | Adjusted R Square | Std. Error of the Estimate | Change Statistics | | | | |
|  |  |  |  |  | R Square Change | F Change | df1 | df2 | Sig. F Change |
| 1 | ,395^a^ | ,156 | ,135 | ,34075 | ,156 | 7,388 | 1 | 40 | ,010 |
| 2 | ,397^b^ | ,158 | ,115 | ,34469 | ,002 | ,091 | 1 | 39 | ,764 |
| a. Predictors: (Constant), Depression_severity | | | | | | | | | |
| b. Predictors: (Constant), Depression_severity, Sexual_abuse | | | | | | | | | |

| **Coefficients^a^** | | | | | | | | |
| --- | --- | --- | --- | --- | --- | --- | --- | --- |
| Model | | Unstandardized Coefficients | | Standardized Coefficients | t | Sig. | 95,0% Confidence Interval for B | |
|  |  | B | Std. Error | Beta |  |  | Lower Bound | Upper Bound |
| 1 | (Constant) | 1,902 | ,130 |  | 14,624 | ,000 | 1,639 | 2,165 |
|  | Depression_severity | -,014 | ,005 | -,395 | -2,718 | ,010 | -,024 | -,004 |
| 2 | (Constant) | 1,889 | ,139 |  | 13,579 | ,000 | 1,607 | 2,170 |
|  | Depression_severity | -,015 | ,006 | -,417 | -2,532 | ,015 | -,026 | -,003 |
|  | Sexual_abuse | ,004 | ,014 | ,050 | ,302 | ,764 | -,024 | ,033 |
| a. Dependent Variable: HDL_cholesterol | | | | | | | | |

| **Model Summary** | | | | | | | | | |
| --- | --- | --- | --- | --- | --- | --- | --- | --- | --- |
| Model | R | R Square | Adjusted R Square | Std. Error of the Estimate | Change Statistics | | | | |
|  |  |  |  |  | R Square Change | F Change | df1 | df2 | Sig. F Change |
| 1 | ,388^a^ | ,151 | ,130 | ,74086 | ,151 | 7,108 | 1 | 40 | ,011 |
| 2 | ,564^b^ | ,318 | ,283 | ,67256 | ,167 | 9,537 | 1 | 39 | ,004 |
| 3 | ,591^c^ | ,349 | ,298 | ,66538 | ,032 | 1,846 | 1 | 38 | ,182 |
| a. Predictors: (Constant), Age | | | | | | | | | |
| b. Predictors: (Constant), Age, Physical_exercise | | | | | | | | | |
| c. Predictors: (Constant), Age, Physical_exercise, Physical_neglect | | | | | | | | | |

| **Coefficients^a^** | | | | | | | | |
| --- | --- | --- | --- | --- | --- | --- | --- | --- |
| Model | | Unstandardized Coefficients | | Standardized Coefficients | t | Sig. | 95,0% Confidence Interval for B | |
|  |  | B | Std. Error | Beta |  |  | Lower Bound | Upper Bound |
| 1 | (Constant) | 1,830 | ,436 |  | 4,194 | ,000 | ,948 | 2,711 |
|  | Age | ,032 | ,012 | ,388 | 2,666 | ,011 | ,008 | ,056 |
| 2 | (Constant) | 2,718 | ,490 |  | 5,553 | ,000 | 1,728 | 3,708 |
|  | Age | ,032 | ,011 | ,396 | 2,994 | ,005 | ,010 | ,054 |
|  | Physical_exercise | -,242 | ,078 | -,409 | -3,088 | ,004 | -,401 | -,084 |
| 3 | (Constant) | 2,425 | ,530 |  | 4,573 | ,000 | 1,351 | 3,498 |
|  | Age | ,030 | ,011 | ,373 | 2,827 | ,007 | ,009 | ,052 |
|  | Physical_exercise | -,244 | ,078 | -,412 | -3,148 | ,003 | -,401 | -,087 |
|  | Physical_neglect | ,039 | ,029 | ,179 | 1,359 | ,182 | -,019 | ,097 |
| a. Dependent Variable: LDL_cholesterol | | | | | | | | |

| **Model Summary** | | | | | | | | | |
| --- | --- | --- | --- | --- | --- | --- | --- | --- | --- |
| Model | R | R Square | Adjusted R Square | Std. Error of the Estimate | Change Statistics | | | | |
|  |  |  |  |  | R Square Change | F Change | df1 | df2 | Sig. F Change |
| 1 | ,388^a^ | ,151 | ,130 | ,74086 | ,151 | 7,108 | 1 | 40 | ,011 |
| 2 | ,564^b^ | ,318 | ,283 | ,67256 | ,167 | 9,537 | 1 | 39 | ,004 |
| 3 | ,570^c^ | ,325 | ,272 | ,67754 | ,008 | ,429 | 1 | 38 | ,516 |
| a. Predictors: (Constant), Age | | | | | | | | | |
| b. Predictors: (Constant), Age, Physical_exercise | | | | | | | | | |
| c. Predictors: (Constant), Age, Physical_exercise, Physical_abuse | | | | | | | | | |

| **Coefficients^a^** | | | | | | | | |
| --- | --- | --- | --- | --- | --- | --- | --- | --- |
| Model | | Unstandardized Coefficients | | Standardized Coefficients | t | Sig. | 95,0% Confidence Interval for B | |
|  |  | B | Std. Error | Beta |  |  | Lower Bound | Upper Bound |
| 1 | (Constant) | 1,830 | ,436 |  | 4,194 | ,000 | ,948 | 2,711 |
|  | Age | ,032 | ,012 | ,388 | 2,666 | ,011 | ,008 | ,056 |
| 2 | (Constant) | 2,718 | ,490 |  | 5,553 | ,000 | 1,728 | 3,708 |
|  | Age | ,032 | ,011 | ,396 | 2,994 | ,005 | ,010 | ,054 |
|  | Physical_exercise | -,242 | ,078 | -,409 | -3,088 | ,004 | -,401 | -,084 |
| 3 | (Constant) | 2,822 | ,518 |  | 5,447 | ,000 | 1,773 | 3,872 |
|  | Age | ,033 | ,011 | ,410 | 3,038 | ,004 | ,011 | ,056 |
|  | Physical_exercise | -,238 | ,079 | -,402 | -3,005 | ,005 | -,398 | -,078 |
|  | Physical_abuse | -,019 | ,029 | -,089 | -,655 | ,516 | -,078 | ,040 |
| a. Dependent Variable: LDL_cholesterol | | | | | | | | |

| **Model Summary** | | | | | | | | | |
| --- | --- | --- | --- | --- | --- | --- | --- | --- | --- |
| Model | R | R Square | Adjusted R Square | Std. Error of the Estimate | Change Statistics | | | | |
|  |  |  |  |  | R Square Change | F Change | df1 | df2 | Sig. F Change |
| 1 | ,388^a^ | ,151 | ,130 | ,74086 | ,151 | 7,108 | 1 | 40 | ,011 |
| 2 | ,564^b^ | ,318 | ,283 | ,67256 | ,167 | 9,537 | 1 | 39 | ,004 |
| 3 | ,596^c^ | ,355 | ,304 | ,66265 | ,037 | 2,176 | 1 | 38 | ,148 |
| a. Predictors: (Constant), Age | | | | | | | | | |
| b. Predictors: (Constant), Age, Physical_exercise | | | | | | | | | |
| c. Predictors: (Constant), Age, Physical_exercise, Emotional_neglect | | | | | | | | | |

| **Coefficients^a^** | | | | | | | | |
| --- | --- | --- | --- | --- | --- | --- | --- | --- |
| Model | | Unstandardized Coefficients | | Standardized Coefficients | t | Sig. | 95,0% Confidence Interval for B | |
|  |  | B | Std. Error | Beta |  |  | Lower Bound | Upper Bound |
| 1 | (Constant) | 1,830 | ,436 |  | 4,194 | ,000 | ,948 | 2,711 |
|  | Age | ,032 | ,012 | ,388 | 2,666 | ,011 | ,008 | ,056 |
| 2 | (Constant) | 2,718 | ,490 |  | 5,553 | ,000 | 1,728 | 3,708 |
|  | Age | ,032 | ,011 | ,396 | 2,994 | ,005 | ,010 | ,054 |
|  | Physical_exercise | -,242 | ,078 | -,409 | -3,088 | ,004 | -,401 | -,084 |
| 3 | (Constant) | 2,333 | ,548 |  | 4,254 | ,000 | 1,223 | 3,443 |
|  | Age | ,031 | ,011 | ,382 | 2,926 | ,006 | ,010 | ,053 |
|  | Physical_exercise | -,257 | ,078 | -,434 | -3,299 | ,002 | -,415 | -,099 |
|  | Emotional_neglect | ,031 | ,021 | ,194 | 1,475 | ,148 | -,012 | ,074 |
| a. Dependent Variable: LDL_cholesterol | | | | | | | | |

| **Model Summary** | | | | | | | | | |
| --- | --- | --- | --- | --- | --- | --- | --- | --- | --- |
| Model | R | R Square | Adjusted R Square | Std. Error of the Estimate | Change Statistics | | | | |
|  |  |  |  |  | R Square Change | F Change | df1 | df2 | Sig. F Change |
| 1 | ,388^a^ | ,151 | ,130 | ,74086 | ,151 | 7,108 | 1 | 40 | ,011 |
| 2 | ,564^b^ | ,318 | ,283 | ,67256 | ,167 | 9,537 | 1 | 39 | ,004 |
| 3 | ,564^c^ | ,318 | ,264 | ,68133 | ,000 | ,003 | 1 | 38 | ,956 |
| a. Predictors: (Constant), Age | | | | | | | | | |
| b. Predictors: (Constant), Age, Physical_exercise | | | | | | | | | |
| c. Predictors: (Constant), Age, Physical_exercise, Emotional_abuse | | | | | | | | | |

| **Coefficients^a^** | | | | | | | | |
| --- | --- | --- | --- | --- | --- | --- | --- | --- |
| Model | | Unstandardized Coefficients | | Standardized Coefficients | t | Sig. | 95,0% Confidence Interval for B | |
|  |  | B | Std. Error | Beta |  |  | Lower Bound | Upper Bound |
| 1 | (Constant) | 1,830 | ,436 |  | 4,194 | ,000 | ,948 | 2,711 |
|  | Age | ,032 | ,012 | ,388 | 2,666 | ,011 | ,008 | ,056 |
| 2 | (Constant) | 2,718 | ,490 |  | 5,553 | ,000 | 1,728 | 3,708 |
|  | Age | ,032 | ,011 | ,396 | 2,994 | ,005 | ,010 | ,054 |
|  | Physical_exercise | -,242 | ,078 | -,409 | -3,088 | ,004 | -,401 | -,084 |
| 3 | (Constant) | 2,705 | ,548 |  | 4,941 | ,000 | 1,597 | 3,814 |
|  | Age | ,032 | ,011 | ,397 | 2,953 | ,005 | ,010 | ,055 |
|  | Physical_exercise | -,243 | ,081 | -,410 | -3,011 | ,005 | -,406 | -,080 |
|  | Emotional_abuse | ,001 | ,018 | ,008 | ,056 | ,956 | -,036 | ,038 |
| a. Dependent Variable: LDL_cholesterol | | | | | | | | |

| **Model Summary** | | | | | | | | | |
| --- | --- | --- | --- | --- | --- | --- | --- | --- | --- |
| Model | R | R Square | Adjusted R Square | Std. Error of the Estimate | Change Statistics | | | | |
|  |  |  |  |  | R Square Change | F Change | df1 | df2 | Sig. F Change |
| 1 | ,388^a^ | ,151 | ,130 | ,74086 | ,151 | 7,108 | 1 | 40 | ,011 |
| 2 | ,564^b^ | ,318 | ,283 | ,67256 | ,167 | 9,537 | 1 | 39 | ,004 |
| 3 | ,593^c^ | ,351 | ,300 | ,66441 | ,034 | 1,963 | 1 | 38 | ,169 |
| a. Predictors: (Constant), Age | | | | | | | | | |
| b. Predictors: (Constant), Age, Physical_exercise | | | | | | | | | |
| c. Predictors: (Constant), Age, Physical_exercise, Sexual_abuse | | | | | | | | | |

| **Coefficients^a^** | | | | | | | | |
| --- | --- | --- | --- | --- | --- | --- | --- | --- |
| Model | | Unstandardized Coefficients | | Standardized Coefficients | t | Sig. | 95,0% Confidence Interval for B | |
|  |  | B | Std. Error | Beta |  |  | Lower Bound | Upper Bound |
| 1 | (Constant) | 1,830 | ,436 |  | 4,194 | ,000 | ,948 | 2,711 |
|  | Age | ,032 | ,012 | ,388 | 2,666 | ,011 | ,008 | ,056 |
| 2 | (Constant) | 2,718 | ,490 |  | 5,553 | ,000 | 1,728 | 3,708 |
|  | Age | ,032 | ,011 | ,396 | 2,994 | ,005 | ,010 | ,054 |
|  | Physical_exercise | -,242 | ,078 | -,409 | -3,088 | ,004 | -,401 | -,084 |
| 3 | (Constant) | 2,748 | ,484 |  | 5,677 | ,000 | 1,768 | 3,728 |
|  | Age | ,036 | ,011 | ,446 | 3,293 | ,002 | ,014 | ,059 |
|  | Physical_exercise | -,216 | ,080 | -,364 | -2,709 | ,010 | -,377 | -,055 |
|  | Sexual_abuse | -,036 | ,026 | -,195 | -1,401 | ,169 | -,088 | ,016 |
| a. Dependent Variable: LDL_cholesterol | | | | | | | | |

| **Model Summary** | | | | | | | | | |
| --- | --- | --- | --- | --- | --- | --- | --- | --- | --- |
| Model | R | R Square | Adjusted R Square | Std. Error of the Estimate | Change Statistics | | | | |
|  |  |  |  |  | R Square Change | F Change | df1 | df2 | Sig. F Change |
| 1 | ,359^a^ | ,129 | ,107 | ,13707 | ,129 | 5,923 | 1 | 40 | ,020 |
| 2 | ,532^b^ | ,283 | ,246 | ,12599 | ,154 | 8,351 | 1 | 39 | ,006 |
| a. Predictors: (Constant), Physical_exercise | | | | | | | | | |
| b. Predictors: (Constant), Physical_exercise, Physical_neglect | | | | | | | | | |

| **Coefficients^a^** | | | | | | | | |
| --- | --- | --- | --- | --- | --- | --- | --- | --- |
| Model | | Unstandardized Coefficients | | Standardized Coefficients | t | Sig. | 95,0% Confidence Interval for B | |
|  |  | B | Std. Error | Beta |  |  | Lower Bound | Upper Bound |
| 1 | (Constant) | ,414 | ,064 |  | 6,494 | ,000 | ,285 | ,543 |
|  | Physical_exercise | -,039 | ,016 | -,359 | -2,434 | ,020 | -,071 | -,007 |
| 2 | (Constant) | ,270 | ,077 |  | 3,522 | ,001 | ,115 | ,426 |
|  | Physical_exercise | -,040 | ,015 | -,368 | -2,712 | ,010 | -,070 | -,010 |
|  | Physical_neglect | ,016 | ,005 | ,392 | 2,890 | ,006 | ,005 | ,026 |
| a. Dependent Variable: LDL_C_HDL_C_ratio | | | | | | | | |

| **Model Summary** | | | | | | | | | |
| --- | --- | --- | --- | --- | --- | --- | --- | --- | --- |
| Model | R | R Square | Adjusted R Square | Std. Error of the Estimate | Change Statistics | | | | |
|  |  |  |  |  | R Square Change | F Change | df1 | df2 | Sig. F Change |
| 1 | ,359^a^ | ,129 | ,107 | ,13707 | ,129 | 5,923 | 1 | 40 | ,020 |
| 2 | ,411^b^ | ,169 | ,126 | ,13561 | ,040 | 1,868 | 1 | 39 | ,180 |
| a. Predictors: (Constant), Physical_exercise | | | | | | | | | |
| b. Predictors: (Constant), Physical_exercise, Physical_abuse | | | | | | | | | |

| **Coefficients^a^** | | | | | | | | |
| --- | --- | --- | --- | --- | --- | --- | --- | --- |
| Model | | Unstandardized Coefficients | | Standardized Coefficients | t | Sig. | 95,0% Confidence Interval for B | |
|  |  | B | Std. Error | Beta |  |  | Lower Bound | Upper Bound |
| 1 | (Constant) | ,414 | ,064 |  | 6,494 | ,000 | ,285 | ,543 |
|  | Physical_exercise | -,039 | ,016 | -,359 | -2,434 | ,020 | -,071 | -,007 |
| 2 | (Constant) | ,355 | ,076 |  | 4,645 | ,000 | ,200 | ,509 |
|  | Physical_exercise | -,041 | ,016 | -,375 | -2,562 | ,014 | -,073 | -,009 |
|  | Physical_abuse | ,008 | ,006 | ,200 | 1,367 | ,180 | -,004 | ,019 |
| a. Dependent Variable: LDL_C_HDL_C_ratio | | | | | | | | |

| **Model Summary** | | | | | | | | | |
| --- | --- | --- | --- | --- | --- | --- | --- | --- | --- |
| Model | R | R Square | Adjusted R Square | Std. Error of the Estimate | Change Statistics | | | | |
|  |  |  |  |  | R Square Change | F Change | df1 | df2 | Sig. F Change |
| 1 | ,359^a^ | ,129 | ,107 | ,13707 | ,129 | 5,923 | 1 | 40 | ,020 |
| 2 | ,505^b^ | ,255 | ,217 | ,12837 | ,126 | 6,606 | 1 | 39 | ,014 |
| a. Predictors: (Constant), Physical_exercise | | | | | | | | | |
| b. Predictors: (Constant), Physical_exercise, Emotional_neglect | | | | | | | | | |

| **Coefficients^a^** | | | | | | | | |
| --- | --- | --- | --- | --- | --- | --- | --- | --- |
| Model | | Unstandardized Coefficients | | Standardized Coefficients | t | Sig. | 95,0% Confidence Interval for B | |
|  |  | B | Std. Error | Beta |  |  | Lower Bound | Upper Bound |
| 1 | (Constant) | ,414 | ,064 |  | 6,494 | ,000 | ,285 | ,543 |
|  | Physical_exercise | -,039 | ,016 | -,359 | -2,434 | ,020 | -,071 | -,007 |
| 2 | (Constant) | ,271 | ,082 |  | 3,321 | ,002 | ,106 | ,436 |
|  | Physical_exercise | -,044 | ,015 | -,406 | -2,912 | ,006 | -,074 | -,013 |
|  | Emotional_neglect | ,010 | ,004 | ,358 | 2,570 | ,014 | ,002 | ,019 |
| a. Dependent Variable: LDL_C_HDL_C_ratio | | | | | | | | |

| **Model Summary** | | | | | | | | | |
| --- | --- | --- | --- | --- | --- | --- | --- | --- | --- |
| Model | R | R Square | Adjusted R Square | Std. Error of the Estimate | Change Statistics | | | | |
|  |  |  |  |  | R Square Change | F Change | df1 | df2 | Sig. F Change |
| 1 | ,359^a^ | ,129 | ,107 | ,13707 | ,129 | 5,923 | 1 | 40 | ,020 |
| 2 | ,421^b^ | ,177 | ,135 | ,13491 | ,048 | 2,295 | 1 | 39 | ,138 |
| a. Predictors: (Constant), Physical_exercise | | | | | | | | | |
| b. Predictors: (Constant), Physical_exercise, Emotional_abuse | | | | | | | | | |

| **Coefficients^a^** | | | | | | | | |
| --- | --- | --- | --- | --- | --- | --- | --- | --- |
| Model | | Unstandardized Coefficients | | Standardized Coefficients | t | Sig. | 95,0% Confidence Interval for B | |
|  |  | B | Std. Error | Beta |  |  | Lower Bound | Upper Bound |
| 1 | (Constant) | ,414 | ,064 |  | 6,494 | ,000 | ,285 | ,543 |
|  | Physical_exercise | -,039 | ,016 | -,359 | -2,434 | ,020 | -,071 | -,007 |
| 2 | (Constant) | ,352 | ,075 |  | 4,698 | ,000 | ,200 | ,503 |
|  | Physical_exercise | -,043 | ,016 | -,398 | -2,698 | ,010 | -,075 | -,011 |
|  | Emotional_abuse | ,006 | ,004 | ,223 | 1,515 | ,138 | -,002 | ,013 |
| a. Dependent Variable: LDL_C_HDL_C_ratio | | | | | | | | |

| **Model Summary** | | | | | | | | | |
| --- | --- | --- | --- | --- | --- | --- | --- | --- | --- |
| Model | R | R Square | Adjusted R Square | Std. Error of the Estimate | Change Statistics | | | | |
|  |  |  |  |  | R Square Change | F Change | df1 | df2 | Sig. F Change |
| 1 | ,359^a^ | ,129 | ,107 | ,13707 | ,129 | 5,923 | 1 | 40 | ,020 |
| 2 | ,359^b^ | ,129 | ,084 | ,13882 | ,000 | ,000 | 1 | 39 | ,984 |
| a. Predictors: (Constant), Physical_exercise | | | | | | | | | |
| b. Predictors: (Constant), Physical_exercise, Sexual_abuse | | | | | | | | | |

| **Coefficients^a^** | | | | | | | | |
| --- | --- | --- | --- | --- | --- | --- | --- | --- |
| Model | | Unstandardized Coefficients | | Standardized Coefficients | t | Sig. | 95,0% Confidence Interval for B | |
|  |  | B | Std. Error | Beta |  |  | Lower Bound | Upper Bound |
| 1 | (Constant) | ,414 | ,064 |  | 6,494 | ,000 | ,285 | ,543 |
|  | Physical_exercise | -,039 | ,016 | -,359 | -2,434 | ,020 | -,071 | -,007 |
| 2 | (Constant) | ,413 | ,069 |  | 5,981 | ,000 | ,273 | ,553 |
|  | Physical_exercise | -,039 | ,017 | -,360 | -2,342 | ,024 | -,073 | -,005 |
|  | Sexual_abuse | ,000 | ,005 | ,003 | ,020 | ,984 | -,010 | ,011 |
| a. Dependent Variable: LDL_C_HDL_C_ratio | | | | | | | | |

| **Model Summary** | | | | | | | | | |
| --- | --- | --- | --- | --- | --- | --- | --- | --- | --- |
| Model | R | R Square | Adjusted R Square | Std. Error of the Estimate | Change Statistics | | | | |
|  |  |  |  |  | R Square Change | F Change | df1 | df2 | Sig. F Change |
| 1 | ,355^a^ | ,126 | ,104 | ,10403 | ,126 | 5,770 | 1 | 40 | ,021 |
| 2 | ,537^b^ | ,289 | ,252 | ,09504 | ,163 | 8,920 | 1 | 39 | ,005 |
| a. Predictors: (Constant), Physical_exercise | | | | | | | | | |
| b. Predictors: (Constant), Physical_exercise, Physical_neglect | | | | | | | | | |

| **Coefficients^a^** | | | | | | | | |
| --- | --- | --- | --- | --- | --- | --- | --- | --- |
| Model | | Unstandardized Coefficients | | Standardized Coefficients | t | Sig. | 95,0% Confidence Interval for B | |
|  |  | B | Std. Error | Beta |  |  | Lower Bound | Upper Bound |
| 1 | (Constant) | ,620 | ,048 |  | 12,823 | ,000 | ,522 | ,718 |
|  | Physical_exercise | -,029 | ,012 | -,355 | -2,402 | ,021 | -,054 | -,005 |
| 2 | (Constant) | ,508 | ,058 |  | 8,777 | ,000 | ,391 | ,625 |
|  | Physical_exercise | -,030 | ,011 | -,364 | -2,696 | ,010 | -,052 | -,007 |
|  | Physical_neglect | ,012 | ,004 | ,403 | 2,987 | ,005 | ,004 | ,020 |
| a. Dependent Variable: TC_HDL_C_ratio | | | | | | | | |

| **Model Summary** | | | | | | | | | |
| --- | --- | --- | --- | --- | --- | --- | --- | --- | --- |
| Model | R | R Square | Adjusted R Square | Std. Error of the Estimate | Change Statistics | | | | |
|  |  |  |  |  | R Square Change | F Change | df1 | df2 | Sig. F Change |
| 1 | ,355^a^ | ,126 | ,104 | ,10403 | ,126 | 5,770 | 1 | 40 | ,021 |
| 2 | ,406^b^ | ,165 | ,122 | ,10300 | ,039 | 1,802 | 1 | 39 | ,187 |
| a. Predictors: (Constant), Physical_exercise | | | | | | | | | |
| b. Predictors: (Constant), Physical_exercise, Physical_abuse | | | | | | | | | |

| **Coefficients^a^** | | | | | | | | |
| --- | --- | --- | --- | --- | --- | --- | --- | --- |
| Model | | Unstandardized Coefficients | | Standardized Coefficients | t | Sig. | 95,0% Confidence Interval for B | |
|  |  | B | Std. Error | Beta |  |  | Lower Bound | Upper Bound |
| 1 | (Constant) | ,620 | ,048 |  | 12,823 | ,000 | ,522 | ,718 |
|  | Physical_exercise | -,029 | ,012 | -,355 | -2,402 | ,021 | -,054 | -,005 |
| 2 | (Constant) | ,576 | ,058 |  | 9,929 | ,000 | ,459 | ,693 |
|  | Physical_exercise | -,030 | ,012 | -,371 | -2,526 | ,016 | -,055 | -,006 |
|  | Physical_abuse | ,006 | ,004 | ,197 | 1,342 | ,187 | -,003 | ,015 |
| a. Dependent Variable: TC_HDL_C_ratio | | | | | | | | |

| **Model Summary** | | | | | | | | | |
| --- | --- | --- | --- | --- | --- | --- | --- | --- | --- |
| Model | R | R Square | Adjusted R Square | Std. Error of the Estimate | Change Statistics | | | | |
|  |  |  |  |  | R Square Change | F Change | df1 | df2 | Sig. F Change |
| 1 | ,355^a^ | ,126 | ,104 | ,10403 | ,126 | 5,770 | 1 | 40 | ,021 |
| 2 | ,547^b^ | ,299 | ,263 | ,09436 | ,173 | 9,623 | 1 | 39 | ,004 |
| a. Predictors: (Constant), Physical_exercise | | | | | | | | | |
| b. Predictors: (Constant), Physical_exercise, Emotional_neglect | | | | | | | | | |

| **Coefficients^a^** | | | | | | | | |
| --- | --- | --- | --- | --- | --- | --- | --- | --- |
| Model | | Unstandardized Coefficients | | Standardized Coefficients | t | Sig. | 95,0% Confidence Interval for B | |
|  |  | B | Std. Error | Beta |  |  | Lower Bound | Upper Bound |
| 1 | (Constant) | ,620 | ,048 |  | 12,823 | ,000 | ,522 | ,718 |
|  | Physical_exercise | -,029 | ,012 | -,355 | -2,402 | ,021 | -,054 | -,005 |
| 2 | (Constant) | ,493 | ,060 |  | 8,228 | ,000 | ,372 | ,614 |
|  | Physical_exercise | -,034 | ,011 | -,410 | -3,030 | ,004 | -,056 | -,011 |
|  | Emotional_neglect | ,009 | ,003 | ,419 | 3,102 | ,004 | ,003 | ,015 |
| a. Dependent Variable: TC_HDL_C_ratio | | | | | | | | |

| **Model Summary** | | | | | | | | | |
| --- | --- | --- | --- | --- | --- | --- | --- | --- | --- |
| Model | R | R Square | Adjusted R Square | Std. Error of the Estimate | Change Statistics | | | | |
|  |  |  |  |  | R Square Change | F Change | df1 | df2 | Sig. F Change |
| 1 | ,355^a^ | ,126 | ,104 | ,10403 | ,126 | 5,770 | 1 | 40 | ,021 |
| 2 | ,431^b^ | ,186 | ,144 | ,10169 | ,060 | 2,859 | 1 | 39 | ,099 |
| a. Predictors: (Constant), Physical_exercise | | | | | | | | | |
| b. Predictors: (Constant), Physical_exercise, Emotional_abuse | | | | | | | | | |

| **Coefficients^a^** | | | | | | | | |
| --- | --- | --- | --- | --- | --- | --- | --- | --- |
| Model | | Unstandardized Coefficients | | Standardized Coefficients | t | Sig. | 95,0% Confidence Interval for B | |
|  |  | B | Std. Error | Beta |  |  | Lower Bound | Upper Bound |
| 1 | (Constant) | ,620 | ,048 |  | 12,823 | ,000 | ,522 | ,718 |
|  | Physical_exercise | -,029 | ,012 | -,355 | -2,402 | ,021 | -,054 | -,005 |
| 2 | (Constant) | ,568 | ,056 |  | 10,062 | ,000 | ,454 | ,682 |
|  | Physical_exercise | -,033 | ,012 | -,398 | -2,713 | ,010 | -,057 | -,008 |
|  | Emotional_abuse | ,005 | ,003 | ,248 | 1,691 | ,099 | -,001 | ,010 |
| a. Dependent Variable: TC_HDL_C_ratio | | | | | | | | |

| **Model Summary** | | | | | | | | | |
| --- | --- | --- | --- | --- | --- | --- | --- | --- | --- |
| Model | R | R Square | Adjusted R Square | Std. Error of the Estimate | Change Statistics | | | | |
|  |  |  |  |  | R Square Change | F Change | df1 | df2 | Sig. F Change |
| 1 | ,355^a^ | ,126 | ,104 | ,10403 | ,126 | 5,770 | 1 | 40 | ,021 |
| 2 | ,358^b^ | ,128 | ,083 | ,10524 | ,002 | ,085 | 1 | 39 | ,772 |
| a. Predictors: (Constant), Physical_exercise | | | | | | | | | |
| b. Predictors: (Constant), Physical_exercise, Sexual_abuse | | | | | | | | | |

| **Coefficients^a^** | | | | | | | | |
| --- | --- | --- | --- | --- | --- | --- | --- | --- |
| Model | | Unstandardized Coefficients | | Standardized Coefficients | t | Sig. | 95,0% Confidence Interval for B | |
|  |  | B | Std. Error | Beta |  |  | Lower Bound | Upper Bound |
| 1 | (Constant) | ,620 | ,048 |  | 12,823 | ,000 | ,522 | ,718 |
|  | Physical_exercise | -,029 | ,012 | -,355 | -2,402 | ,021 | -,054 | -,005 |
| 2 | (Constant) | ,615 | ,052 |  | 11,732 | ,000 | ,509 | ,720 |
|  | Physical_exercise | -,030 | ,013 | -,365 | -2,377 | ,022 | -,055 | -,004 |
|  | Sexual_abuse | ,001 | ,004 | ,045 | ,292 | ,772 | -,007 | ,009 |
| Dependent Variable: TC_HDL_C_ratio | | | | | | | | |

|  | **HC**  **(n=20)** | **entire MDD**  **(n=42)** | **MDD Only**  **(n=21)** | **MDD**  **+ELS**  **(n=21)** | **Statistics of the comparisons between HC and overall MDD** | **Statistics of the comparisons between HC, MDD Only, and MDD+ELS** |
| --- | --- | --- | --- | --- | --- | --- |
| **CPT omissions^a^** | 1.00  (1.00) | 2.00  (4.25) | 2.00  (4.00) | 3.00  (5.00) | Welch’s *F*_(1,58.5)_*=*7.464, *P*=0.008 | Welch’s *F*_(2,36.6)_*=*3.780,  *P=*0.032  HC<MDD+ELS, *P*=0.045 |
| **CPT commissions^b^** | 12.05  (5.17) | 14.05  (7.22) | 12.10  (5.64) | 16.00  (8.19) | *F*_(1,60)_*=*1.226, *P=*0.273 | Welch’s *F*_(2,38.5)_*=*1.943, *P=*0.157 |
| **CPT hit reaction time (msec)^a^** | 361.80  (40.47) | 386.13  (72.14) | 376.50  (55.40) | 388.30  (80.37) | *F*_(1,60)_*=*3.432, *P=*0.069 | *F*_(2,59)_*=*2.039, *P*=0.139 |
| **CPT detectability^b^** | 0.74  (0.36) | 0.69  (0.42) | 0.73  (0.42) | 0.60  (0.47) | *F*_(1,60)_*=*0.454, *P=*0.503 | *F*_(2,59)_*=*0.769, *P*=0.468 |
| **WCST total correct**  **responses^a^** | 71.00  (12.25) | 70.00  (14.75) | 72.00  (18.00) | 67.00  (13.50) | *F*_(1,60)_*=*0.289, *P=*0.593 | *F*_(2,59)_*=*0.681, *P*=0.510 |
| **WCST perseverative**  **Errors^a^** | 7.00  (5.5) | 11.00  (21.00) | 10.00  (18.50) | 17.00  (26.00) | Welch’s *F*_(1,50.8)_*=*5.463, *P*=0.023 | *F*_(2,59)_*=*2.617, *P*=0.082 |
| **WCST non-perseverative**  **errors^a^** | 8  (7.00) | 12.00  (17.25) | 15.00  (17.50) | 12.00  (16.00) | *F*_(1,60)_*=*3.479, *P=*0.067 | *F*_(2,59)_*=*1.835, *P*=0.169 |
| **WCST conceptual level**  **responses^b^** | 66.80  (7.05) | 62.33  (13.37) | 64.62  (14.60) | 60.05  (11.94) | *F*_(1,60)_*=*1.960, *P=*0.167 | *F*_(2,59)_*=*1.794, *P*=0.175 |

## Supplementary Table 4.

## Neurocognitive test results, and one-way ANOVA results of the comparisons between healthy controls and MDD groups in neurocognitive performances

^a^ Medians and inter-quartile ranges are presented. ^b^ Means and standard deviations are presented.

CPT, Conners’ Continuous Performance Test II; MDD, major depressive disorder; ELS, early life stress; WCST, Wisconsin Card Sorting Test

## Supplementary Table 5.

## One-way ANCOVA results of the neurocognitive performances

|  | **Statistics of the comparisons between HC and overall MDD** | **Statistics of the comparisons between HC, MDD Only, and MDD+ELS** |
| --- | --- | --- |
| **CPT omissions** | *F*_(1,57)_*=*1.391, *P*=0.243 | *F*_(2,56)_*=*0.897, *P=*0.414 |
| **CPT commissions** | *F*_(1,57)_*=*0.001, *P=*0.971 | *F*_(2,56)_*=*1.329, *P=*0.273 |
| **CPT hit reaction time (msec)** | *F*_(1,57)_*=*1.522, *P=*0.222 | *F*_(2,56)_*=*1.206, *P*=0.307 |
| **CPT detectability** | *F*_(1,57)_*=*0.102, *P=*0.750 | *F*_(2,56)_*=*0.198, *P*=0.821 |
| **WCST total correct**  **responses** | *F*_(1,57)_*=*1.720, *P=*0.195 | *F*_(2,56)_*=*1.495, *P*=0.233 |
| **WCST perseverative**  **errors** | *F*_(1,57)_*=*0.136, *P*=0.714 | *F*_(2,56)_*=*0.207, *P*=0.813 |
| **WCST non-perseverative**  **errors** | *F*_(1,57)_*=*0.042, *P=*0.838 | *F*_(2,56)_*=*0.380, *P*=0.685 |
| **WCST conceptual level**  **responses** | *F*_(1,57)_*=*1.777, *P=*0.188 | *F*_(2,56)_*=*1.487, *P*=0.235 |

## Supplementary Table 6.

## Hierarchical linear regression analyses of serum lipid and lipoprotein levels as predictors of attentional performances (CPT scores) in the entire MDD group

| **Model Summary** | | | | | | | | | |
| --- | --- | --- | --- | --- | --- | --- | --- | --- | --- |
| Model | R | R Square | Adjusted R Square | Std. Error of the Estimate | Change Statistics | | | | |
|  |  |  |  |  | R Square Change | F Change | df1 | df2 | Sig. F Change |
| 1 | ,088^a^ | ,008 | -,017 | ,46060 | ,008 | ,314 | 1 | 40 | ,578 |
| a. Predictors: (Constant), Total_cholesterol | | | | | | | | | |

| **Coefficients^a^** | | | | | | | | |
| --- | --- | --- | --- | --- | --- | --- | --- | --- |
| Model | | Unstandardized Coefficients | | Standardized Coefficients | t | Sig. | 95,0% Confidence Interval for B | |
|  |  | B | Std. Error | Beta |  |  | Lower Bound | Upper Bound |
| 1 | (Constant) | ,331 | ,347 |  | ,954 | ,346 | -,370 | 1,032 |
|  | Total_cholesterol | ,037 | ,067 | ,088 | ,560 | ,578 | -,097 | ,172 |
| a. Dependent Variable: CPT_OM | | | | | | | | |

| **Model Summary** | | | | | | | | | |
| --- | --- | --- | --- | --- | --- | --- | --- | --- | --- |
| Model | R | R Square | Adjusted R Square | Std. Error of the Estimate | Change Statistics | | | | |
|  |  |  |  |  | R Square Change | F Change | df1 | df2 | Sig. F Change |
| 1 | ,113^a^ | ,013 | -,012 | ,45943 | ,013 | ,520 | 1 | 40 | ,475 |
| a. Predictors: (Constant), Triglycerides | | | | | | | | | |

| **Coefficients^a^** | | | | | | | | |
| --- | --- | --- | --- | --- | --- | --- | --- | --- |
| Model | | Unstandardized Coefficients | | Standardized Coefficients | t | Sig. | 95,0% Confidence Interval for B | |
|  |  | B | Std. Error | Beta |  |  | Lower Bound | Upper Bound |
| 1 | (Constant) | ,506 | ,074 |  | 6,863 | ,000 | ,357 | ,655 |
|  | Triglycerides | ,273 | ,379 | ,113 | ,721 | ,475 | -,493 | 1,039 |
| a. Dependent Variable: CPT_OM | | | | | | | | |

| **Model Summary** | | | | | | | | | |
| --- | --- | --- | --- | --- | --- | --- | --- | --- | --- |
| Model | R | R Square | Adjusted R Square | Std. Error of the Estimate | Change Statistics | | | | |
|  |  |  |  |  | R Square Change | F Change | df1 | df2 | Sig. F Change |
| 1 | ,022^a^ | ,000 | -,024 | ,46229 | ,000 | ,020 | 1 | 40 | ,889 |
| a. Predictors: (Constant), HDL_cholesterol | | | | | | | | | |

| **Coefficients^a^** | | | | | | | | |
| --- | --- | --- | --- | --- | --- | --- | --- | --- |
| Model | | Unstandardized Coefficients | | Standardized Coefficients | t | Sig. | 95,0% Confidence Interval for B | |
|  |  | B | Std. Error | Beta |  |  | Lower Bound | Upper Bound |
| 1 | (Constant) | ,565 | ,319 |  | 1,769 | ,085 | -,081 | 1,210 |
|  | HDL_cholesterol | -,028 | ,197 | -,022 | -,141 | ,889 | -,426 | ,371 |
| a. Dependent Variable: CPT_OM | | | | | | | | |

| **Model Summary** | | | | | | | | | |
| --- | --- | --- | --- | --- | --- | --- | --- | --- | --- |
| Model | R | R Square | Adjusted R Square | Std. Error of the Estimate | Change Statistics | | | | |
|  |  |  |  |  | R Square Change | F Change | df1 | df2 | Sig. F Change |
| 1 | ,115^a^ | ,013 | -,011 | ,45931 | ,013 | ,540 | 1 | 40 | ,467 |
| a. Predictors: (Constant), LDL_cholesterol | | | | | | | | | |

| **Coefficients^a^** | | | | | | | | |
| --- | --- | --- | --- | --- | --- | --- | --- | --- |
| Model | | Unstandardized Coefficients | | Standardized Coefficients | t | Sig. | 95,0% Confidence Interval for B | |
|  |  | B | Std. Error | Beta |  |  | Lower Bound | Upper Bound |
| 1 | (Constant) | ,325 | ,276 |  | 1,178 | ,246 | -,233 | ,883 |
|  | LDL_cholesterol | ,066 | ,090 | ,115 | ,735 | ,467 | -,116 | ,249 |
| a. Dependent Variable: CPT_OM | | | | | | | | |

| **Model Summary** | | | | | | | | | |
| --- | --- | --- | --- | --- | --- | --- | --- | --- | --- |
| Model | R | R Square | Adjusted R Square | Std. Error of the Estimate | Change Statistics | | | | |
|  |  |  |  |  | R Square Change | F Change | df1 | df2 | Sig. F Change |
| 1 | ,106^a^ | ,011 | -,013 | ,45980 | ,011 | ,455 | 1 | 40 | ,504 |
| a. Predictors: (Constant), LDL_C_HDL_C_ratio | | | | | | | | | |

| **Coefficients^a^** | | | | | | | | |
| --- | --- | --- | --- | --- | --- | --- | --- | --- |
| Model | | Unstandardized Coefficients | | Standardized Coefficients | t | Sig. | 95,0% Confidence Interval for B | |
|  |  | B | Std. Error | Beta |  |  | Lower Bound | Upper Bound |
| 1 | (Constant) | ,432 | ,150 |  | 2,874 | ,006 | ,128 | ,735 |
|  | LDL_C_HDL_C_ratio | ,334 | ,495 | ,106 | ,674 | ,504 | -,667 | 1,334 |
| a. Dependent Variable: CPT_OM | | | | | | | | |

| **Model Summary** | | | | | | | | | |
| --- | --- | --- | --- | --- | --- | --- | --- | --- | --- |
| Model | R | R Square | Adjusted R Square | Std. Error of the Estimate | Change Statistics | | | | |
|  |  |  |  |  | R Square Change | F Change | df1 | df2 | Sig. F Change |
| 1 | ,081^a^ | ,007 | -,018 | ,46089 | ,007 | ,263 | 1 | 40 | ,611 |
| a. Predictors: (Constant), TC_HDL_C_ratio | | | | | | | | | |

| **Coefficients^a^** | | | | | | | | |
| --- | --- | --- | --- | --- | --- | --- | --- | --- |
| Model | | Unstandardized Coefficients | | Standardized Coefficients | t | Sig. | 95,0% Confidence Interval for B | |
|  |  | B | Std. Error | Beta |  |  | Lower Bound | Upper Bound |
| 1 | (Constant) | ,350 | ,342 |  | 1,023 | ,312 | -,341 | 1,040 |
|  | TC_HDL_C_ratio | ,336 | ,655 | ,081 | ,512 | ,611 | -,988 | 1,659 |
| a. Dependent Variable: CPT_OM | | | | | | | | |

| **Model Summary** | | | | | | | | | |
| --- | --- | --- | --- | --- | --- | --- | --- | --- | --- |
| Model | R | R Square | Adjusted R Square | Std. Error of the Estimate | Change Statistics | | | | |
|  |  |  |  |  | R Square Change | F Change | df1 | df2 | Sig. F Change |
| 1 | ,360^a^ | ,130 | ,108 | 6,81957 | ,130 | 5,970 | 1 | 40 | ,019 |
| 2 | ,544^b^ | ,295 | ,259 | 6,21470 | ,166 | 9,165 | 1 | 39 | ,004 |
| 3 | ,632^c^ | ,399 | ,352 | 5,81427 | ,104 | 6,557 | 1 | 38 | ,015 |
| 4 | ,691^d^ | ,478 | ,421 | 5,49366 | ,079 | 5,565 | 1 | 37 | ,024 |
| 5 | ,702^e^ | ,493 | ,422 | 5,48831 | ,015 | 1,072 | 1 | 36 | ,307 |
| a. Predictors: (Constant), Age | | | | | | | | | |
| b. Predictors: (Constant), Age, Gender | | | | | | | | | |
| c. Predictors: (Constant), Age, Gender, Education | | | | | | | | | |
| d. Predictors: (Constant), Age, Gender, Education, Depression_severity | | | | | | | | | |
| e. Predictors: (Constant), Age, Gender, Education, Depression_severity, Total_cholesterol | | | | | | | | | |

| **Coefficients^a^** | | | | | | | | |
| --- | --- | --- | --- | --- | --- | --- | --- | --- |
| Model | | Unstandardized Coefficients | | Standardized Coefficients | t | Sig. | 95,0% Confidence Interval for B | |
|  |  | B | Std. Error | Beta |  |  | Lower Bound | Upper Bound |
| 1 | (Constant) | 4,579 | 4,015 |  | 1,140 | ,261 | -3,536 | 12,695 |
|  | Age | ,267 | ,109 | ,360 | 2,443 | ,019 | ,046 | ,489 |
| 2 | (Constant) | 8,531 | 3,885 |  | 2,196 | ,034 | ,673 | 16,390 |
|  | Age | ,315 | ,101 | ,424 | 3,117 | ,003 | ,111 | ,519 |
|  | Gender | -7,162 | 2,366 | -,412 | -3,027 | ,004 | -11,946 | -2,377 |
| 3 | (Constant) | 18,756 | 5,400 |  | 3,474 | ,001 | 7,825 | 29,687 |
|  | Age | ,347 | ,095 | ,467 | 3,639 | ,001 | ,154 | ,540 |
|  | Gender | -7,427 | 2,216 | -,427 | -3,352 | ,002 | -11,912 | -2,941 |
|  | Education | -,922 | ,360 | -,325 | -2,561 | ,015 | -1,651 | -,193 |
| 4 | (Constant) | 12,677 | 5,716 |  | 2,218 | ,033 | 1,096 | 24,258 |
|  | Age | ,331 | ,090 | ,446 | 3,669 | ,001 | ,148 | ,514 |
|  | Gender | -7,793 | 2,099 | -,448 | -3,712 | ,001 | -12,046 | -3,540 |
|  | Education | -,736 | ,349 | -,259 | -2,107 | ,042 | -1,443 | -,028 |
|  | Depression_severity | ,201 | ,085 | ,289 | 2,359 | ,024 | ,028 | ,374 |
| 5 | (Constant) | 16,166 | 6,630 |  | 2,438 | ,020 | 2,719 | 29,613 |
|  | Age | ,372 | ,098 | ,502 | 3,779 | ,001 | ,172 | ,572 |
|  | Gender | -7,903 | 2,100 | -,455 | -3,764 | ,001 | -12,162 | -3,645 |
|  | Education | -,743 | ,349 | -,262 | -2,129 | ,040 | -1,450 | -,035 |
|  | Depression_severity | ,193 | ,086 | ,278 | 2,261 | ,030 | ,020 | ,367 |
|  | Total_cholesterol | -,900 | ,869 | -,135 | -1,035 | ,307 | -2,663 | ,863 |
| a. Dependent Variable: CPT_Com | | | | | | | | |

| **Model Summary** | | | | | | | | | |
| --- | --- | --- | --- | --- | --- | --- | --- | --- | --- |
| Model | R | R Square | Adjusted R Square | Std. Error of the Estimate | Change Statistics | | | | |
|  |  |  |  |  | R Square Change | F Change | df1 | df2 | Sig. F Change |
| 1 | ,360^a^ | ,130 | ,108 | 6,81957 | ,130 | 5,970 | 1 | 40 | ,019 |
| 2 | ,544^b^ | ,295 | ,259 | 6,21470 | ,166 | 9,165 | 1 | 39 | ,004 |
| 3 | ,632^c^ | ,399 | ,352 | 5,81427 | ,104 | 6,557 | 1 | 38 | ,015 |
| 4 | ,691^d^ | ,478 | ,421 | 5,49366 | ,079 | 5,565 | 1 | 37 | ,024 |
| 5 | ,696^e^ | ,485 | ,413 | 5,53285 | ,007 | ,478 | 1 | 36 | ,494 |
| a. Predictors: (Constant), Age | | | | | | | | | |
| b. Predictors: (Constant), Age, Gender | | | | | | | | | |
| c. Predictors: (Constant), Age, Gender, Education | | | | | | | | | |
| d. Predictors: (Constant), Age, Gender, Education, Depression_severity | | | | | | | | | |
| e. Predictors: (Constant), Age, Gender, Education, Depression_severity, Triglycerides | | | | | | | | | |

| **Coefficients^a^** | | | | | | | | |
| --- | --- | --- | --- | --- | --- | --- | --- | --- |
| Model | | Unstandardized Coefficients | | Standardized Coefficients | t | Sig. | 95,0% Confidence Interval for B | |
|  |  | B | Std. Error | Beta |  |  | Lower Bound | Upper Bound |
| 1 | (Constant) | 4,579 | 4,015 |  | 1,140 | ,261 | -3,536 | 12,695 |
|  | Age | ,267 | ,109 | ,360 | 2,443 | ,019 | ,046 | ,489 |
| 2 | (Constant) | 8,531 | 3,885 |  | 2,196 | ,034 | ,673 | 16,390 |
|  | Age | ,315 | ,101 | ,424 | 3,117 | ,003 | ,111 | ,519 |
|  | Gender | -7,162 | 2,366 | -,412 | -3,027 | ,004 | -11,946 | -2,377 |
| 3 | (Constant) | 18,756 | 5,400 |  | 3,474 | ,001 | 7,825 | 29,687 |
|  | Age | ,347 | ,095 | ,467 | 3,639 | ,001 | ,154 | ,540 |
|  | Gender | -7,427 | 2,216 | -,427 | -3,352 | ,002 | -11,912 | -2,941 |
|  | Education | -,922 | ,360 | -,325 | -2,561 | ,015 | -1,651 | -,193 |
| 4 | (Constant) | 12,677 | 5,716 |  | 2,218 | ,033 | 1,096 | 24,258 |
|  | Age | ,331 | ,090 | ,446 | 3,669 | ,001 | ,148 | ,514 |
|  | Gender | -7,793 | 2,099 | -,448 | -3,712 | ,001 | -12,046 | -3,540 |
|  | Education | -,736 | ,349 | -,259 | -2,107 | ,042 | -1,443 | -,028 |
|  | Depression_severity | ,201 | ,085 | ,289 | 2,359 | ,024 | ,028 | ,374 |
| 5 | (Constant) | 13,409 | 5,853 |  | 2,291 | ,028 | 1,538 | 25,280 |
|  | Age | ,311 | ,095 | ,419 | 3,261 | ,002 | ,118 | ,505 |
|  | Gender | -7,477 | 2,163 | -,430 | -3,456 | ,001 | -11,864 | -3,090 |
|  | Education | -,742 | ,352 | -,261 | -2,109 | ,042 | -1,455 | -,028 |
|  | Depression_severity | ,184 | ,089 | ,265 | 2,067 | ,046 | ,003 | ,365 |
|  | Triglycerides | 3,478 | 5,033 | ,091 | ,691 | ,494 | -6,729 | 13,686 |
| a. Dependent Variable: CPT_Com | | | | | | | | |

| **Model Summary** | | | | | | | | | |
| --- | --- | --- | --- | --- | --- | --- | --- | --- | --- |
| Model | R | R Square | Adjusted R Square | Std. Error of the Estimate | Change Statistics | | | | |
|  |  |  |  |  | R Square Change | F Change | df1 | df2 | Sig. F Change |
| 1 | ,360^a^ | ,130 | ,108 | 6,81957 | ,130 | 5,970 | 1 | 40 | ,019 |
| 2 | ,544^b^ | ,295 | ,259 | 6,21470 | ,166 | 9,165 | 1 | 39 | ,004 |
| 3 | ,632^c^ | ,399 | ,352 | 5,81427 | ,104 | 6,557 | 1 | 38 | ,015 |
| 4 | ,691^d^ | ,478 | ,421 | 5,49366 | ,079 | 5,565 | 1 | 37 | ,024 |
| 5 | ,692^e^ | ,479 | ,407 | 5,56170 | ,001 | ,100 | 1 | 36 | ,753 |
| a. Predictors: (Constant), Age | | | | | | | | | |
| b. Predictors: (Constant), Age, Gender | | | | | | | | | |
| c. Predictors: (Constant), Age, Gender, Education | | | | | | | | | |
| d. Predictors: (Constant), Age, Gender, Education, Depression_severity | | | | | | | | | |
| e. Predictors: (Constant), Age, Gender, Education, Depression_severity, HDL_cholesterol | | | | | | | | | |

| **Coefficients^a^** | | | | | | | | |
| --- | --- | --- | --- | --- | --- | --- | --- | --- |
| Model | | Unstandardized Coefficients | | Standardized Coefficients | t | Sig. | 95,0% Confidence Interval for B | |
|  |  | B | Std. Error | Beta |  |  | Lower Bound | Upper Bound |
| 1 | (Constant) | 4,579 | 4,015 |  | 1,140 | ,261 | -3,536 | 12,695 |
|  | Age | ,267 | ,109 | ,360 | 2,443 | ,019 | ,046 | ,489 |
| 2 | (Constant) | 8,531 | 3,885 |  | 2,196 | ,034 | ,673 | 16,390 |
|  | Age | ,315 | ,101 | ,424 | 3,117 | ,003 | ,111 | ,519 |
|  | Gender | -7,162 | 2,366 | -,412 | -3,027 | ,004 | -11,946 | -2,377 |
| 3 | (Constant) | 18,756 | 5,400 |  | 3,474 | ,001 | 7,825 | 29,687 |
|  | Age | ,347 | ,095 | ,467 | 3,639 | ,001 | ,154 | ,540 |
|  | Gender | -7,427 | 2,216 | -,427 | -3,352 | ,002 | -11,912 | -2,941 |
|  | Education | -,922 | ,360 | -,325 | -2,561 | ,015 | -1,651 | -,193 |
| 4 | (Constant) | 12,677 | 5,716 |  | 2,218 | ,033 | 1,096 | 24,258 |
|  | Age | ,331 | ,090 | ,446 | 3,669 | ,001 | ,148 | ,514 |
|  | Gender | -7,793 | 2,099 | -,448 | -3,712 | ,001 | -12,046 | -3,540 |
|  | Education | -,736 | ,349 | -,259 | -2,107 | ,042 | -1,443 | -,028 |
|  | Depression_severity | ,201 | ,085 | ,289 | 2,359 | ,024 | ,028 | ,374 |
| 5 | (Constant) | 14,000 | 7,137 |  | 1,962 | ,058 | -,474 | 28,473 |
|  | Age | ,337 | ,093 | ,454 | 3,618 | ,001 | ,148 | ,525 |
|  | Gender | -7,672 | 2,159 | -,441 | -3,553 | ,001 | -12,051 | -3,292 |
|  | Education | -,734 | ,354 | -,259 | -2,077 | ,045 | -1,451 | -,017 |
|  | Depression_severity | ,189 | ,095 | ,271 | 1,987 | ,055 | -,004 | ,381 |
|  | HDL_cholesterol | -,851 | 2,687 | -,043 | -,317 | ,753 | -6,300 | 4,598 |
| a. Dependent Variable: CPT_Com | | | | | | | | |

| **Model Summary** | | | | | | | | | |
| --- | --- | --- | --- | --- | --- | --- | --- | --- | --- |
| Model | R | R Square | Adjusted R Square | Std. Error of the Estimate | Change Statistics | | | | |
|  |  |  |  |  | R Square Change | F Change | df1 | df2 | Sig. F Change |
| 1 | ,360^a^ | ,130 | ,108 | 6,81957 | ,130 | 5,970 | 1 | 40 | ,019 |
| 2 | ,544^b^ | ,295 | ,259 | 6,21470 | ,166 | 9,165 | 1 | 39 | ,004 |
| 3 | ,632^c^ | ,399 | ,352 | 5,81427 | ,104 | 6,557 | 1 | 38 | ,015 |
| 4 | ,691^d^ | ,478 | ,421 | 5,49366 | ,079 | 5,565 | 1 | 37 | ,024 |
| 5 | ,702^e^ | ,493 | ,422 | 5,48773 | ,015 | 1,080 | 1 | 36 | ,306 |
| a. Predictors: (Constant), Age | | | | | | | | | |
| b. Predictors: (Constant), Age, Gender | | | | | | | | | |
| c. Predictors: (Constant), Age, Gender, Education | | | | | | | | | |
| d. Predictors: (Constant), Age, Gender, Education, Depression_severity | | | | | | | | | |
| e. Predictors: (Constant), Age, Gender, Education, Depression_severity, LDL_cholesterol | | | | | | | | | |

| **Coefficients^a^** | | | | | | | | |
| --- | --- | --- | --- | --- | --- | --- | --- | --- |
| Model | | Unstandardized Coefficients | | Standardized Coefficients | t | Sig. | 95,0% Confidence Interval for B | |
|  |  | B | Std. Error | Beta |  |  | Lower Bound | Upper Bound |
| 1 | (Constant) | 4,579 | 4,015 |  | 1,140 | ,261 | -3,536 | 12,695 |
|  | Age | ,267 | ,109 | ,360 | 2,443 | ,019 | ,046 | ,489 |
| 2 | (Constant) | 8,531 | 3,885 |  | 2,196 | ,034 | ,673 | 16,390 |
|  | Age | ,315 | ,101 | ,424 | 3,117 | ,003 | ,111 | ,519 |
|  | Gender | -7,162 | 2,366 | -,412 | -3,027 | ,004 | -11,946 | -2,377 |
| 3 | (Constant) | 18,756 | 5,400 |  | 3,474 | ,001 | 7,825 | 29,687 |
|  | Age | ,347 | ,095 | ,467 | 3,639 | ,001 | ,154 | ,540 |
|  | Gender | -7,427 | 2,216 | -,427 | -3,352 | ,002 | -11,912 | -2,941 |
|  | Education | -,922 | ,360 | -,325 | -2,561 | ,015 | -1,651 | -,193 |
| 4 | (Constant) | 12,677 | 5,716 |  | 2,218 | ,033 | 1,096 | 24,258 |
|  | Age | ,331 | ,090 | ,446 | 3,669 | ,001 | ,148 | ,514 |
|  | Gender | -7,793 | 2,099 | -,448 | -3,712 | ,001 | -12,046 | -3,540 |
|  | Education | -,736 | ,349 | -,259 | -2,107 | ,042 | -1,443 | -,028 |
|  | Depression_severity | ,201 | ,085 | ,289 | 2,359 | ,024 | ,028 | ,374 |
| 5 | (Constant) | 15,075 | 6,158 |  | 2,448 | ,019 | 2,585 | 27,565 |
|  | Age | ,372 | ,098 | ,501 | 3,784 | ,001 | ,172 | ,571 |
|  | Gender | -8,011 | 2,107 | -,461 | -3,801 | ,001 | -12,285 | -3,737 |
|  | Education | -,739 | ,349 | -,261 | -2,119 | ,041 | -1,446 | -,032 |
|  | Depression_severity | ,201 | ,085 | ,289 | 2,359 | ,024 | ,028 | ,374 |
|  | LDL_cholesterol | -1,223 | 1,177 | -,135 | -1,039 | ,306 | -3,610 | 1,164 |
| a. Dependent Variable: CPT_Com | | | | | | | | |

| **Model Summary** | | | | | | | | | |
| --- | --- | --- | --- | --- | --- | --- | --- | --- | --- |
| Model | R | R Square | Adjusted R Square | Std. Error of the Estimate | Change Statistics | | | | |
|  |  |  |  |  | R Square Change | F Change | df1 | df2 | Sig. F Change |
| 1 | ,360^a^ | ,130 | ,108 | 6,81957 | ,130 | 5,970 | 1 | 40 | ,019 |
| 2 | ,544^b^ | ,295 | ,259 | 6,21470 | ,166 | 9,165 | 1 | 39 | ,004 |
| 3 | ,632^c^ | ,399 | ,352 | 5,81427 | ,104 | 6,557 | 1 | 38 | ,015 |
| 4 | ,691^d^ | ,478 | ,421 | 5,49366 | ,079 | 5,565 | 1 | 37 | ,024 |
| 5 | ,695^e^ | ,482 | ,411 | 5,54407 | ,005 | ,330 | 1 | 36 | ,569 |
| a. Predictors: (Constant), Age | | | | | | | | | |
| b. Predictors: (Constant), Age, Gender | | | | | | | | | |
| c. Predictors: (Constant), Age, Gender, Education | | | | | | | | | |
| d. Predictors: (Constant), Age, Gender, Education, Depression_severity | | | | | | | | | |
| e. Predictors: (Constant), Age, Gender, Education, Depression_severity, LDL_C_HDL_C_ratio | | | | | | | | | |

| **Coefficients^a^** | | | | | | | | |
| --- | --- | --- | --- | --- | --- | --- | --- | --- |
| Model | | Unstandardized Coefficients | | Standardized Coefficients | t | Sig. | 95,0% Confidence Interval for B | |
|  |  | B | Std. Error | Beta |  |  | Lower Bound | Upper Bound |
| 1 | (Constant) | 4,579 | 4,015 |  | 1,140 | ,261 | -3,536 | 12,695 |
|  | Age | ,267 | ,109 | ,360 | 2,443 | ,019 | ,046 | ,489 |
| 2 | (Constant) | 8,531 | 3,885 |  | 2,196 | ,034 | ,673 | 16,390 |
|  | Age | ,315 | ,101 | ,424 | 3,117 | ,003 | ,111 | ,519 |
|  | Gender | -7,162 | 2,366 | -,412 | -3,027 | ,004 | -11,946 | -2,377 |
| 3 | (Constant) | 18,756 | 5,400 |  | 3,474 | ,001 | 7,825 | 29,687 |
|  | Age | ,347 | ,095 | ,467 | 3,639 | ,001 | ,154 | ,540 |
|  | Gender | -7,427 | 2,216 | -,427 | -3,352 | ,002 | -11,912 | -2,941 |
|  | Education | -,922 | ,360 | -,325 | -2,561 | ,015 | -1,651 | -,193 |
| 4 | (Constant) | 12,677 | 5,716 |  | 2,218 | ,033 | 1,096 | 24,258 |
|  | Age | ,331 | ,090 | ,446 | 3,669 | ,001 | ,148 | ,514 |
|  | Gender | -7,793 | 2,099 | -,448 | -3,712 | ,001 | -12,046 | -3,540 |
|  | Education | -,736 | ,349 | -,259 | -2,107 | ,042 | -1,443 | -,028 |
|  | Depression_severity | ,201 | ,085 | ,289 | 2,359 | ,024 | ,028 | ,374 |
| 5 | (Constant) | 13,120 | 5,820 |  | 2,255 | ,030 | 1,318 | 24,923 |
|  | Age | ,342 | ,093 | ,461 | 3,676 | ,001 | ,153 | ,531 |
|  | Gender | -8,020 | 2,155 | -,461 | -3,722 | ,001 | -12,391 | -3,650 |
|  | Education | -,737 | ,352 | -,260 | -2,090 | ,044 | -1,451 | -,022 |
|  | Depression_severity | ,216 | ,090 | ,311 | 2,404 | ,022 | ,034 | ,399 |
|  | LDL_C_HDL_C_ratio | -3,701 | 6,440 | -,074 | -,575 | ,569 | -16,761 | 9,360 |
| a. Dependent Variable: CPT_Com | | | | | | | | |

| **Model Summary** | | | | | | | | | |
| --- | --- | --- | --- | --- | --- | --- | --- | --- | --- |
| Model | R | R Square | Adjusted R Square | Std. Error of the Estimate | Change Statistics | | | | |
|  |  |  |  |  | R Square Change | F Change | df1 | df2 | Sig. F Change |
| 1 | ,360^a^ | ,130 | ,108 | 6,81957 | ,130 | 5,970 | 1 | 40 | ,019 |
| 2 | ,544^b^ | ,295 | ,259 | 6,21470 | ,166 | 9,165 | 1 | 39 | ,004 |
| 3 | ,632^c^ | ,399 | ,352 | 5,81427 | ,104 | 6,557 | 1 | 38 | ,015 |
| 4 | ,691^d^ | ,478 | ,421 | 5,49366 | ,079 | 5,565 | 1 | 37 | ,024 |
| 5 | ,695^e^ | ,483 | ,411 | 5,54233 | ,005 | ,353 | 1 | 36 | ,556 |
| a. Predictors: (Constant), Age | | | | | | | | | |
| b. Predictors: (Constant), Age, Gender | | | | | | | | | |
| c. Predictors: (Constant), Age, Gender, Education | | | | | | | | | |
| d. Predictors: (Constant), Age, Gender, Education, Depression_severity | | | | | | | | | |
| e. Predictors: (Constant), Age, Gender, Education, Depression_severity, TC_HDL_C_ratio | | | | | | | | | |

| **Coefficients^a^** | | | | | | | | |
| --- | --- | --- | --- | --- | --- | --- | --- | --- |
| Model | | Unstandardized Coefficients | | Standardized Coefficients | t | Sig. | 95,0% Confidence Interval for B | |
|  |  | B | Std. Error | Beta |  |  | Lower Bound | Upper Bound |
| 1 | (Constant) | 4,579 | 4,015 |  | 1,140 | ,261 | -3,536 | 12,695 |
|  | Age | ,267 | ,109 | ,360 | 2,443 | ,019 | ,046 | ,489 |
| 2 | (Constant) | 8,531 | 3,885 |  | 2,196 | ,034 | ,673 | 16,390 |
|  | Age | ,315 | ,101 | ,424 | 3,117 | ,003 | ,111 | ,519 |
|  | Gender | -7,162 | 2,366 | -,412 | -3,027 | ,004 | -11,946 | -2,377 |
| 3 | (Constant) | 18,756 | 5,400 |  | 3,474 | ,001 | 7,825 | 29,687 |
|  | Age | ,347 | ,095 | ,467 | 3,639 | ,001 | ,154 | ,540 |
|  | Gender | -7,427 | 2,216 | -,427 | -3,352 | ,002 | -11,912 | -2,941 |
|  | Education | -,922 | ,360 | -,325 | -2,561 | ,015 | -1,651 | -,193 |
| 4 | (Constant) | 12,677 | 5,716 |  | 2,218 | ,033 | 1,096 | 24,258 |
|  | Age | ,331 | ,090 | ,446 | 3,669 | ,001 | ,148 | ,514 |
|  | Gender | -7,793 | 2,099 | -,448 | -3,712 | ,001 | -12,046 | -3,540 |
|  | Education | -,736 | ,349 | -,259 | -2,107 | ,042 | -1,443 | -,028 |
|  | Depression_severity | ,201 | ,085 | ,289 | 2,359 | ,024 | ,028 | ,374 |
| 5 | (Constant) | 14,754 | 6,743 |  | 2,188 | ,035 | 1,078 | 28,430 |
|  | Age | ,342 | ,093 | ,461 | 3,682 | ,001 | ,154 | ,531 |
|  | Gender | -8,038 | 2,157 | -,462 | -3,726 | ,001 | -12,413 | -3,662 |
|  | Education | -,741 | ,352 | -,261 | -2,104 | ,042 | -1,456 | -,027 |
|  | Depression_severity | ,217 | ,090 | ,313 | 2,408 | ,021 | ,034 | ,401 |
|  | TC_HDL_C_ratio | -5,078 | 8,547 | -,077 | -,594 | ,556 | -22,413 | 12,257 |
| a. Dependent Variable: CPT_Com | | | | | | | | |

| **Model Summary** | | | | | | | | | |
| --- | --- | --- | --- | --- | --- | --- | --- | --- | --- |
| Model | R | R Square | Adjusted R Square | Std. Error of the Estimate | Change Statistics | | | | |
|  |  |  |  |  | R Square Change | F Change | df1 | df2 | Sig. F Change |
| 1 | ,401^a^ | ,161 | ,140 | ,05537 | ,161 | 7,666 | 1 | 40 | ,008 |
| 2 | ,414^b^ | ,172 | ,129 | ,05571 | ,011 | ,516 | 1 | 39 | ,477 |
| a. Predictors: (Constant), Gender | | | | | | | | | |
| b. Predictors: (Constant), Gender, Total_cholesterol | | | | | | | | | |

| **Coefficients^a^** | | | | | | | | |
| --- | --- | --- | --- | --- | --- | --- | --- | --- |
| Model | | Unstandardized Coefficients | | Standardized Coefficients | t | Sig. | 95,0% Confidence Interval for B | |
|  |  | B | Std. Error | Beta |  |  | Lower Bound | Upper Bound |
| 1 | (Constant) | 2,554 | ,018 |  | 138,365 | ,000 | 2,517 | 2,591 |
|  | Gender | ,058 | ,021 | ,401 | 2,769 | ,008 | ,016 | ,100 |
| 2 | (Constant) | 2,525 | ,045 |  | 56,196 | ,000 | 2,434 | 2,615 |
|  | Gender | ,058 | ,021 | ,400 | 2,745 | ,009 | ,015 | ,100 |
|  | Total_cholesterol | ,006 | ,008 | ,105 | ,719 | ,477 | -,011 | ,022 |
| a. Dependent Variable: CPT_RT | | | | | | | | |

| **Model Summary** | | | | | | | | | |
| --- | --- | --- | --- | --- | --- | --- | --- | --- | --- |
| Model | R | R Square | Adjusted R Square | Std. Error of the Estimate | Change Statistics | | | | |
|  |  |  |  |  | R Square Change | F Change | df1 | df2 | Sig. F Change |
| 1 | ,401^a^ | ,161 | ,140 | ,05537 | ,161 | 7,666 | 1 | 40 | ,008 |
| 2 | ,457^b^ | ,209 | ,168 | ,05445 | ,048 | 2,364 | 1 | 39 | ,132 |
| a. Predictors: (Constant), Gender | | | | | | | | | |
| b. Predictors: (Constant), Gender, Triglycerides | | | | | | | | | |

| **Coefficients^a^** | | | | | | | | |
| --- | --- | --- | --- | --- | --- | --- | --- | --- |
| Model | | Unstandardized Coefficients | | Standardized Coefficients | t | Sig. | 95,0% Confidence Interval for B | |
|  |  | B | Std. Error | Beta |  |  | Lower Bound | Upper Bound |
| 1 | (Constant) | 2,554 | ,018 |  | 138,365 | ,000 | 2,517 | 2,591 |
|  | Gender | ,058 | ,021 | ,401 | 2,769 | ,008 | ,016 | ,100 |
| 2 | (Constant) | 2,547 | ,019 |  | 136,094 | ,000 | 2,509 | 2,585 |
|  | Gender | ,062 | ,021 | ,430 | 2,992 | ,005 | ,020 | ,104 |
|  | Triglycerides | ,070 | ,045 | ,221 | 1,537 | ,132 | -,022 | ,161 |
| a. Dependent Variable: CPT_RT | | | | | | | | |

| **Model Summary** | | | | | | | | | |
| --- | --- | --- | --- | --- | --- | --- | --- | --- | --- |
| Model | R | R Square | Adjusted R Square | Std. Error of the Estimate | Change Statistics | | | | |
|  |  |  |  |  | R Square Change | F Change | df1 | df2 | Sig. F Change |
| 1 | ,401^a^ | ,161 | ,140 | ,05537 | ,161 | 7,666 | 1 | 40 | ,008 |
| 2 | ,464^b^ | ,216 | ,175 | ,05422 | ,055 | 2,727 | 1 | 39 | ,107 |
| a. Predictors: (Constant), Gender | | | | | | | | | |
| b. Predictors: (Constant), Gender, HDL_cholesterol | | | | | | | | | |

| **Coefficients^a^** | | | | | | | | |
| --- | --- | --- | --- | --- | --- | --- | --- | --- |
| Model | | Unstandardized Coefficients | | Standardized Coefficients | t | Sig. | 95,0% Confidence Interval for B | |
|  |  | B | Std. Error | Beta |  |  | Lower Bound | Upper Bound |
| 1 | (Constant) | 2,554 | ,018 |  | 138,365 | ,000 | 2,517 | 2,591 |
|  | Gender | ,058 | ,021 | ,401 | 2,769 | ,008 | ,016 | ,100 |
| 2 | (Constant) | 2,611 | ,039 |  | 67,078 | ,000 | 2,532 | 2,690 |
|  | Gender | ,063 | ,021 | ,437 | 3,045 | ,004 | ,021 | ,105 |
|  | HDL_cholesterol | -,039 | ,023 | -,237 | -1,651 | ,107 | -,086 | ,009 |
| a. Dependent Variable: CPT_RT | | | | | | | | |

| **Model Summary** | | | | | | | | | |
| --- | --- | --- | --- | --- | --- | --- | --- | --- | --- |
| Model | R | R Square | Adjusted R Square | Std. Error of the Estimate | Change Statistics | | | | |
|  |  |  |  |  | R Square Change | F Change | df1 | df2 | Sig. F Change |
| 1 | ,401^a^ | ,161 | ,140 | ,05537 | ,161 | 7,666 | 1 | 40 | ,008 |
| 2 | ,431^b^ | ,186 | ,144 | ,05524 | ,025 | 1,188 | 1 | 39 | ,282 |
| a. Predictors: (Constant), Gender | | | | | | | | | |
| b. Predictors: (Constant), Gender, LDL_cholesterol | | | | | | | | | |

| **Coefficients^a^** | | | | | | | | |
| --- | --- | --- | --- | --- | --- | --- | --- | --- |
| Model | | Unstandardized Coefficients | | Standardized Coefficients | t | Sig. | 95,0% Confidence Interval for B | |
|  |  | B | Std. Error | Beta |  |  | Lower Bound | Upper Bound |
| 1 | (Constant) | 2,554 | ,018 |  | 138,365 | ,000 | 2,517 | 2,591 |
|  | Gender | ,058 | ,021 | ,401 | 2,769 | ,008 | ,016 | ,100 |
| 2 | (Constant) | 2,518 | ,037 |  | 67,288 | ,000 | 2,443 | 2,594 |
|  | Gender | ,058 | ,021 | ,406 | 2,807 | ,008 | ,016 | ,100 |
|  | LDL_cholesterol | ,012 | ,011 | ,158 | 1,090 | ,282 | -,010 | ,034 |
| a. Dependent Variable: CPT_RT | | | | | | | | |

| **Model Summary** | | | | | | | | | |
| --- | --- | --- | --- | --- | --- | --- | --- | --- | --- |
| Model | R | R Square | Adjusted R Square | Std. Error of the Estimate | Change Statistics | | | | |
|  |  |  |  |  | R Square Change | F Change | df1 | df2 | Sig. F Change |
| 1 | ,401^a^ | ,161 | ,140 | ,05537 | ,161 | 7,666 | 1 | 40 | ,008 |
| 2 | ,494^b^ | ,244 | ,205 | ,05323 | ,083 | 4,291 | 1 | 39 | ,045 |
| a. Predictors: (Constant), Gender | | | | | | | | | |
| b. Predictors: (Constant), Gender, LDL_C_HDL_C_ratio | | | | | | | | | |

| **Coefficients^a^** | | | | | | | | |
| --- | --- | --- | --- | --- | --- | --- | --- | --- |
| Model | | Unstandardized Coefficients | | Standardized Coefficients | t | Sig. | 95,0% Confidence Interval for B | |
|  |  | B | Std. Error | Beta |  |  | Lower Bound | Upper Bound |
| 1 | (Constant) | 2,554 | ,018 |  | 138,365 | ,000 | 2,517 | 2,591 |
|  | Gender | ,058 | ,021 | ,401 | 2,769 | ,008 | ,016 | ,100 |
| 2 | (Constant) | 2,518 | ,025 |  | 101,549 | ,000 | 2,468 | 2,568 |
|  | Gender | ,063 | ,020 | ,436 | 3,107 | ,004 | ,022 | ,103 |
|  | LDL_C_HDL_C_ratio | ,120 | ,058 | ,288 | 2,072 | ,056 | ,003 | ,236 |
| a. Dependent Variable: CPT_RT | | | | | | | | |

| **Model Summary** | | | | | | | | | |
| --- | --- | --- | --- | --- | --- | --- | --- | --- | --- |
| Model | R | R Square | Adjusted R Square | Std. Error of the Estimate | Change Statistics | | | | |
|  |  |  |  |  | R Square Change | F Change | df1 | df2 | Sig. F Change |
| 1 | ,401^a^ | ,161 | ,140 | ,05537 | ,161 | 7,666 | 1 | 40 | ,008 |
| 2 | ,497^b^ | ,247 | ,208 | ,05312 | ,086 | 4,459 | 1 | 39 | ,041 |
| a. Predictors: (Constant), Gender | | | | | | | | | |
| b. Predictors: (Constant), Gender, TC_HDL_C_ratio | | | | | | | | | |

| **Coefficients^a^** | | | | | | | | |
| --- | --- | --- | --- | --- | --- | --- | --- | --- |
| Model | | Unstandardized Coefficients | | Standardized Coefficients | t | Sig. | 95,0% Confidence Interval for B | |
|  |  | B | Std. Error | Beta |  |  | Lower Bound | Upper Bound |
| 1 | (Constant) | 2,554 | ,018 |  | 138,365 | ,000 | 2,517 | 2,591 |
|  | Gender | ,058 | ,021 | ,401 | 2,769 | ,008 | ,016 | ,100 |
| 2 | (Constant) | 2,468 | ,044 |  | 55,493 | ,000 | 2,378 | 2,558 |
|  | Gender | ,063 | ,020 | ,438 | 3,126 | ,003 | ,022 | ,104 |
|  | TC_HDL_C_ratio | ,161 | ,076 | ,291 | 2,112 | ,054 | ,007 | ,315 |
| a. Dependent Variable: CPT_RT | | | | | | | | |

| **Model Summary** | | | | | | | | | |
| --- | --- | --- | --- | --- | --- | --- | --- | --- | --- |
| Model | R | R Square | Adjusted R Square | Std. Error of the Estimate | Change Statistics | | | | |
|  |  |  |  |  | R Square Change | F Change | df1 | df2 | Sig. F Change |
| 1 | ,424^a^ | ,180 | ,160 | ,40698 | ,180 | 8,792 | 1 | 40 | ,005 |
| 2 | ,522^b^ | ,273 | ,235 | ,38820 | ,093 | 4,964 | 1 | 39 | ,032 |
| 3 | ,607^c^ | ,368 | ,318 | ,36666 | ,095 | 5,716 | 1 | 38 | ,022 |
| 4 | ,674^d^ | ,455 | ,396 | ,34516 | ,087 | 5,883 | 1 | 37 | ,020 |
| 5 | ,702^e^ | ,493 | ,423 | ,33725 | ,039 | 2,757 | 1 | 36 | ,106 |
| a. Predictors: (Constant), Age | | | | | | | | | |
| b. Predictors: (Constant), Age, Gender | | | | | | | | | |
| c. Predictors: (Constant), Age, Gender, Education | | | | | | | | | |
| d. Predictors: (Constant), Age, Gender, Education, Depression_severity | | | | | | | | | |
| e. Predictors: (Constant), Age, Gender, Education, Depression_severity, Total_cholesterol | | | | | | | | | |

| **Coefficients^a^** | | | | | | | | |
| --- | --- | --- | --- | --- | --- | --- | --- | --- |
| Model | | Unstandardized Coefficients | | Standardized Coefficients | t | Sig. | 95,0% Confidence Interval for B | |
|  |  | B | Std. Error | Beta |  |  | Lower Bound | Upper Bound |
| 1 | (Constant) | 1,349 | ,240 |  | 5,628 | ,000 | ,864 | 1,833 |
|  | Age | -,019 | ,007 | -,424 | -2,965 | ,005 | -,033 | -,006 |
| 2 | (Constant) | 1,167 | ,243 |  | 4,808 | ,000 | ,676 | 1,658 |
|  | Age | -,022 | ,006 | -,472 | -3,416 | ,001 | -,034 | -,009 |
|  | Gender | ,329 | ,148 | ,308 | 2,228 | ,032 | ,030 | ,628 |
| 3 | (Constant) | ,565 | ,341 |  | 1,659 | ,105 | -,124 | 1,254 |
|  | Age | -,023 | ,006 | -,513 | -3,899 | ,000 | -,036 | -,011 |
|  | Gender | ,345 | ,140 | ,323 | 2,468 | ,018 | ,062 | ,628 |
|  | Education | ,054 | ,023 | ,311 | 2,391 | ,022 | ,008 | ,100 |
| 4 | (Constant) | ,958 | ,359 |  | 2,666 | ,011 | ,230 | 1,685 |
|  | Age | -,022 | ,006 | -,491 | -3,953 | ,000 | -,034 | -,011 |
|  | Gender | ,369 | ,132 | ,345 | 2,794 | ,008 | ,101 | ,636 |
|  | Education | ,042 | ,022 | ,242 | 1,927 | ,062 | -,002 | ,087 |
|  | Depression_severity | -,013 | ,005 | -,304 | -2,425 | ,020 | -,024 | -,002 |
| 5 | (Constant) | ,614 | ,407 |  | 1,507 | ,141 | -,212 | 1,440 |
|  | Age | -,026 | ,006 | -,580 | -4,372 | ,000 | -,039 | -,014 |
|  | Gender | ,379 | ,129 | ,355 | 2,940 | ,006 | ,118 | ,641 |
|  | Education | ,043 | ,021 | ,246 | 2,004 | ,053 | -,001 | ,086 |
|  | Depression_severity | -,012 | ,005 | -,286 | -2,326 | ,026 | -,023 | -,002 |
|  | Total_cholesterol | ,089 | ,053 | ,216 | 1,660 | ,106 | -,020 | ,197 |
| a. Dependent Variable: CPT_Det | | | | | | | | |

| **Model Summary** | | | | | | | | | |
| --- | --- | --- | --- | --- | --- | --- | --- | --- | --- |
| Model | R | R Square | Adjusted R Square | Std. Error of the Estimate | Change Statistics | | | | |
|  |  |  |  |  | R Square Change | F Change | df1 | df2 | Sig. F Change |
| 1 | ,424^a^ | ,180 | ,160 | ,40698 | ,180 | 8,792 | 1 | 40 | ,005 |
| 2 | ,522^b^ | ,273 | ,235 | ,38820 | ,093 | 4,964 | 1 | 39 | ,032 |
| 3 | ,607^c^ | ,368 | ,318 | ,36666 | ,095 | 5,716 | 1 | 38 | ,022 |
| 4 | ,674^d^ | ,455 | ,396 | ,34516 | ,087 | 5,883 | 1 | 37 | ,020 |
| 5 | ,675^e^ | ,455 | ,380 | ,34972 | ,001 | ,041 | 1 | 36 | ,841 |
| a. Predictors: (Constant), Age | | | | | | | | | |
| b. Predictors: (Constant), Age, Gender | | | | | | | | | |
| c. Predictors: (Constant), Age, Gender, Education | | | | | | | | | |
| d. Predictors: (Constant), Age, Gender, Education, Depression_severity | | | | | | | | | |
| e. Predictors: (Constant), Age, Gender, Education, Depression_severity, Triglycerides | | | | | | | | | |

| **Coefficients^a^** | | | | | | | | |
| --- | --- | --- | --- | --- | --- | --- | --- | --- |
| Model | | Unstandardized Coefficients | | Standardized Coefficients | t | Sig. | 95,0% Confidence Interval for B | |
|  |  | B | Std. Error | Beta |  |  | Lower Bound | Upper Bound |
| 1 | (Constant) | 1,349 | ,240 |  | 5,628 | ,000 | ,864 | 1,833 |
|  | Age | -,019 | ,007 | -,424 | -2,965 | ,005 | -,033 | -,006 |
| 2 | (Constant) | 1,167 | ,243 |  | 4,808 | ,000 | ,676 | 1,658 |
|  | Age | -,022 | ,006 | -,472 | -3,416 | ,001 | -,034 | -,009 |
|  | Gender | ,329 | ,148 | ,308 | 2,228 | ,032 | ,030 | ,628 |
| 3 | (Constant) | ,565 | ,341 |  | 1,659 | ,105 | -,124 | 1,254 |
|  | Age | -,023 | ,006 | -,513 | -3,899 | ,000 | -,036 | -,011 |
|  | Gender | ,345 | ,140 | ,323 | 2,468 | ,018 | ,062 | ,628 |
|  | Education | ,054 | ,023 | ,311 | 2,391 | ,022 | ,008 | ,100 |
| 4 | (Constant) | ,958 | ,359 |  | 2,666 | ,011 | ,230 | 1,685 |
|  | Age | -,022 | ,006 | -,491 | -3,953 | ,000 | -,034 | -,011 |
|  | Gender | ,369 | ,132 | ,345 | 2,794 | ,008 | ,101 | ,636 |
|  | Education | ,042 | ,022 | ,242 | 1,927 | ,062 | -,002 | ,087 |
|  | Depression_severity | -,013 | ,005 | -,304 | -2,425 | ,020 | -,024 | -,002 |
| 5 | (Constant) | ,971 | ,370 |  | 2,625 | ,013 | ,221 | 1,721 |
|  | Age | -,023 | ,006 | -,500 | -3,779 | ,001 | -,035 | -,011 |
|  | Gender | ,374 | ,137 | ,350 | 2,738 | ,010 | ,097 | ,652 |
|  | Education | ,042 | ,022 | ,242 | 1,896 | ,066 | -,003 | ,087 |
|  | Depression_severity | -,013 | ,006 | -,311 | -2,359 | ,024 | -,025 | -,002 |
|  | Triglycerides | ,064 | ,318 | ,027 | ,202 | ,841 | -,581 | ,709 |
| a. Dependent Variable: CPT_Det | | | | | | | | |

| **Model Summary** | | | | | | | | | |
| --- | --- | --- | --- | --- | --- | --- | --- | --- | --- |
| Model | R | R Square | Adjusted R Square | Std. Error of the Estimate | Change Statistics | | | | |
|  |  |  |  |  | R Square Change | F Change | df1 | df2 | Sig. F Change |
| 1 | ,424^a^ | ,180 | ,160 | ,40698 | ,180 | 8,792 | 1 | 40 | ,005 |
| 2 | ,522^b^ | ,273 | ,235 | ,38820 | ,093 | 4,964 | 1 | 39 | ,032 |
| 3 | ,607^c^ | ,368 | ,318 | ,36666 | ,095 | 5,716 | 1 | 38 | ,022 |
| 4 | ,674^d^ | ,455 | ,396 | ,34516 | ,087 | 5,883 | 1 | 37 | ,020 |
| 5 | ,676^e^ | ,457 | ,381 | ,34927 | ,002 | ,134 | 1 | 36 | ,716 |
| a. Predictors: (Constant), Age | | | | | | | | | |
| b. Predictors: (Constant), Age, Gender | | | | | | | | | |
| c. Predictors: (Constant), Age, Gender, Education | | | | | | | | | |
| d. Predictors: (Constant), Age, Gender, Education, Depression_severity | | | | | | | | | |
| e. Predictors: (Constant), Age, Gender, Education, Depression_severity, HDL_cholesterol | | | | | | | | | |

| **Coefficients^a^** | | | | | | | | |
| --- | --- | --- | --- | --- | --- | --- | --- | --- |
| Model | | Unstandardized Coefficients | | Standardized Coefficients | t | Sig. | 95,0% Confidence Interval for B | |
|  |  | B | Std. Error | Beta |  |  | Lower Bound | Upper Bound |
| 1 | (Constant) | 1,349 | ,240 |  | 5,628 | ,000 | ,864 | 1,833 |
|  | Age | -,019 | ,007 | -,424 | -2,965 | ,005 | -,033 | -,006 |
| 2 | (Constant) | 1,167 | ,243 |  | 4,808 | ,000 | ,676 | 1,658 |
|  | Age | -,022 | ,006 | -,472 | -3,416 | ,001 | -,034 | -,009 |
|  | Gender | ,329 | ,148 | ,308 | 2,228 | ,032 | ,030 | ,628 |
| 3 | (Constant) | ,565 | ,341 |  | 1,659 | ,105 | -,124 | 1,254 |
|  | Age | -,023 | ,006 | -,513 | -3,899 | ,000 | -,036 | -,011 |
|  | Gender | ,345 | ,140 | ,323 | 2,468 | ,018 | ,062 | ,628 |
|  | Education | ,054 | ,023 | ,311 | 2,391 | ,022 | ,008 | ,100 |
| 4 | (Constant) | ,958 | ,359 |  | 2,666 | ,011 | ,230 | 1,685 |
|  | Age | -,022 | ,006 | -,491 | -3,953 | ,000 | -,034 | -,011 |
|  | Gender | ,369 | ,132 | ,345 | 2,794 | ,008 | ,101 | ,636 |
|  | Education | ,042 | ,022 | ,242 | 1,927 | ,062 | -,002 | ,087 |
|  | Depression_severity | -,013 | ,005 | -,304 | -2,425 | ,020 | -,024 | -,002 |
| 5 | (Constant) | 1,054 | ,448 |  | 2,351 | ,024 | ,145 | 1,963 |
|  | Age | -,022 | ,006 | -,483 | -3,767 | ,001 | -,034 | -,010 |
|  | Gender | ,377 | ,136 | ,353 | 2,782 | ,009 | ,102 | ,652 |
|  | Education | ,042 | ,022 | ,243 | 1,909 | ,064 | -,003 | ,087 |
|  | Depression_severity | -,014 | ,006 | -,325 | -2,332 | ,025 | -,026 | -,002 |
|  | HDL_cholesterol | -,062 | ,169 | -,051 | -,366 | ,716 | -,404 | ,280 |
| a. Dependent Variable: CPT_Det | | | | | | | | |

| **Model Summary** | | | | | | | | | |
| --- | --- | --- | --- | --- | --- | --- | --- | --- | --- |
| Model | R | R Square | Adjusted R Square | Std. Error of the Estimate | Change Statistics | | | | |
|  |  |  |  |  | R Square Change | F Change | df1 | df2 | Sig. F Change |
| 1 | ,424^a^ | ,180 | ,160 | ,40698 | ,180 | 8,792 | 1 | 40 | ,005 |
| 2 | ,522^b^ | ,273 | ,235 | ,38820 | ,093 | 4,964 | 1 | 39 | ,032 |
| 3 | ,607^c^ | ,368 | ,318 | ,36666 | ,095 | 5,716 | 1 | 38 | ,022 |
| 4 | ,674^d^ | ,455 | ,396 | ,34516 | ,087 | 5,883 | 1 | 37 | ,020 |
| 5 | ,708^e^ | ,501 | ,432 | ,33468 | ,046 | 3,354 | 1 | 36 | ,075 |
| a. Predictors: (Constant), Age | | | | | | | | | |
| b. Predictors: (Constant), Age, Gender | | | | | | | | | |
| c. Predictors: (Constant), Age, Gender, Education | | | | | | | | | |
| d. Predictors: (Constant), Age, Gender, Education, Depression_severity | | | | | | | | | |
| e. Predictors: (Constant), Age, Gender, Education, Depression_severity, LDL_cholesterol | | | | | | | | | |

| **Coefficients^a^** | | | | | | | | |
| --- | --- | --- | --- | --- | --- | --- | --- | --- |
| Model | | Unstandardized Coefficients | | Standardized Coefficients | t | Sig. | 95,0% Confidence Interval for B | |
|  |  | B | Std. Error | Beta |  |  | Lower Bound | Upper Bound |
| 1 | (Constant) | 1,349 | ,240 |  | 5,628 | ,000 | ,864 | 1,833 |
|  | Age | -,019 | ,007 | -,424 | -2,965 | ,005 | -,033 | -,006 |
| 2 | (Constant) | 1,167 | ,243 |  | 4,808 | ,000 | ,676 | 1,658 |
|  | Age | -,022 | ,006 | -,472 | -3,416 | ,001 | -,034 | -,009 |
|  | Gender | ,329 | ,148 | ,308 | 2,228 | ,032 | ,030 | ,628 |
| 3 | (Constant) | ,565 | ,341 |  | 1,659 | ,105 | -,124 | 1,254 |
|  | Age | -,023 | ,006 | -,513 | -3,899 | ,000 | -,036 | -,011 |
|  | Gender | ,345 | ,140 | ,323 | 2,468 | ,018 | ,062 | ,628 |
|  | Education | ,054 | ,023 | ,311 | 2,391 | ,022 | ,008 | ,100 |
| 4 | (Constant) | ,958 | ,359 |  | 2,666 | ,011 | ,230 | 1,685 |
|  | Age | -,022 | ,006 | -,491 | -3,953 | ,000 | -,034 | -,011 |
|  | Gender | ,369 | ,132 | ,345 | 2,794 | ,008 | ,101 | ,636 |
|  | Education | ,042 | ,022 | ,242 | 1,927 | ,062 | -,002 | ,087 |
|  | Depression_severity | -,013 | ,005 | -,304 | -2,425 | ,020 | -,024 | -,002 |
| 5 | (Constant) | ,700 | ,376 |  | 1,863 | ,071 | -,062 | 1,462 |
|  | Age | -,027 | ,006 | -,586 | -4,469 | ,000 | -,039 | -,015 |
|  | Gender | ,392 | ,129 | ,367 | 3,050 | ,004 | ,131 | ,653 |
|  | Education | ,043 | ,021 | ,244 | 2,003 | ,053 | -,001 | ,086 |
|  | Depression_severity | -,013 | ,005 | -,303 | -2,497 | ,017 | -,024 | -,002 |
|  | LDL_cholesterol | ,131 | ,072 | ,235 | 1,831 | ,075 | -,014 | ,277 |
| a. Dependent Variable: CPT_Det | | | | | | | | |

| **Model Summary** | | | | | | | | | |
| --- | --- | --- | --- | --- | --- | --- | --- | --- | --- |
| Model | R | R Square | Adjusted R Square | Std. Error of the Estimate | Change Statistics | | | | |
|  |  |  |  |  | R Square Change | F Change | df1 | df2 | Sig. F Change |
| 1 | ,424^a^ | ,180 | ,160 | ,40698 | ,180 | 8,792 | 1 | 40 | ,005 |
| 2 | ,522^b^ | ,273 | ,235 | ,38820 | ,093 | 4,964 | 1 | 39 | ,032 |
| 3 | ,607^c^ | ,368 | ,318 | ,36666 | ,095 | 5,716 | 1 | 38 | ,022 |
| 4 | ,674^d^ | ,455 | ,396 | ,34516 | ,087 | 5,883 | 1 | 37 | ,020 |
| 5 | ,700^e^ | ,490 | ,419 | ,33838 | ,035 | 2,496 | 1 | 36 | ,123 |
| a. Predictors: (Constant), Age | | | | | | | | | |
| b. Predictors: (Constant), Age, Gender | | | | | | | | | |
| c. Predictors: (Constant), Age, Gender, Education | | | | | | | | | |
| d. Predictors: (Constant), Age, Gender, Education, Depression_severity | | | | | | | | | |
| e. Predictors: (Constant), Age, Gender, Education, Depression_severity, LDL_C_HDL_C_ratio | | | | | | | | | |

| **Coefficients^a^** | | | | | | | | |
| --- | --- | --- | --- | --- | --- | --- | --- | --- |
| Model | | Unstandardized Coefficients | | Standardized Coefficients | t | Sig. | 95,0% Confidence Interval for B | |
|  |  | B | Std. Error | Beta |  |  | Lower Bound | Upper Bound |
| 1 | (Constant) | 1,349 | ,240 |  | 5,628 | ,000 | ,864 | 1,833 |
|  | Age | -,019 | ,007 | -,424 | -2,965 | ,005 | -,033 | -,006 |
| 2 | (Constant) | 1,167 | ,243 |  | 4,808 | ,000 | ,676 | 1,658 |
|  | Age | -,022 | ,006 | -,472 | -3,416 | ,001 | -,034 | -,009 |
|  | Gender | ,329 | ,148 | ,308 | 2,228 | ,032 | ,030 | ,628 |
| 3 | (Constant) | ,565 | ,341 |  | 1,659 | ,105 | -,124 | 1,254 |
|  | Age | -,023 | ,006 | -,513 | -3,899 | ,000 | -,036 | -,011 |
|  | Gender | ,345 | ,140 | ,323 | 2,468 | ,018 | ,062 | ,628 |
|  | Education | ,054 | ,023 | ,311 | 2,391 | ,022 | ,008 | ,100 |
| 4 | (Constant) | ,958 | ,359 |  | 2,666 | ,011 | ,230 | 1,685 |
|  | Age | -,022 | ,006 | -,491 | -3,953 | ,000 | -,034 | -,011 |
|  | Gender | ,369 | ,132 | ,345 | 2,794 | ,008 | ,101 | ,636 |
|  | Education | ,042 | ,022 | ,242 | 1,927 | ,062 | -,002 | ,087 |
|  | Depression_severity | -,013 | ,005 | -,304 | -2,425 | ,020 | -,024 | -,002 |
| 5 | (Constant) | ,883 | ,355 |  | 2,486 | ,018 | ,163 | 1,604 |
|  | Age | -,024 | ,006 | -,531 | -4,269 | ,000 | -,036 | -,013 |
|  | Gender | ,407 | ,132 | ,380 | 3,092 | ,004 | ,140 | ,673 |
|  | Education | ,042 | ,022 | ,243 | 1,971 | ,056 | -,001 | ,086 |
|  | Depression_severity | -,016 | ,005 | -,363 | -2,827 | ,008 | -,027 | -,004 |
|  | LDL_C_HDL_C_ratio | ,621 | ,393 | ,203 | 1,580 | ,123 | -,176 | 1,418 |
| a. Dependent Variable: CPT_Det | | | | | | | | |

| **Model Summary** | | | | | | | | | |
| --- | --- | --- | --- | --- | --- | --- | --- | --- | --- |
| Model | R | R Square | Adjusted R Square | Std. Error of the Estimate | Change Statistics | | | | |
|  |  |  |  |  | R Square Change | F Change | df1 | df2 | Sig. F Change |
| 1 | ,424^a^ | ,180 | ,160 | ,40698 | ,180 | 8,792 | 1 | 40 | ,005 |
| 2 | ,522^b^ | ,273 | ,235 | ,38820 | ,093 | 4,964 | 1 | 39 | ,032 |
| 3 | ,607^c^ | ,368 | ,318 | ,36666 | ,095 | 5,716 | 1 | 38 | ,022 |
| 4 | ,674^d^ | ,455 | ,396 | ,34516 | ,087 | 5,883 | 1 | 37 | ,020 |
| 5 | ,703^e^ | ,495 | ,425 | ,33680 | ,040 | 2,859 | 1 | 36 | ,100 |
| a. Predictors: (Constant), Age | | | | | | | | | |
| b. Predictors: (Constant), Age, Gender | | | | | | | | | |
| c. Predictors: (Constant), Age, Gender, Education | | | | | | | | | |
| d. Predictors: (Constant), Age, Gender, Education, Depression_severity | | | | | | | | | |
| e. Predictors: (Constant), Age, Gender, Education, Depression_severity, TC_HDL_C_ratio | | | | | | | | | |

| **Coefficients^a^** | | | | | | | | |
| --- | --- | --- | --- | --- | --- | --- | --- | --- |
| Model | | Unstandardized Coefficients | | Standardized Coefficients | t | Sig. | 95,0% Confidence Interval for B | |
|  |  | B | Std. Error | Beta |  |  | Lower Bound | Upper Bound |
| 1 | (Constant) | 1,349 | ,240 |  | 5,628 | ,000 | ,864 | 1,833 |
|  | Age | -,019 | ,007 | -,424 | -2,965 | ,005 | -,033 | -,006 |
| 2 | (Constant) | 1,167 | ,243 |  | 4,808 | ,000 | ,676 | 1,658 |
|  | Age | -,022 | ,006 | -,472 | -3,416 | ,001 | -,034 | -,009 |
|  | Gender | ,329 | ,148 | ,308 | 2,228 | ,032 | ,030 | ,628 |
| 3 | (Constant) | ,565 | ,341 |  | 1,659 | ,105 | -,124 | 1,254 |
|  | Age | -,023 | ,006 | -,513 | -3,899 | ,000 | -,036 | -,011 |
|  | Gender | ,345 | ,140 | ,323 | 2,468 | ,018 | ,062 | ,628 |
|  | Education | ,054 | ,023 | ,311 | 2,391 | ,022 | ,008 | ,100 |
| 4 | (Constant) | ,958 | ,359 |  | 2,666 | ,011 | ,230 | 1,685 |
|  | Age | -,022 | ,006 | -,491 | -3,953 | ,000 | -,034 | -,011 |
|  | Gender | ,369 | ,132 | ,345 | 2,794 | ,008 | ,101 | ,636 |
|  | Education | ,042 | ,022 | ,242 | 1,927 | ,062 | -,002 | ,087 |
|  | Depression_severity | -,013 | ,005 | -,304 | -2,425 | ,020 | -,024 | -,002 |
| 5 | (Constant) | ,598 | ,410 |  | 1,460 | ,153 | -,233 | 1,429 |
|  | Age | -,024 | ,006 | -,534 | -4,310 | ,000 | -,036 | -,013 |
|  | Gender | ,411 | ,131 | ,384 | 3,134 | ,003 | ,145 | ,677 |
|  | Education | ,043 | ,021 | ,248 | 2,018 | ,051 | ,000 | ,087 |
|  | Depression_severity | -,016 | ,005 | -,370 | -2,882 | ,007 | -,027 | -,005 |
|  | TC_HDL_C_ratio | ,878 | ,519 | ,217 | 1,691 | ,100 | -,175 | 1,932 |
| a. Dependent Variable: CPT_Det | | | | | | | | |

## Supplementary Table 7.

## Hierarchical linear regression analyses of serum lipid and lipoprotein levels as predictors of executive functioning (WCST scores) in the entire MDD group

| **Model Summary** | | | | | | | | | |
| --- | --- | --- | --- | --- | --- | --- | --- | --- | --- |
| Model | R | R Square | Adjusted R Square | Std. Error of the Estimate | Change Statistics | | | | |
|  |  |  |  |  | R Square Change | F Change | df1 | df2 | Sig. F Change |
| 1 | ,130^a^ | ,017 | -,008 | ,05773 | ,017 | ,690 | 1 | 40 | ,411 |
| a. Predictors: (Constant), Total_cholesterol | | | | | | | | | |

| **Coefficients^a^** | | | | | | | | |
| --- | --- | --- | --- | --- | --- | --- | --- | --- |
| Model | | Unstandardized Coefficients | | Standardized Coefficients | t | Sig. | 95,0% Confidence Interval for B | |
|  |  | B | Std. Error | Beta |  |  | Lower Bound | Upper Bound |
| 1 | (Constant) | 1,884 | ,043 |  | 43,343 | ,000 | 1,796 | 1,972 |
|  | Total_cholesterol | -,007 | ,008 | -,130 | -,831 | ,411 | -,024 | ,010 |
| a. Dependent Variable: WCST_TC | | | | | | | | |

| **Model Summary** | | | | | | | | | |
| --- | --- | --- | --- | --- | --- | --- | --- | --- | --- |
| Model | R | R Square | Adjusted R Square | Std. Error of the Estimate | Change Statistics | | | | |
|  |  |  |  |  | R Square Change | F Change | df1 | df2 | Sig. F Change |
| 1 | ,235^a^ | ,055 | ,032 | ,05659 | ,055 | 2,345 | 1 | 40 | ,134 |
| a. Predictors: (Constant), Triglycerides | | | | | | | | | |

| **Coefficients^a^** | | | | | | | | |
| --- | --- | --- | --- | --- | --- | --- | --- | --- |
| Model | | Unstandardized Coefficients | | Standardized Coefficients | t | Sig. | 95,0% Confidence Interval for B | |
|  |  | B | Std. Error | Beta |  |  | Lower Bound | Upper Bound |
| 1 | (Constant) | 1,853 | ,009 |  | 203,902 | ,000 | 1,834 | 1,871 |
|  | Triglycerides | -,071 | ,047 | -,235 | -1,531 | ,134 | -,166 | ,023 |
| a. Dependent Variable: WCST_TC | | | | | | | | |

| **Model Summary** | | | | | | | | | |
| --- | --- | --- | --- | --- | --- | --- | --- | --- | --- |
| Model | R | R Square | Adjusted R Square | Std. Error of the Estimate | Change Statistics | | | | |
|  |  |  |  |  | R Square Change | F Change | df1 | df2 | Sig. F Change |
| 1 | ,167^a^ | ,028 | ,004 | ,05741 | ,028 | 1,149 | 1 | 40 | ,290 |
| a. Predictors: (Constant), HDL_cholesterol | | | | | | | | | |

| **Coefficients^a^** | | | | | | | | |
| --- | --- | --- | --- | --- | --- | --- | --- | --- |
| Model | | Unstandardized Coefficients | | Standardized Coefficients | t | Sig. | 95,0% Confidence Interval for B | |
|  |  | B | Std. Error | Beta |  |  | Lower Bound | Upper Bound |
| 1 | (Constant) | 1,807 | ,040 |  | 45,588 | ,000 | 1,727 | 1,887 |
|  | HDL_cholesterol | ,026 | ,024 | ,167 | 1,072 | ,290 | -,023 | ,076 |
| a. Dependent Variable: WCST_TC | | | | | | | | |

| **Model Summary** | | | | | | | | | |
| --- | --- | --- | --- | --- | --- | --- | --- | --- | --- |
| Model | R | R Square | Adjusted R Square | Std. Error of the Estimate | Change Statistics | | | | |
|  |  |  |  |  | R Square Change | F Change | df1 | df2 | Sig. F Change |
| 1 | ,236^a^ | ,056 | ,032 | ,05659 | ,056 | 2,352 | 1 | 40 | ,133 |
| a. Predictors: (Constant), LDL_cholesterol | | | | | | | | | |

| **Coefficients^a^** | | | | | | | | |
| --- | --- | --- | --- | --- | --- | --- | --- | --- |
| Model | | Unstandardized Coefficients | | Standardized Coefficients | t | Sig. | 95,0% Confidence Interval for B | |
|  |  | B | Std. Error | Beta |  |  | Lower Bound | Upper Bound |
| 1 | (Constant) | 1,899 | ,034 |  | 55,875 | ,000 | 1,830 | 1,968 |
|  | LDL_cholesterol | -,017 | ,011 | -,236 | -1,534 | ,133 | -,040 | ,005 |
| a. Dependent Variable: WCST_TC | | | | | | | | |

| **Model Summary** | | | | | | | | | |
| --- | --- | --- | --- | --- | --- | --- | --- | --- | --- |
| Model | R | R Square | Adjusted R Square | Std. Error of the Estimate | Change Statistics | | | | |
|  |  |  |  |  | R Square Change | F Change | df1 | df2 | Sig. F Change |
| 1 | ,306^a^ | ,094 | ,071 | ,05543 | ,094 | 4,144 | 1 | 40 | ,048 |
| a. Predictors: (Constant), LDL_C_HDL_C_ratio | | | | | | | | | |

| **Coefficients^a^** | | | | | | | | |
| --- | --- | --- | --- | --- | --- | --- | --- | --- |
| Model | | Unstandardized Coefficients | | Standardized Coefficients | t | Sig. | 95,0% Confidence Interval for B | |
|  |  | B | Std. Error | Beta |  |  | Lower Bound | Upper Bound |
| 1 | (Constant) | 1,881 | ,018 |  | 103,900 | ,000 | 1,845 | 1,918 |
|  | LDL_C_HDL_C_ratio | -,121 | ,060 | -,306 | -2,036 | ,048 | -,242 | -,001 |
| a. Dependent Variable: WCST_TC | | | | | | | | |

| **Model Summary** | | | | | | | | | |
| --- | --- | --- | --- | --- | --- | --- | --- | --- | --- |
| Model | R | R Square | Adjusted R Square | Std. Error of the Estimate | Change Statistics | | | | |
|  |  |  |  |  | R Square Change | F Change | df1 | df2 | Sig. F Change |
| 1 | ,252^a^ | ,063 | ,040 | ,05635 | ,063 | 2,710 | 1 | 40 | ,108 |
| a. Predictors: (Constant), TC_HDL_C_ratio | | | | | | | | | |

| **Coefficients^a^** | | | | | | | | |
| --- | --- | --- | --- | --- | --- | --- | --- | --- |
| Model | | Unstandardized Coefficients | | Standardized Coefficients | t | Sig. | 95,0% Confidence Interval for B | |
|  |  | B | Std. Error | Beta |  |  | Lower Bound | Upper Bound |
| 1 | (Constant) | 1,916 | ,042 |  | 45,857 | ,000 | 1,832 | 2,001 |
|  | TC_HDL_C_ratio | -,132 | ,080 | -,252 | -1,646 | ,108 | -,294 | ,030 |
| a. Dependent Variable: WCST_TC | | | | | | | | |

| **Model Summary** | | | | | | | | | |
| --- | --- | --- | --- | --- | --- | --- | --- | --- | --- |
| Model | R | R Square | Adjusted R Square | Std. Error of the Estimate | Change Statistics | | | | |
|  |  |  |  |  | R Square Change | F Change | df1 | df2 | Sig. F Change |
| 1 | ,444^a^ | ,197 | ,177 | ,32426 | ,197 | 9,827 | 1 | 40 | ,003 |
| 2 | ,606^b^ | ,368 | ,335 | ,29147 | ,170 | 10,506 | 1 | 39 | ,002 |
| 3 | ,612^c^ | ,374 | ,325 | ,29375 | ,007 | ,398 | 1 | 38 | ,532 |
| a. Predictors: (Constant), Education | | | | | | | | | |
| b. Predictors: (Constant), Education, Age | | | | | | | | | |
| c. Predictors: (Constant), Education, Age, Total_cholesterol | | | | | | | | | |

| **Coefficients^a^** | | | | | | | | |
| --- | --- | --- | --- | --- | --- | --- | --- | --- |
| Model | | Unstandardized Coefficients | | Standardized Coefficients | t | Sig. | 95,0% Confidence Interval for B | |
|  |  | B | Std. Error | Beta |  |  | Lower Bound | Upper Bound |
| 1 | (Constant) | 1,860 | ,246 |  | 7,568 | ,000 | 1,363 | 2,357 |
|  | Education | -,062 | ,020 | -,444 | -3,135 | ,003 | -,103 | -,022 |
| 2 | (Constant) | 1,408 | ,261 |  | 5,386 | ,000 | ,879 | 1,936 |
|  | Education | -,070 | ,018 | -,496 | -3,867 | ,000 | -,106 | -,033 |
|  | Age | ,015 | ,005 | ,416 | 3,241 | ,002 | ,006 | ,025 |
| 3 | (Constant) | 1,511 | ,310 |  | 4,876 | ,000 | ,883 | 2,138 |
|  | Education | -,070 | ,018 | -,496 | -3,835 | ,000 | -,106 | -,033 |
|  | Age | ,017 | ,005 | ,451 | 3,205 | ,003 | ,006 | ,027 |
|  | Total_cholesterol | -,029 | ,046 | -,088 | -,631 | ,532 | -,123 | ,064 |
| a. Dependent Variable: WCST_PE | | | | | | | | |

| **Model Summary** | | | | | | | | | |
| --- | --- | --- | --- | --- | --- | --- | --- | --- | --- |
| Model | R | R Square | Adjusted R Square | Std. Error of the Estimate | Change Statistics | | | | |
|  |  |  |  |  | R Square Change | F Change | df1 | df2 | Sig. F Change |
| 1 | ,444^a^ | ,197 | ,177 | ,32426 | ,197 | 9,827 | 1 | 40 | ,003 |
| 2 | ,606^b^ | ,368 | ,335 | ,29147 | ,170 | 10,506 | 1 | 39 | ,002 |
| 3 | ,642^c^ | ,412 | ,365 | ,28482 | ,044 | 2,842 | 1 | 38 | ,100 |
| a. Predictors: (Constant), Education | | | | | | | | | |
| b. Predictors: (Constant), Education, Age | | | | | | | | | |
| c. Predictors: (Constant), Education, Age, Triglycerides | | | | | | | | | |

| **Coefficients^a^** | | | | | | | | |
| --- | --- | --- | --- | --- | --- | --- | --- | --- |
| Model | | Unstandardized Coefficients | | Standardized Coefficients | t | Sig. | 95,0% Confidence Interval for B | |
|  |  | B | Std. Error | Beta |  |  | Lower Bound | Upper Bound |
| 1 | (Constant) | 1,860 | ,246 |  | 7,568 | ,000 | 1,363 | 2,357 |
|  | Education | -,062 | ,020 | -,444 | -3,135 | ,003 | -,103 | -,022 |
| 2 | (Constant) | 1,408 | ,261 |  | 5,386 | ,000 | ,879 | 1,936 |
|  | Education | -,070 | ,018 | -,496 | -3,867 | ,000 | -,106 | -,033 |
|  | Age | ,015 | ,005 | ,416 | 3,241 | ,002 | ,006 | ,025 |
| 3 | (Constant) | 1,456 | ,257 |  | 5,666 | ,000 | ,936 | 1,976 |
|  | Education | -,069 | ,018 | -,490 | -3,905 | ,000 | -,104 | -,033 |
|  | Age | ,013 | ,005 | ,353 | 2,702 | ,010 | ,003 | ,023 |
|  | Triglycerides | ,413 | ,245 | ,219 | 1,686 | ,100 | -,083 | ,909 |
| a. Dependent Variable: WCST_PE | | | | | | | | |

| **Model Summary** | | | | | | | | | |
| --- | --- | --- | --- | --- | --- | --- | --- | --- | --- |
| Model | R | R Square | Adjusted R Square | Std. Error of the Estimate | Change Statistics | | | | |
|  |  |  |  |  | R Square Change | F Change | df1 | df2 | Sig. F Change |
| 1 | ,444^a^ | ,197 | ,177 | ,32426 | ,197 | 9,827 | 1 | 40 | ,003 |
| 2 | ,606^b^ | ,368 | ,335 | ,29147 | ,170 | 10,506 | 1 | 39 | ,002 |
| 3 | ,667^c^ | ,445 | ,401 | ,27672 | ,077 | 5,268 | 1 | 38 | ,027 |
| a. Predictors: (Constant), Education | | | | | | | | | |
| b. Predictors: (Constant), Education, Age | | | | | | | | | |
| c. Predictors: (Constant), Education, Age, HDL_cholesterol | | | | | | | | | |

| **Coefficients^a^** | | | | | | | | |
| --- | --- | --- | --- | --- | --- | --- | --- | --- |
| Model | | Unstandardized Coefficients | | Standardized Coefficients | t | Sig. | 95,0% Confidence Interval for B | |
|  |  | B | Std. Error | Beta |  |  | Lower Bound | Upper Bound |
| 1 | (Constant) | 1,860 | ,246 |  | 7,568 | ,000 | 1,363 | 2,357 |
|  | Education | -,062 | ,020 | -,444 | -3,135 | ,003 | -,103 | -,022 |
| 2 | (Constant) | 1,408 | ,261 |  | 5,386 | ,000 | ,879 | 1,936 |
|  | Education | -,070 | ,018 | -,496 | -3,867 | ,000 | -,106 | -,033 |
|  | Age | ,015 | ,005 | ,416 | 3,241 | ,002 | ,006 | ,025 |
| 3 | (Constant) | 1,735 | ,286 |  | 6,062 | ,000 | 1,156 | 2,315 |
|  | Education | -,066 | ,017 | -,468 | -3,819 | ,000 | -,101 | -,031 |
|  | Age | ,017 | ,005 | ,463 | 3,745 | ,001 | ,008 | ,026 |
|  | HDL_cholesterol | -,277 | ,120 | -,283 | -2,295 | ,027 | -,520 | -,033 |
| a. Dependent Variable: WCST_PE | | | | | | | | |

| **Model Summary** | | | | | | | | | |
| --- | --- | --- | --- | --- | --- | --- | --- | --- | --- |
| Model | R | R Square | Adjusted R Square | Std. Error of the Estimate | Change Statistics | | | | |
|  |  |  |  |  | R Square Change | F Change | df1 | df2 | Sig. F Change |
| 1 | ,444^a^ | ,197 | ,177 | ,32426 | ,197 | 9,827 | 1 | 40 | ,003 |
| 2 | ,606^b^ | ,368 | ,335 | ,29147 | ,170 | 10,506 | 1 | 39 | ,002 |
| 3 | ,610^c^ | ,372 | ,322 | ,29436 | ,004 | ,237 | 1 | 38 | ,629 |
| a. Predictors: (Constant), Education | | | | | | | | | |
| b. Predictors: (Constant), Education, Age | | | | | | | | | |
| c. Predictors: (Constant), Education, Age, LDL_cholesterol | | | | | | | | | |

| **Coefficients^a^** | | | | | | | | |
| --- | --- | --- | --- | --- | --- | --- | --- | --- |
| Model | | Unstandardized Coefficients | | Standardized Coefficients | t | Sig. | 95,0% Confidence Interval for B | |
|  |  | B | Std. Error | Beta |  |  | Lower Bound | Upper Bound |
| 1 | (Constant) | 1,860 | ,246 |  | 7,568 | ,000 | 1,363 | 2,357 |
|  | Education | -,062 | ,020 | -,444 | -3,135 | ,003 | -,103 | -,022 |
| 2 | (Constant) | 1,408 | ,261 |  | 5,386 | ,000 | ,879 | 1,936 |
|  | Education | -,070 | ,018 | -,496 | -3,867 | ,000 | -,106 | -,033 |
|  | Age | ,015 | ,005 | ,416 | 3,241 | ,002 | ,006 | ,025 |
| 3 | (Constant) | 1,464 | ,288 |  | 5,080 | ,000 | ,881 | 2,047 |
|  | Education | -,070 | ,018 | -,497 | -3,830 | ,000 | -,107 | -,033 |
|  | Age | ,016 | ,005 | ,442 | 3,148 | ,003 | ,006 | ,027 |
|  | LDL_cholesterol | -,031 | ,063 | -,068 | -,487 | ,629 | -,158 | ,097 |
| a. Dependent Variable: WCST_PE | | | | | | | | |

| **Model Summary** | | | | | | | | | |
| --- | --- | --- | --- | --- | --- | --- | --- | --- | --- |
| Model | R | R Square | Adjusted R Square | Std. Error of the Estimate | Change Statistics | | | | |
|  |  |  |  |  | R Square Change | F Change | df1 | df2 | Sig. F Change |
| 1 | ,444^a^ | ,197 | ,177 | ,32426 | ,197 | 9,827 | 1 | 40 | ,003 |
| 2 | ,606^b^ | ,368 | ,335 | ,29147 | ,170 | 10,506 | 1 | 39 | ,002 |
| 3 | ,624^c^ | ,389 | ,341 | ,29014 | ,022 | 1,359 | 1 | 38 | ,251 |
| a. Predictors: (Constant), Education | | | | | | | | | |
| b. Predictors: (Constant), Education, Age | | | | | | | | | |
| c. Predictors: (Constant), Education, Age, LDL_C_HDL_C_ratio | | | | | | | | | |

| **Coefficients^a^** | | | | | | | | |
| --- | --- | --- | --- | --- | --- | --- | --- | --- |
| Model | | Unstandardized Coefficients | | Standardized Coefficients | t | Sig. | 95,0% Confidence Interval for B | |
|  |  | B | Std. Error | Beta |  |  | Lower Bound | Upper Bound |
| 1 | (Constant) | 1,860 | ,246 |  | 7,568 | ,000 | 1,363 | 2,357 |
|  | Education | -,062 | ,020 | -,444 | -3,135 | ,003 | -,103 | -,022 |
| 2 | (Constant) | 1,408 | ,261 |  | 5,386 | ,000 | ,879 | 1,936 |
|  | Education | -,070 | ,018 | -,496 | -3,867 | ,000 | -,106 | -,033 |
|  | Age | ,015 | ,005 | ,416 | 3,241 | ,002 | ,006 | ,025 |
| 3 | (Constant) | 1,330 | ,268 |  | 4,954 | ,000 | ,787 | 1,874 |
|  | Education | -,068 | ,018 | -,487 | -3,803 | ,001 | -,105 | -,032 |
|  | Age | ,014 | ,005 | ,387 | 2,973 | ,005 | ,005 | ,024 |
|  | LDL_C_HDL_C_ratio | ,371 | ,318 | ,151 | 1,166 | ,251 | -,273 | 1,016 |
| a. Dependent Variable: WCST_PE | | | | | | | | |

| **Model Summary** | | | | | | | | | |
| --- | --- | --- | --- | --- | --- | --- | --- | --- | --- |
| Model | R | R Square | Adjusted R Square | Std. Error of the Estimate | Change Statistics | | | | |
|  |  |  |  |  | R Square Change | F Change | df1 | df2 | Sig. F Change |
| 1 | ,444^a^ | ,197 | ,177 | ,32426 | ,197 | 9,827 | 1 | 40 | ,003 |
| 2 | ,606^b^ | ,368 | ,335 | ,29147 | ,170 | 10,506 | 1 | 39 | ,002 |
| 3 | ,635^c^ | ,403 | ,356 | ,28679 | ,036 | 2,284 | 1 | 38 | ,139 |
| a. Predictors: (Constant), Education | | | | | | | | | |
| b. Predictors: (Constant), Education, Age | | | | | | | | | |
| c. Predictors: (Constant), Education, Age, TC_HDL_C_ratio | | | | | | | | | |

| **Coefficients^a^** | | | | | | | | |
| --- | --- | --- | --- | --- | --- | --- | --- | --- |
| Model | | Unstandardized Coefficients | | Standardized Coefficients | t | Sig. | 95,0% Confidence Interval for B | |
|  |  | B | Std. Error | Beta |  |  | Lower Bound | Upper Bound |
| 1 | (Constant) | 1,860 | ,246 |  | 7,568 | ,000 | 1,363 | 2,357 |
|  | Education | -,062 | ,020 | -,444 | -3,135 | ,003 | -,103 | -,022 |
| 2 | (Constant) | 1,408 | ,261 |  | 5,386 | ,000 | ,879 | 1,936 |
|  | Education | -,070 | ,018 | -,496 | -3,867 | ,000 | -,106 | -,033 |
|  | Age | ,015 | ,005 | ,416 | 3,241 | ,002 | ,006 | ,025 |
| 3 | (Constant) | 1,106 | ,325 |  | 3,402 | ,002 | ,448 | 1,765 |
|  | Education | -,067 | ,018 | -,480 | -3,783 | ,001 | -,103 | -,031 |
|  | Age | ,014 | ,005 | ,379 | 2,947 | ,005 | ,004 | ,023 |
|  | TC_HDL_C_ratio | ,629 | ,416 | ,193 | 1,511 | ,139 | -,213 | 1,470 |
| a. Dependent Variable: WCST_PE | | | | | | | | |

| **Model Summary** | | | | | | | | | |
| --- | --- | --- | --- | --- | --- | --- | --- | --- | --- |
| Model | R | R Square | Adjusted R Square | Std. Error of the Estimate | Change Statistics | | | | |
|  |  |  |  |  | R Square Change | F Change | df1 | df2 | Sig. F Change |
| 1 | ,417^a^ | ,174 | ,153 | ,29313 | ,174 | 8,423 | 1 | 40 | ,006 |
| 2 | ,419^b^ | ,175 | ,133 | ,29660 | ,001 | ,069 | 1 | 39 | ,794 |
| a. Predictors: (Constant), Education | | | | | | | | | |
| b. Predictors: (Constant), Education, Total_cholesterol | | | | | | | | | |

| **Coefficients^a^** | | | | | | | | |
| --- | --- | --- | --- | --- | --- | --- | --- | --- |
| Model | | Unstandardized Coefficients | | Standardized Coefficients | t | Sig. | 95,0% Confidence Interval for B | |
|  |  | B | Std. Error | Beta |  |  | Lower Bound | Upper Bound |
| 1 | (Constant) | 1,701 | ,222 |  | 7,655 | ,000 | 1,252 | 2,150 |
|  | Education | -,052 | ,018 | -,417 | -2,902 | ,006 | -,089 | -,016 |
| 2 | (Constant) | 1,755 | ,306 |  | 5,740 | ,000 | 1,137 | 2,374 |
|  | Education | -,052 | ,018 | -,415 | -2,851 | ,007 | -,089 | -,015 |
|  | Total_cholesterol | -,011 | ,043 | -,038 | -,263 | ,794 | -,098 | ,076 |
| a. Dependent Variable: WCST_NPE | | | | | | | | |

| **Model Summary** | | | | | | | | | |
| --- | --- | --- | --- | --- | --- | --- | --- | --- | --- |
| Model | R | R Square | Adjusted R Square | Std. Error of the Estimate | Change Statistics | | | | |
|  |  |  |  |  | R Square Change | F Change | df1 | df2 | Sig. F Change |
| 1 | ,417^a^ | ,174 | ,153 | ,29313 | ,174 | 8,423 | 1 | 40 | ,006 |
| 2 | ,422^b^ | ,178 | ,136 | ,29618 | ,004 | ,181 | 1 | 39 | ,673 |
| a. Predictors: (Constant), Education | | | | | | | | | |
| b. Predictors: (Constant), Education, Triglycerides | | | | | | | | | |

| **Coefficients^a^** | | | | | | | | |
| --- | --- | --- | --- | --- | --- | --- | --- | --- |
| Model | | Unstandardized Coefficients | | Standardized Coefficients | t | Sig. | 95,0% Confidence Interval for B | |
|  |  | B | Std. Error | Beta |  |  | Lower Bound | Upper Bound |
| 1 | (Constant) | 1,701 | ,222 |  | 7,655 | ,000 | 1,252 | 2,150 |
|  | Education | -,052 | ,018 | -,417 | -2,902 | ,006 | -,089 | -,016 |
| 2 | (Constant) | 1,696 | ,225 |  | 7,544 | ,000 | 1,241 | 2,151 |
|  | Education | -,052 | ,018 | -,418 | -2,875 | ,007 | -,089 | -,015 |
|  | Triglycerides | ,104 | ,244 | ,062 | ,425 | ,673 | -,390 | ,598 |
| a. Dependent Variable: WCST_NPE | | | | | | | | |

| **Model Summary** | | | | | | | | | |
| --- | --- | --- | --- | --- | --- | --- | --- | --- | --- |
| Model | R | R Square | Adjusted R Square | Std. Error of the Estimate | Change Statistics | | | | |
|  |  |  |  |  | R Square Change | F Change | df1 | df2 | Sig. F Change |
| 1 | ,417^a^ | ,174 | ,153 | ,29313 | ,174 | 8,423 | 1 | 40 | ,006 |
| 2 | ,449^b^ | ,201 | ,160 | ,29191 | ,027 | 1,335 | 1 | 39 | ,255 |
| a. Predictors: (Constant), Education | | | | | | | | | |
| b. Predictors: (Constant), Education, HDL_cholesterol | | | | | | | | | |

| **Coefficients^a^** | | | | | | | | |
| --- | --- | --- | --- | --- | --- | --- | --- | --- |
| Model | | Unstandardized Coefficients | | Standardized Coefficients | t | Sig. | 95,0% Confidence Interval for B | |
|  |  | B | Std. Error | Beta |  |  | Lower Bound | Upper Bound |
| 1 | (Constant) | 1,701 | ,222 |  | 7,655 | ,000 | 1,252 | 2,150 |
|  | Education | -,052 | ,018 | -,417 | -2,902 | ,006 | -,089 | -,016 |
| 2 | (Constant) | 1,899 | ,280 |  | 6,783 | ,000 | 1,333 | 2,465 |
|  | Education | -,050 | ,018 | -,397 | -2,753 | ,009 | -,086 | -,013 |
|  | HDL_cholesterol | -,145 | ,125 | -,167 | -1,156 | ,255 | -,398 | ,109 |
| a. Dependent Variable: WCST_NPE | | | | | | | | |

| **Model Summary** | | | | | | | | | |
| --- | --- | --- | --- | --- | --- | --- | --- | --- | --- |
| Model | R | R Square | Adjusted R Square | Std. Error of the Estimate | Change Statistics | | | | |
|  |  |  |  |  | R Square Change | F Change | df1 | df2 | Sig. F Change |
| 1 | ,417^a^ | ,174 | ,153 | ,29313 | ,174 | 8,423 | 1 | 40 | ,006 |
| 2 | ,419^b^ | ,175 | ,133 | ,29663 | ,001 | ,061 | 1 | 39 | ,806 |
| a. Predictors: (Constant), Education | | | | | | | | | |
| b. Predictors: (Constant), Education, LDL_cholesterol | | | | | | | | | |

| **Coefficients^a^** | | | | | | | | |
| --- | --- | --- | --- | --- | --- | --- | --- | --- |
| Model | | Unstandardized Coefficients | | Standardized Coefficients | t | Sig. | 95,0% Confidence Interval for B | |
|  |  | B | Std. Error | Beta |  |  | Lower Bound | Upper Bound |
| 1 | (Constant) | 1,701 | ,222 |  | 7,655 | ,000 | 1,252 | 2,150 |
|  | Education | -,052 | ,018 | -,417 | -2,902 | ,006 | -,089 | -,016 |
| 2 | (Constant) | 1,741 | ,277 |  | 6,276 | ,000 | 1,180 | 2,302 |
|  | Education | -,052 | ,018 | -,415 | -2,854 | ,007 | -,089 | -,015 |
|  | LDL_cholesterol | -,014 | ,058 | -,036 | -,248 | ,806 | -,133 | ,104 |
| a. Dependent Variable: WCST_NPE | | | | | | | | |

| **Model Summary** | | | | | | | | | |
| --- | --- | --- | --- | --- | --- | --- | --- | --- | --- |
| Model | R | R Square | Adjusted R Square | Std. Error of the Estimate | Change Statistics | | | | |
|  |  |  |  |  | R Square Change | F Change | df1 | df2 | Sig. F Change |
| 1 | ,417^a^ | ,174 | ,153 | ,29313 | ,174 | 8,423 | 1 | 40 | ,006 |
| 2 | ,430^b^ | ,185 | ,143 | ,29487 | ,011 | ,529 | 1 | 39 | ,471 |
| a. Predictors: (Constant), Education | | | | | | | | | |
| b. Predictors: (Constant), Education, LDL_C_HDL_C_ratio | | | | | | | | | |

| **Coefficients^a^** | | | | | | | | |
| --- | --- | --- | --- | --- | --- | --- | --- | --- |
| Model | | Unstandardized Coefficients | | Standardized Coefficients | t | Sig. | 95,0% Confidence Interval for B | |
|  |  | B | Std. Error | Beta |  |  | Lower Bound | Upper Bound |
| 1 | (Constant) | 1,701 | ,222 |  | 7,655 | ,000 | 1,252 | 2,150 |
|  | Education | -,052 | ,018 | -,417 | -2,902 | ,006 | -,089 | -,016 |
| 2 | (Constant) | 1,633 | ,242 |  | 6,742 | ,000 | 1,143 | 2,123 |
|  | Education | -,052 | ,018 | -,413 | -2,855 | ,007 | -,088 | -,015 |
|  | LDL_C_HDL_C_ratio | ,231 | ,318 | ,105 | ,727 | ,471 | -,412 | ,874 |
| a. Dependent Variable: WCST_NPE | | | | | | | | |

| **Model Summary** | | | | | | | | | |
| --- | --- | --- | --- | --- | --- | --- | --- | --- | --- |
| Model | R | R Square | Adjusted R Square | Std. Error of the Estimate | Change Statistics | | | | |
|  |  |  |  |  | R Square Change | F Change | df1 | df2 | Sig. F Change |
| 1 | ,417^a^ | ,174 | ,153 | ,29313 | ,174 | 8,423 | 1 | 40 | ,006 |
| 2 | ,442^b^ | ,195 | ,154 | ,29304 | ,021 | 1,025 | 1 | 39 | ,318 |
| a. Predictors: (Constant), Education | | | | | | | | | |
| b. Predictors: (Constant), Education, TC_HDL_C_ratio | | | | | | | | | |

| **Coefficients^a^** | | | | | | | | |
| --- | --- | --- | --- | --- | --- | --- | --- | --- |
| Model | | Unstandardized Coefficients | | Standardized Coefficients | t | Sig. | 95,0% Confidence Interval for B | |
|  |  | B | Std. Error | Beta |  |  | Lower Bound | Upper Bound |
| 1 | (Constant) | 1,701 | ,222 |  | 7,655 | ,000 | 1,252 | 2,150 |
|  | Education | -,052 | ,018 | -,417 | -2,902 | ,006 | -,089 | -,016 |
| 2 | (Constant) | 1,472 | ,317 |  | 4,638 | ,000 | ,830 | 2,113 |
|  | Education | -,051 | ,018 | -,408 | -2,834 | ,007 | -,088 | -,015 |
|  | TC_HDL_C_ratio | ,422 | ,417 | ,146 | 1,012 | ,318 | -,422 | 1,266 |
| a. Dependent Variable: WCST_NPE | | | | | | | | |

| **Model Summary** | | | | | | | | | |
| --- | --- | --- | --- | --- | --- | --- | --- | --- | --- |
| Model | R | R Square | Adjusted R Square | Std. Error of the Estimate | Change Statistics | | | | |
|  |  |  |  |  | R Square Change | F Change | df1 | df2 | Sig. F Change |
| 1 | ,416^a^ | ,173 | ,153 | 12,30881 | ,173 | 8,389 | 1 | 40 | ,006 |
| 2 | ,444^b^ | ,197 | ,156 | 12,28736 | ,023 | 1,140 | 1 | 39 | ,292 |
| a. Predictors: (Constant), Depression_severity | | | | | | | | | |
| b. Predictors: (Constant), Depression_severity, Total_cholesterol | | | | | | | | | |

| **Coefficients^a^** | | | | | | | | |
| --- | --- | --- | --- | --- | --- | --- | --- | --- |
| Model | | Unstandardized Coefficients | | Standardized Coefficients | t | Sig. | 95,0% Confidence Interval for B | |
|  |  | B | Std. Error | Beta |  |  | Lower Bound | Upper Bound |
| 1 | (Constant) | 74,783 | 4,699 |  | 15,914 | ,000 | 65,286 | 84,281 |
|  | Depression_severity | -,536 | ,185 | -,416 | -2,896 | ,006 | -,911 | -,162 |
| 2 | (Constant) | 84,748 | 10,447 |  | 8,113 | ,000 | 63,618 | 105,879 |
|  | Depression_severity | -,548 | ,185 | -,426 | -2,961 | ,005 | -,923 | -,174 |
|  | Total_cholesterol | -1,900 | 1,780 | -,153 | -1,068 | ,292 | -5,501 | 1,700 |
| a. Dependent Variable: WCST_CLR | | | | | | | | |

| **Model Summary** | | | | | | | | | |
| --- | --- | --- | --- | --- | --- | --- | --- | --- | --- |
| Model | R | R Square | Adjusted R Square | Std. Error of the Estimate | Change Statistics | | | | |
|  |  |  |  |  | R Square Change | F Change | df1 | df2 | Sig. F Change |
| 1 | ,416^a^ | ,173 | ,153 | 12,30881 | ,173 | 8,389 | 1 | 40 | ,006 |
| 2 | ,489^b^ | ,239 | ,200 | 11,95784 | ,066 | 3,382 | 1 | 39 | ,074 |
| a. Predictors: (Constant), Depression_severity | | | | | | | | | |
| b. Predictors: (Constant), Depression_severity, Triglycerides | | | | | | | | | |

| **Coefficients^a^** | | | | | | | | |
| --- | --- | --- | --- | --- | --- | --- | --- | --- |
| Model | | Unstandardized Coefficients | | Standardized Coefficients | t | Sig. | 95,0% Confidence Interval for B | |
|  |  | B | Std. Error | Beta |  |  | Lower Bound | Upper Bound |
| 1 | (Constant) | 74,783 | 4,699 |  | 15,914 | ,000 | 65,286 | 84,281 |
|  | Depression_severity | -,536 | ,185 | -,416 | -2,896 | ,006 | -,911 | -,162 |
| 2 | (Constant) | 73,750 | 4,600 |  | 16,034 | ,000 | 64,447 | 83,054 |
|  | Depression_severity | -,448 | ,186 | -,348 | -2,409 | ,021 | -,825 | -,072 |
|  | Triglycerides | -18,774 | 10,208 | -,266 | -1,839 | ,074 | -39,421 | 1,874 |
| a. Dependent Variable: WCST_CLR | | | | | | | | |

| **Model Summary** | | | | | | | | | |
| --- | --- | --- | --- | --- | --- | --- | --- | --- | --- |
| Model | R | R Square | Adjusted R Square | Std. Error of the Estimate | Change Statistics | | | | |
|  |  |  |  |  | R Square Change | F Change | df1 | df2 | Sig. F Change |
| 1 | ,416^a^ | ,173 | ,153 | 12,30881 | ,173 | 8,389 | 1 | 40 | ,006 |
| 2 | ,452^b^ | ,204 | ,164 | 12,22876 | ,031 | 1,525 | 1 | 39 | ,224 |
| a. Predictors: (Constant), Depression_severity | | | | | | | | | |
| b. Predictors: (Constant), Depression_severity, HDL_cholesterol | | | | | | | | | |

| **Coefficients^a^** | | | | | | | | |
| --- | --- | --- | --- | --- | --- | --- | --- | --- |
| Model | | Unstandardized Coefficients | | Standardized Coefficients | t | Sig. | 95,0% Confidence Interval for B | |
|  |  | B | Std. Error | Beta |  |  | Lower Bound | Upper Bound |
| 1 | (Constant) | 74,783 | 4,699 |  | 15,914 | ,000 | 65,286 | 84,281 |
|  | Depression_severity | -,536 | ,185 | -,416 | -2,896 | ,006 | -,911 | -,162 |
| 2 | (Constant) | 61,451 | 11,761 |  | 5,225 | ,000 | 37,661 | 85,240 |
|  | Depression_severity | -,439 | ,200 | -,341 | -2,191 | ,035 | -,844 | -,034 |
|  | HDL_cholesterol | 7,008 | 5,674 | ,192 | 1,235 | ,224 | -4,469 | 18,486 |
| a. Dependent Variable: WCST_CLR | | | | | | | | |

| **Model Summary** | | | | | | | | | |
| --- | --- | --- | --- | --- | --- | --- | --- | --- | --- |
| Model | R | R Square | Adjusted R Square | Std. Error of the Estimate | Change Statistics | | | | |
|  |  |  |  |  | R Square Change | F Change | df1 | df2 | Sig. F Change |
| 1 | ,416^a^ | ,173 | ,153 | 12,30881 | ,173 | 8,389 | 1 | 40 | ,006 |
| 2 | ,481^b^ | ,232 | ,192 | 12,01796 | ,058 | 2,960 | 1 | 39 | ,093 |
| a. Predictors: (Constant), Depression_severity | | | | | | | | | |
| b. Predictors: (Constant), Depression_severity, LDL_cholesterol | | | | | | | | | |

| **Coefficients^a^** | | | | | | | | |
| --- | --- | --- | --- | --- | --- | --- | --- | --- |
| Model | | Unstandardized Coefficients | | Standardized Coefficients | t | Sig. | 95,0% Confidence Interval for B | |
|  |  | B | Std. Error | Beta |  |  | Lower Bound | Upper Bound |
| 1 | (Constant) | 74,783 | 4,699 |  | 15,914 | ,000 | 65,286 | 84,281 |
|  | Depression_severity | -,536 | ,185 | -,416 | -2,896 | ,006 | -,911 | -,162 |
| 2 | (Constant) | 86,688 | 8,303 |  | 10,441 | ,000 | 69,894 | 103,481 |
|  | Depression_severity | -,532 | ,181 | -,413 | -2,943 | ,005 | -,898 | -,166 |
|  | LDL_cholesterol | -4,066 | 2,364 | -,241 | -1,720 | ,093 | -8,847 | ,715 |
| a. Dependent Variable: WCST_CLR | | | | | | | | |

| **Model Summary** | | | | | | | | | |
| --- | --- | --- | --- | --- | --- | --- | --- | --- | --- |
| Model | R | R Square | Adjusted R Square | Std. Error of the Estimate | Change Statistics | | | | |
|  |  |  |  |  | R Square Change | F Change | df1 | df2 | Sig. F Change |
| 1 | ,416^a^ | ,173 | ,153 | 12,30881 | ,173 | 8,389 | 1 | 40 | ,006 |
| 2 | ,528^b^ | ,279 | ,242 | 11,64060 | ,106 | 5,724 | 1 | 39 | ,022 |
| a. Predictors: (Constant), Depression_severity | | | | | | | | | |
| b. Predictors: (Constant), Depression_severity, LDL_C_HDL_C_ratio | | | | | | | | | |

| **Coefficients^a^** | | | | | | | | |
| --- | --- | --- | --- | --- | --- | --- | --- | --- |
| Model | | Unstandardized Coefficients | | Standardized Coefficients | t | Sig. | 95,0% Confidence Interval for B | |
|  |  | B | Std. Error | Beta |  |  | Lower Bound | Upper Bound |
| 1 | (Constant) | 74,783 | 4,699 |  | 15,914 | ,000 | 65,286 | 84,281 |
|  | Depression_severity | -,536 | ,185 | -,416 | -2,896 | ,006 | -,911 | -,162 |
| 2 | (Constant) | 80,241 | 4,995 |  | 16,063 | ,000 | 70,137 | 90,346 |
|  | Depression_severity | -,411 | ,183 | -,319 | -2,247 | ,030 | -,781 | -,041 |
|  | LDL_C_HDL_C_ratio | -31,298 | 13,082 | -,340 | -2,393 | ,022 | -57,759 | -4,838 |
| a. Dependent Variable: WCST_CLR | | | | | | | | |

| **Model Summary** | | | | | | | | | |
| --- | --- | --- | --- | --- | --- | --- | --- | --- | --- |
| Model | R | R Square | Adjusted R Square | Std. Error of the Estimate | Change Statistics | | | | |
|  |  |  |  |  | R Square Change | F Change | df1 | df2 | Sig. F Change |
| 1 | ,416^a^ | ,173 | ,153 | 12,30881 | ,173 | 8,389 | 1 | 40 | ,006 |
| 2 | ,510^b^ | ,260 | ,222 | 11,79393 | ,087 | 4,569 | 1 | 39 | ,039 |
| a. Predictors: (Constant), Depression_severity | | | | | | | | | |
| b. Predictors: (Constant), Depression_severity, TC_HDL_C_ratio | | | | | | | | | |

| **Coefficients^a^** | | | | | | | | |
| --- | --- | --- | --- | --- | --- | --- | --- | --- |
| Model | | Unstandardized Coefficients | | Standardized Coefficients | t | Sig. | 95,0% Confidence Interval for B | |
|  |  | B | Std. Error | Beta |  |  | Lower Bound | Upper Bound |
| 1 | (Constant) | 74,783 | 4,699 |  | 15,914 | ,000 | 65,286 | 84,281 |
|  | Depression_severity | -,536 | ,185 | -,416 | -2,896 | ,006 | -,911 | -,162 |
| 2 | (Constant) | 91,170 | 8,891 |  | 10,254 | ,000 | 73,187 | 109,154 |
|  | Depression_severity | -,416 | ,186 | -,323 | -2,233 | ,031 | -,792 | -,039 |
|  | TC_HDL_C_ratio | -37,586 | 17,585 | -,309 | -2,137 | ,039 | -73,154 | -2,018 |
| a. Dependent Variable: WCST_CLR | | | | | | | | |
